# Synergism of dual AAV gene therapy and rapamycin rescues GSDIII phenotype in muscle and liver

**DOI:** 10.1172/jci.insight.172614

**Published:** 2024-05-16

**Authors:** Louisa Jauze, Mallaury Vie, Quentin Miagoux, Lucille Rossiaud, Patrice Vidal, Valle Montalvo-Romeral, Hanadi Saliba, Margot Jarrige, Helene Polveche, Justine Nozi, Pierre-Romain Le Brun, Luca Bocchialini, Amandine Francois, Jérémie Cosette, Jérémy Rouillon, Fanny Collaud, Fanny Bordier, Emilie Bertil-Froidevaux, Christophe Georger, Laetitia van Wittenberghe, Adeline Miranda, Nathalie F. Daniele, David-Alexandre Gross, Lucile Hoch, Xavier Nissan, Giuseppe Ronzitti

**Affiliations:** 1 Généthon, Évry, France.; 2 Université Paris-Saclay, Univ Évry, Inserm, Généthon, Integrare research unit UMR_S951, 91000 Évry, France.; 3 CECS, I-STEM, Institute for Stem Cell Therapy and Exploration of Monogenic Diseases, Corbeil-Essonnes, France.

**Keywords:** Therapeutics, Autophagy, Gene therapy, Skeletal muscle

## Abstract

Glycogen storage disease type III (GSDIII) is a rare metabolic disorder due to glycogen debranching enzyme (GDE) deficiency. Reduced GDE activity leads to pathological glycogen accumulation responsible for impaired hepatic metabolism and muscle weakness. To date, there is no curative treatment for GSDIII. We previously reported that 2 distinct dual AAV vectors encoding for GDE were needed to correct liver and muscle in a GSDIII mouse model. Here, we evaluated the efficacy of rapamycin in combination with AAV gene therapy. Simultaneous treatment with rapamycin and a potentially novel dual AAV vector expressing GDE in the liver and muscle resulted in a synergic effect demonstrated at biochemical and functional levels. Transcriptomic analysis confirmed synergy and suggested a putative mechanism based on the correction of lysosomal impairment. In GSDIII mice livers, dual AAV gene therapy combined with rapamycin reduced the effect of the immune response to AAV observed in this disease model. These data provide proof of concept of an approach exploiting the combination of gene therapy and rapamycin to improve efficacy and safety and to support clinical translation.

## Abstract 2

Simultaneous treatment of a murine GSDIII model with rapamycin and a GDE-expressing, liver- and muscle-targeting AAV vector resulted in a synergic effect at the biochemical and functional level.

## Introduction

Glycogen storage disease type III (GSDIII) is a rare, autosomal recessive disorder with an incidence of 1 in 100,000 live births due to mutations in the amylo-α-1, 6-glucosidase, 4-α-glucanotransferase (*AGL*) gene. *AGL* encodes for the glycogen debranching enzyme (GDE). Its deficiency leads to glycogen accumulation mainly in liver, heart, and skeletal muscles. Inactivating mutations on the *AGL* gene result in impaired liver metabolism with severe hypoglycemia, heart function degradation, and progressive muscle weakness (1).

The disease evolves in 2 phases: (a) in childhood, the main manifestation is the metabolic disorder with hepatomegaly, fasting hypoglycemia, hyperlipidemia, and hyperketonemia; (b) with aging, a progressive myopathy characterized by skeletal muscle weakness and exercise intolerance appears (2) together with a progressive liver disease (1, 3, 4). Patients with GSDIII also present cardiac involvement since early ages, in some cases associated with cardiomyopathy (2, 5).

To date, there is no curative treatment for GSDIII. A dietary regimen including frequent meals and a strict diet enriched in uncooked cornstarch, while controlling hypoglycemia episodes and reducing glycogen accumulation, fails at addressing the muscle impairment (1, 2, 6). A high-protein diet was sporadically reported to be beneficial in reducing or stabilizing skeletal and cardiac muscle manifestations (7–12).

The unmet medical need and the fact that GSDIII has a monogenic origin make this disease an ideal target for gene replacement therapy. Adeno-associated virus–derived (AAV-derived) vectors demonstrated efficient targeting of liver and muscle in multiple glycogenosis preclinical models (13–21). The limited packaging capacity of AAVs has been extended by demonstrating the feasibility of the use of 2 overlapping vectors encoding GDE in a mouse model of GSDIII (22).

Recently, an orthogonal approach was described based on a single AAV vector encoding a bacterial GDE named pullulanase (23). When expressed under the control of a CMV enhancer/chicken β-actin promoter (CB), pullulanase corrected glycogen accumulation in skeletal muscle and heart but not in liver of a GSDIII mouse model. Injection of a second vector expressing pullulanase under the control of a liver-specific promoter (LSP) in the same animals was necessary to correct the hepatic glycogen accumulation and reverse the hepatic fibrosis (23). However, the expression of pullulanase was lost after a few weeks, probably because of transgene-induced cytotoxic T lymphocyte (24). To address this issue, a vector encoding pullulanase under a tandem promoter CB-LSP was assessed (24). The CB-LSP-pullulanase vector corrected glycogen accumulation in liver, heart, and quadriceps; reversed hepatic fibrosis; and decreased pullulanase-induced CTL response in GSDIII mice (24). Although promising, these data confirm the potential risk of immune responses to bacterial transgenes (25) that may preclude a clinical translation of this approach.

Here, in an effort to optimize the dual vector approach for GSDIII, we attempted the use of a recently developed tandem promoter expressing GDE in liver and muscles (AAV9-LiMP-GDE overlapping [AAV9-LiMP-GDEov]). Despite transient rescue obtained after 1 month of treatment, the correction was lost over time. To overcome this issue, and because rapamycin was shown to be promising for GSDIII treatment in a dog model of GSDIII (26), we assessed the efficiency of rapamycin alone and in combination with AAV9-LiMP-GDEov in a murine model of the disease. One-month treatment with rapamycin alone was not efficient in correcting the hepatic and muscular impairment in our mouse model. However, the same rapamycin treatment followed by the injection of AAV9-LiMP-GDEov had a synergic effect with gene therapy, increasing glycogen clearance in skeletal muscles and improving muscle strength rescue as compared with AAV9-LiMP-GDEov alone. This synergic effect was confirmed also by analyzing the RNA expression profile in triceps. In general, we observed a better rescue of the disease markers in animals treated with the combination of rapamycin and gene therapy. More precisely, the lysosomal pathway was better corrected in triceps of GSDIII mice treated with the combination therapy. Interestingly, transcriptomic analysis performed in the liver showed, in animals receiving the AAV9-LiMP-GDE vector, an RNA expression pattern suggestive of an immune response to gene therapy that was controlled by rapamycin treatment.

## Results

### Long-term partial correction of the muscular and hepatic GSDIII phenotype in Agl^–/–^ mice with a dual AAV vector expressing GDE under the control of a tandem LiMP.

We have previously established proof-of-concept of the correction of the muscular and hepatic disease manifestations in *Agl^–/–^* mice with 2 distinct overlapping AAV vectors expressing GDE either under the control of a constitutive promoter or a LSP (22). Although the CAG promoter allowed efficient expression of the GDE transgene in liver and muscle with a dual AAV vector, the use of such a promoter was associated with an increased risk of immune response to the transgene (13, 27, 28) and the potential formation of liver tumors at high vector doses when injected in neonatal animals (29).

**Figure 1:**
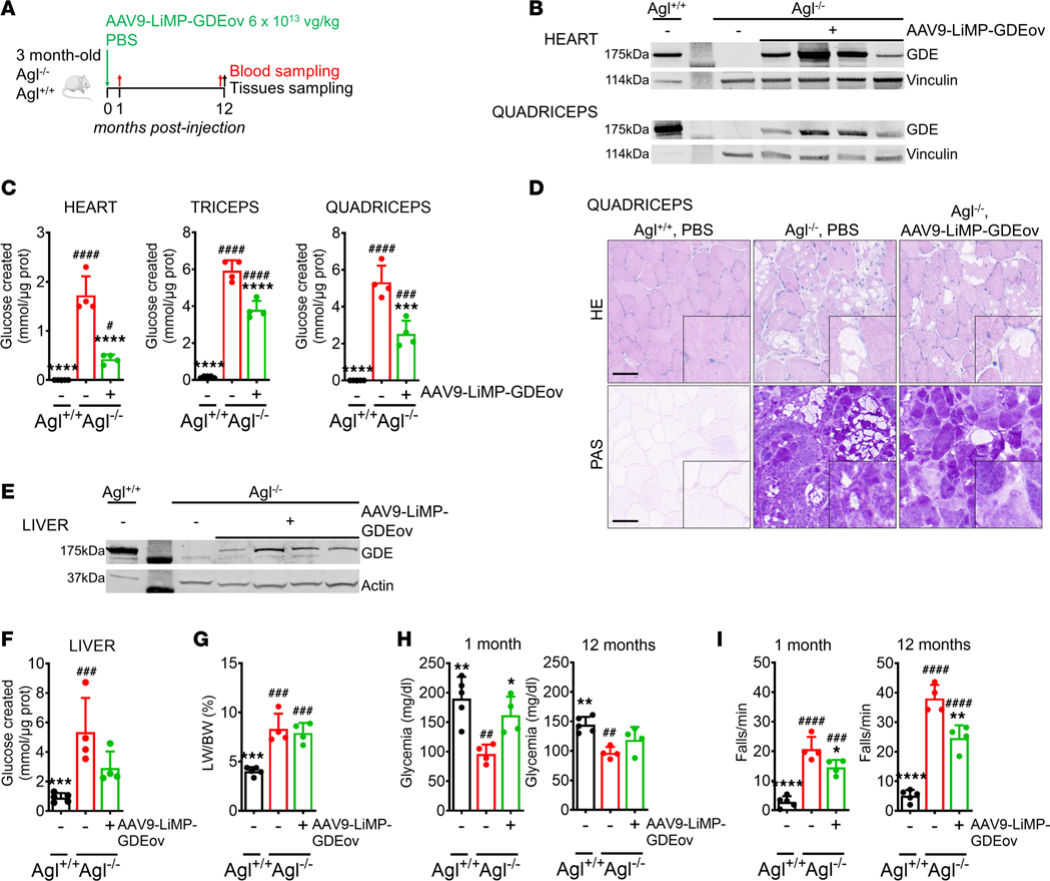
AAV9-LiMP-GDEov achieves partial correction of the muscles, but correction is lost over time in liver of *Agl^–/–^* mice. (**A**) Three-month-old *Agl*^–/–^ mice received a single injection of AAV9-LiMP-GDEov at 6 × 10^13^ vg/kg total (*n* = 4 per group) and followed up for 12 months. PBS-injected *Agl^+/+^* (*n* = 5) and *Agl^−/−^* (*n* = 4) mice were used as controls. (**B**) Western blot analysis of GDE in heart and quadriceps. (**C**) Glycogen content in heart, quadriceps, and triceps 12 months after vector injection. (**D**) H&E and Periodic Acid Schiff (PAS) staining performed in quadriceps sections. Scale bar: 100 μm. (**E**) Western blot analysis of GDE in liver. (**F**) Glycogen content in liver 12 months after vector injection. (**G**) Liver weight expressed as a percentage of body weight measured 12 months after vector injection. (**H**) Glycemia measured 1 month or 12 months after vector injection. (**I**) Wire hang test shown as number of falls per minute performed 1 month or 12 months after vector injection. Data are shown as mean ± SD. Statistical analyses were performed by 1-way ANOVA with Tukey post hoc test. Significance was indicated with * vs. *Agl^−/−^* and ^#^ vs. *Agl^+/+^*. * and ^#^*P* < 0.05, ** and ^##^*P* < 0.01, *** and ^###^*P* < 0.001, and **** and ^####^*P* < 0.0001.

To overcome these limitations and achieve safe and efficient gene transfer in both liver and muscle, we took advantage of a recently developed tandem liver-muscle promoter (LiMP), which allows us to control immune responses to the transgene while achieving a robust and specific expression in liver and muscle (13). Three-month-old *Agl^–/–^* mice received 6 × 10^13^ vg/kg of a dual AAV9 vector expressing the human GDE transgene under the control of the LiMP (AAV9-LiMP-GDEov) and followed up for 12 months (Figure 1A). At sacrifice, Western blot analysis showed expression of GDE in heart and quadriceps (Figure 1B). In the heart, the robust GDE expression, likely due to the high tissue transduction achieved with AAV9 (Supplemental Table 1; supplemental material available online with this article; https://doi.org/10.1172/jci.insight.172614DS1), resulted in a partial correction of glycogen accumulation and histological features as measured biochemically and by H&E and periodic-acid–Schiff (PAS) staining (Figure 1C and Supplemental Figure 1A). The lower transduction levels (Supplemental Table 1) and GDE protein expression (Figure 1B) correlated with partial correction of glycogen accumulation in quadriceps and triceps (Figure 1C). Those results were confirmed by H&E and PAS staining that showed an only partial rescue of the histological features in quadriceps (Figure 1D). In liver, GDE transgene expression (Figure 1E) resulted in partial correction of glycogen accumulation (Figure 1F), with little to no effect on fibrosis or other histological features (Supplemental Figure 1B). Accordingly, at sacrifice, hepatomegaly was not corrected (Figure 1G). Surprisingly, 1 month after treatment of *Agl^–/–^* mice with AAV9-LiMP-GDEov, glycemia was completely corrected, whereas 12 months after vector injection, the glycemia was no longer rescued (Figure 1H), confirming the biochemical data and supporting the hypothesis of a loss of correction in the liver. Evaluation of muscle function by wire-hang revealed a partial correction of the muscle strength impairment at both 1 and 12 months after AAV9-LiMP-GDEov administration (Figure 1I), suggesting stability of gene transfer over 12 months. Importantly, in mice treated with AAV9-LiMP-GDEov, variable levels of anti-GDE antibodies were measured during the protocol that were not correlated with transgene expression levels in liver and muscle (Supplemental Figure 1C).

To conclude, long-term evaluation of the efficacy of an AAV gene therapy approach for GSDIII based on the use of a promoter with dual liver muscle specificity indicates partial rescue of muscle impairment and loss of efficacy in liver, thus supporting the need for optimization to achieve full correction of GSDIII disease manifestations while reducing antitransgene immunogenicity.

### Pharmacological treatment with rapamycin alone does not correct the hepatic and muscular phenotype of Agl^–/–^ mice.

**Figure 2:**
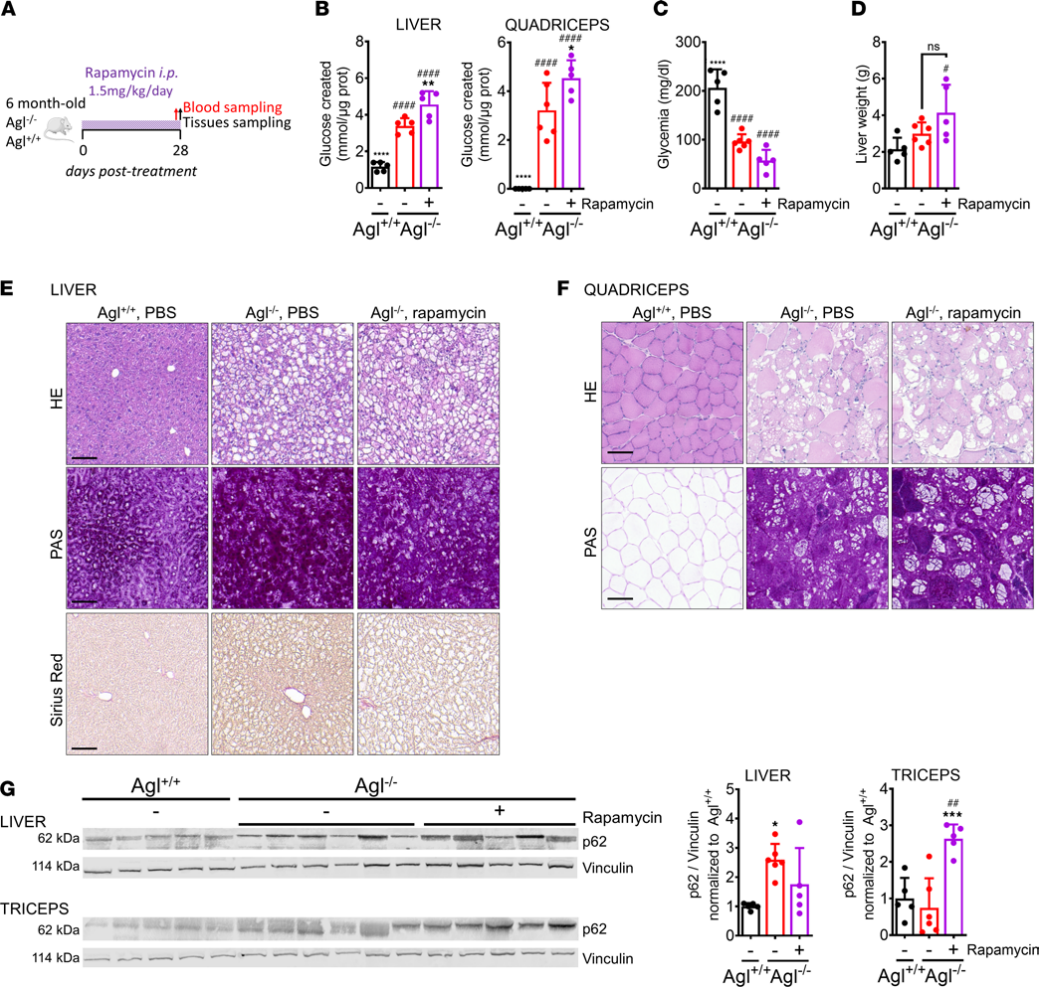
One-month daily rapamycin treatment fails at rescuing liver and muscle impairment in *Agl^–/–^* mice. (**A**) Six-month-old *Agl^−/−^* mice received daily i.p. injections of rapamycin at 1.5 mg/kg for a month (*n* = 5 per group). Vehicle-injected *Agl^+/+^* (*n* = 5) and *Agl^−/−^* (*n* = 5) mice were used as controls. (**B**) Glycogen content measured in liver and quadriceps at the end of the treatment. (**C**) Glycemia measured at the end of the treatment. (**D**) Liver weight measured at the end of the treatment. (**E**) H&E, Periodic Acid Schiff (PAS), and Sirius red staining performed in liver sections. Scale bar: 100 μm. (**F**) H&E and Periodic Acid Schiff (PAS) staining performed in quadriceps sections. Scale bar: 100 μm. (**G**) Western blot analysis of p62 in liver and triceps. Quantifications of p62 proteins bands are plotted next to the Western blot and expressed as ratio to vinculin. Data shown as mean ± SD. Statistical analyses were performed by 1-way ANOVA with Tukey post hoc test. Significance was indicated with * vs. *Agl^–/–^* and ^#^ vs. *Agl^+/+^*. * and ^#^*P* < 0.05, ** and ^##^*P* < 0.01, ***P < 0.001, and **** and ^####^*P* < 0.0001.

Inhibition of the mammalian target of rapamycin (mTOR) leads to increased autophagy in multiple cell types. Inhibition of mTOR in immune cells is associated with decreased activation and immunosuppression (30). Although, in GSDIII, the pathological glycogen accumulation occurs in the cytosol, the activation of glycogen-specific autophagy (glycophagy) may participate in glycogen clearance. Moreover, it has been recently shown that autophagy is impaired in GSDIII skeletal muscle biopsies (31). Treatment with rapamycin was shown effective in a dog model of GSDIII, correcting glycogen accumulation in liver and preventing glycogen accumulation in skeletal muscle (26). To confirm these results in a mouse model of GSDIII, we treated symptomatic, 6-month-old *Agl^–/–^* mice with 1.5 mg/kg/day for 1 month, a regimen known to achieve immunosuppression in rodents (32) (Figure 2A). After 1 month of daily treatment, we observed a slight increase in glycogen content in liver, heart, and skeletal muscles (Figure 2B and Supplemental Figure 2A). Together with no correction of hypoglycemia by rapamycin treatment (Figure 2C), we observed a tendency toward increased liver weight (Figure 2D). To rule out a potential toxicity due to rapamycin treatment, aspartate and alanine transaminases (AST and ALT) were measured in blood. The hepatic enzymes, elevated in *Agl^–/–^* mice, were not significantly increased by rapamycin treatment (Supplemental Figure 2, B and C). Liver histological features were similar between the two groups of *Agl^–/–^* mice regardless of the treatment (Figure 2E). Tendentially decreased CD8^+^ infiltrates were measured in rapamycin-treated *Agl^–/–^* mice by histology and CD8 mRNA analysis (Supplementary Figure 2, D–F).

The general architecture of heart and skeletal muscle evaluated by H&E and PAS staining showed signs of glycogen accumulation characteristic of the disease (Figure 2F and Supplemental Figure 2G).

Rapamycin is an mTOR inhibitor; however, in the context of Pompe disease, another GSD, it has been shown that rapamycin may inhibit glycogen synthase in muscle (33). To test whether rapamycin was acting through the same mechanism, we evaluated the effect of a 1-month treatment with the molecule on GSDIII mice. Gys1 inhibition was then assessed by measuring both total levels of Gys1, the muscle-specific glycogen synthase, and its Ser641 phosphorylation, a known inhibiting phosphorylation participating in the rapamycin effect observed in Pompe disease (33). We found that Gys1 was strongly inhibited in the triceps of *Agl^–/–^* mice while 1-month treatment with rapamycin had no effect (Supplemental Figure 2, H and I).

To evaluate the effect of rapamycin treatment on autophagy induction, we measured the levels of p62 protein, a known marker of autophagy in GSDs (31, 34, 35), in liver and muscle. In liver, p62 protein levels were increased in untreated *Agl^–/–^* mice compared with *Agl^+/+^*, and they only slightly decreased by rapamycin treatment (Figure 2G), suggesting a preexisting block of hepatic autophagy in *Agl^–/–^* mice. Surprisingly, p62 levels in triceps of untreated *Agl^–/–^* mice were similar to those of WT animals and were greatly increased by rapamycin treatment (Figure 2G). Measurement of GAA activity in the same tissue showed similar levels in *Agl^+/+^* and *Agl^–/–^* mice, and treatment with rapamycin had no effect (Supplemental Figure 2J). Our current working hypothesis to interpretate the data obtained in skeletal muscle is described in Supplemental Figure 3. We hypothesize that rapamycin treatment increases the autophagic flux in GSDIII muscle, leading to increased glycophagy. However, the lysosomal capacity to degrade glycogen remained constant, as shown by the stable GAA activity, and this led to an accumulation of autophagosomes and the consequent increased p62 levels suggestive of an autophagy block.

### Combination of rapamycin treatment with AAV9-LiMP-GDEov rescues liver and muscle phenotype in GSDIII mice.

**Figure 3:**
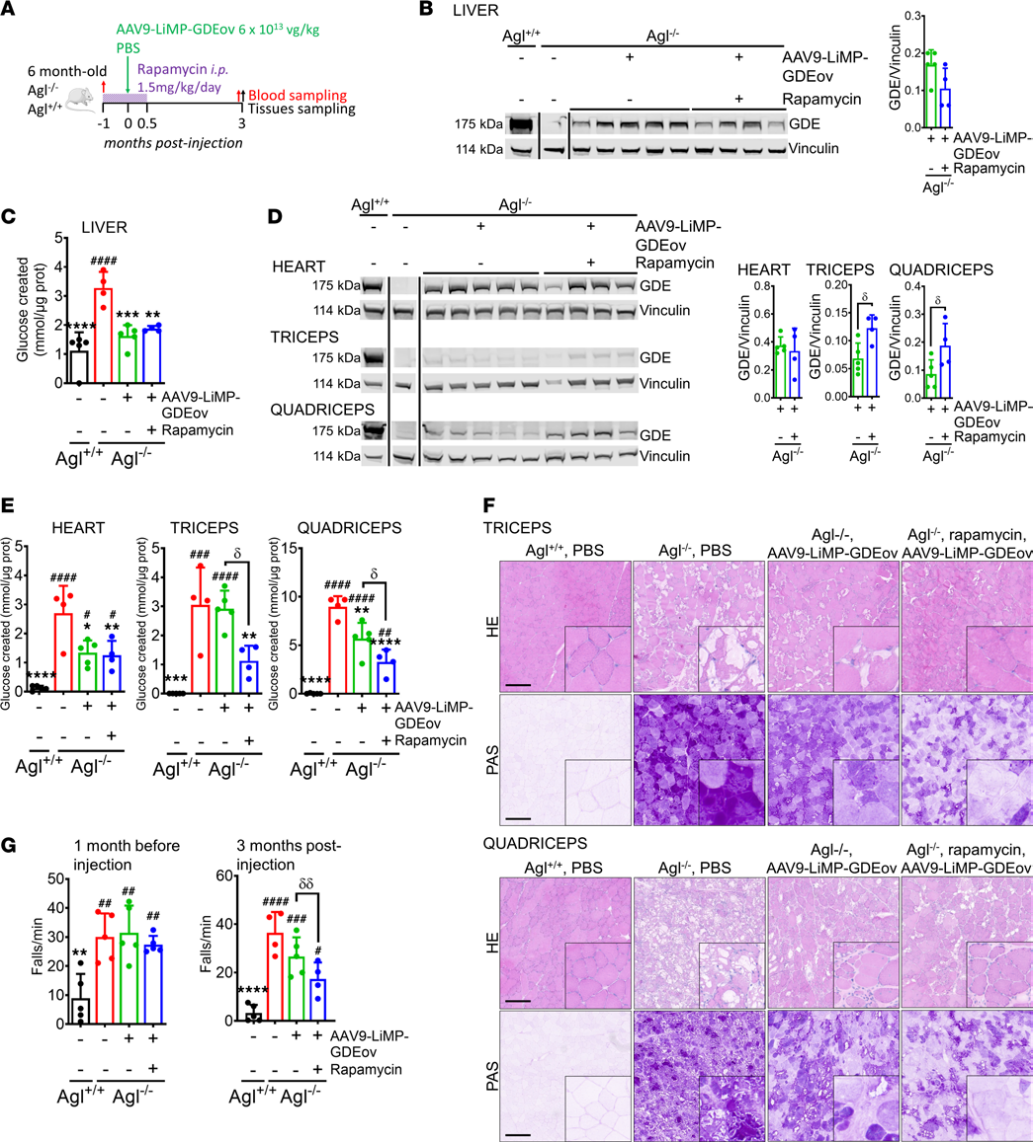
Combination of AAV9-LiMP-GDEov and rapamycin corrects the liver and the muscle impairment in symptomatic *Agl^–/–^* mice. (**A**) Six-month-old *Agl^−/−^* mice received daily i.p. injections of rapamycin at 1.5 mg/kg or vehicle for 6 weeks and were injected with AAV9-LiMP-GDEov at 6 × 10^13^ vg/kg (*n* = 4 per group) 4 weeks after the beginning of the rapamycin treatment. Vehicle-injected *Agl^+/+^* (*n* = 5) and *Agl^−/−^* (*n* = 4) mice received daily injection for 6 weeks. (**B**) Western blot analysis of GDE in liver. The quantification of the GDE protein bands is plotted on the right expressed as ratio to vinculin. (**C**) Glycogen content in liver measured 3 months after vector injection. (**D**) Western blot analysis of GDE in heart, quadriceps, and triceps. The quantification of GDE protein band is plotted on the right expressed as ratio to vinculin. (**E**) Glycogen content in heart, quadriceps, and triceps 3 months after vector injection. (**F**) H&E and Periodic Acid Schiff (PAS) staining performed in triceps and quadriceps. Scale bar: 200 μm. (**G**) Wire hang test shown as number of falls per minute performed 1 month before the beginning of the rapamycin treatment and 3 months after vector injection. Data shown as mean ± SD. Statistical analyses were performed by 1-way ANOVA with Tukey post hoc test. Significance was indicated with * vs. *Agl^−/−^* and ^#^ vs. *Agl^+/+^* or δ (AAV9-LiMP-GDEov vs. Rapamycin + AAV9-LiMP-GDEov treatment groups) as indicated. ^δ^, *, and ^#^*P* < 0.05; ^δδ^, ** and ^##^*P* < 0.01; *** and ^###^*P* < 0.001; and **** and ^####^*P* < 0.0001.

In our experimental conditions, rapamycin treatment alone was not sufficient to clear glycogen in liver and muscle of GSDIII mice. We then tested whether autophagy induction with rapamycin treatment may synergize with the GDE enzyme expression achieved by gene transfer with AAV9-LiMP-GDEov. Symptomatic, 6-month-old *Agl^–/–^* mice were treated daily for 1 month with 1.5 mg/kg/day of rapamycin before the injection of the AAV9-LiMP-GDEov vector, followed by an additional 15 days of the same rapamycin treatment (Figure 3A). Three months after AAV injection, robust transduction levels and GDE expression were detected in liver of *Agl^–/–^* mice treated with AAV9-LiMP-GDEov regardless of the combined treatment (Supplemental Table 1 and Figure 3B). Of note, unlike previous reports (36–38), rapamycin pretreatment did not improve AAV liver transduction in *Agl^–/–^* mice. Consistently, similar correction of glycogen accumulation and histological features was achieved in the liver in the 2 groups (Figure 3C and Supplemental Figure 4A). The lower glycemia values in *Agl^–/–^* mice measured at the start of the rapamycin treatment were corrected 3 months after AAV injection (Supplemental Figure 4B). Despite complete liver glycogen clearance and glycemia rescue, the liver weight was not corrected in AAV9-LiMP-GDEov–treated animals regardless of the use of rapamycin (Supplemental Figure 4C). In the heart, transduction efficacy, GDE expression, partial glycogen clearance, and correction of the histological features were similar between *Agl^–/–^* mice treated with AAV9-LiMP-GDEov with or without rapamycin (Supplemental Table 1, Supplemental Figure 4D, and Figure 3, D and E). Importantly, despite similar transduction (Supplemental Table 1), the combination of rapamycin with AAV9-LiMP-GDEov treatment led to increased GDE expression in quadriceps and in triceps (Figure 3D). Increased GDE expression in skeletal muscle resulted in a better glycogen clearance in the quadriceps and triceps of *Agl^–/–^* mice treated with the combination of rapamycin and AAV9-LiMP-GDEov (Figure 3E). Accordingly, the histological features as measured by H&E and PAS staining were better corrected in quadriceps and triceps of animals treated with the combination of rapamycin and gene therapy than gene therapy alone (Figure 3F). The better glycogen clearance and the normalization of the muscle histology resulted in a better correction of the muscle strength as assessed by wire hang in mice that received the combined treatment (Figure 3G).

Finally, in accordance with what was previously reported, the rapamycin treatment used decreased the level of anti-AAV9 IgG measured all along the protocol. Interestingly, the low levels of anti-GDE IgG measured after AAV vector injection were not affected by the treatment, possibly due to the delayed kinetic of the antitransgene immunity (Supplemental Figure 4E).

### Combination of rapamycin treatment with AAV9-LiMP-GDEov rescues the disease’s molecular signature of the muscle phenotype in GSDIII mice.

To further evaluate the correction of the muscle phenotype at a molecular level, we performed a transcriptomic analysis in triceps of *Agl^–/–^* mice treated with AAV9-LiMP-GDEov with or without rapamycin. We identified a total of 217 differentially expressed genes (DEGs) among the different conditions. Among them, 207 were modified in vehicle-treated *Agl^–/–^* mice and most of them were rescued by the treatment with AAV9-LiMP-GDEov (Supplemental Table 2). To examine gene expression patterns, a heatmap of DEGs was constructed (Supplemental Figure 5A). The findings suggest that the transcriptomic profile of *Agl^–/–^* mice treated with combined gene therapy and rapamycin was closer to PBS-treated *Agl^+/+^* mice than the profile of AAV9-LiMP-GDEov–treated animals (Supplemental Figure 5A), thus supporting the synergic effect observed at both biochemical and functional levels (Figure 3). Validation of 5 upregulated and 4 downregulated genes was performed in triceps and liver, showing a similar pattern of expression than RNA-Seq data (Supplemental Table 3 and 4).

**Figure 4:**
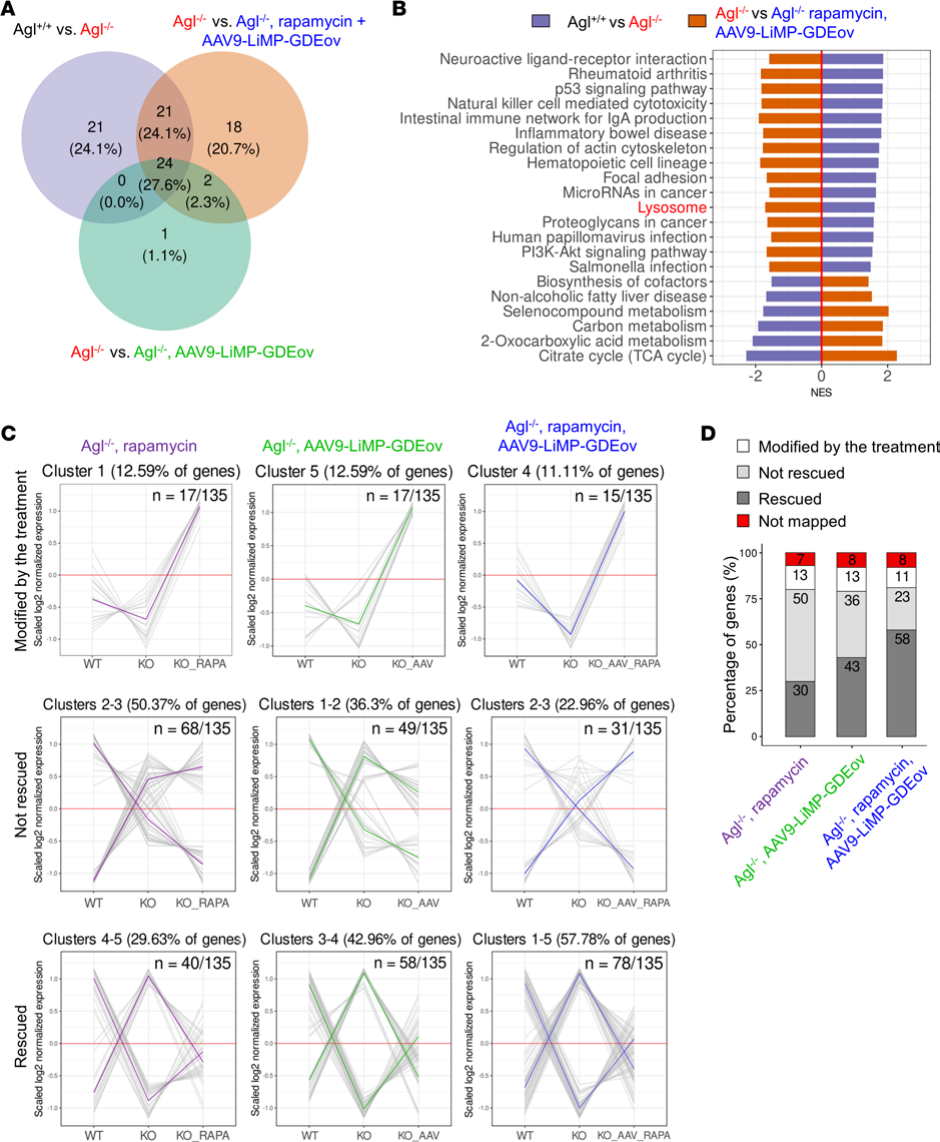
RNA-Seq analysis supports the synergic effect of AAV9-LiMP-GDEov and rapamycin treatment to correct *Agl^–/–^* mice and rescue the lysosomal pathway impairment in triceps. (**A**) Venn diagram representing the overlap of pathways from Gene Set Enrichment Analysis (GSEA) with KEGG database in *Agl^+/+^* *Ag^-^* vs. *Agl^−/−^*, *Agl^−/−^* vs. *Agl^−/−^* AAV9-LiMP-GDEov, and *Agl^−/−^* vs. *Agl^−/−^* AAV9-LiMP-GDEov, rapamycin mice. (**B**) Bar plot representing the 21 common pathways found dysregulated between *Agl^+/+^* vs. *Agl^−/−^* mice and *Agl^−/−^* vs. *Agl^−/−^* AAV9-LiMP-GDEov, rapamycin mice by using GSEA with KEGG database. NES, Normalized Enrichment Score. (**C**) K-means clustering analysis on the lysosomal pathway genes between the 3 treatments: *Agl^−/−^* mice treated with rapamycin alone, AAV9-LiMP-GDEov alone, and the combined treatment. The 3 clusters identified contained genes in which the expression levels were: (a) unaffected by the disease, modified by the treatment; (b) modified by the disease but not rescued by the treatment; and (c) modified by the disease and rescued. The *y* axis represents normalized expression counts, averaged for each condition and scaled for each gene. Gray lines represent the expression profile of individual genes. Colored lines represent average expression profiles for each of the clusters. (**D**) Comparison of the number of genes in the 3 subfamilies (modified by the treatment, not rescued, rescued) among the 3 treatments. Values are expressed in percentage of total.

**Figure 5:**
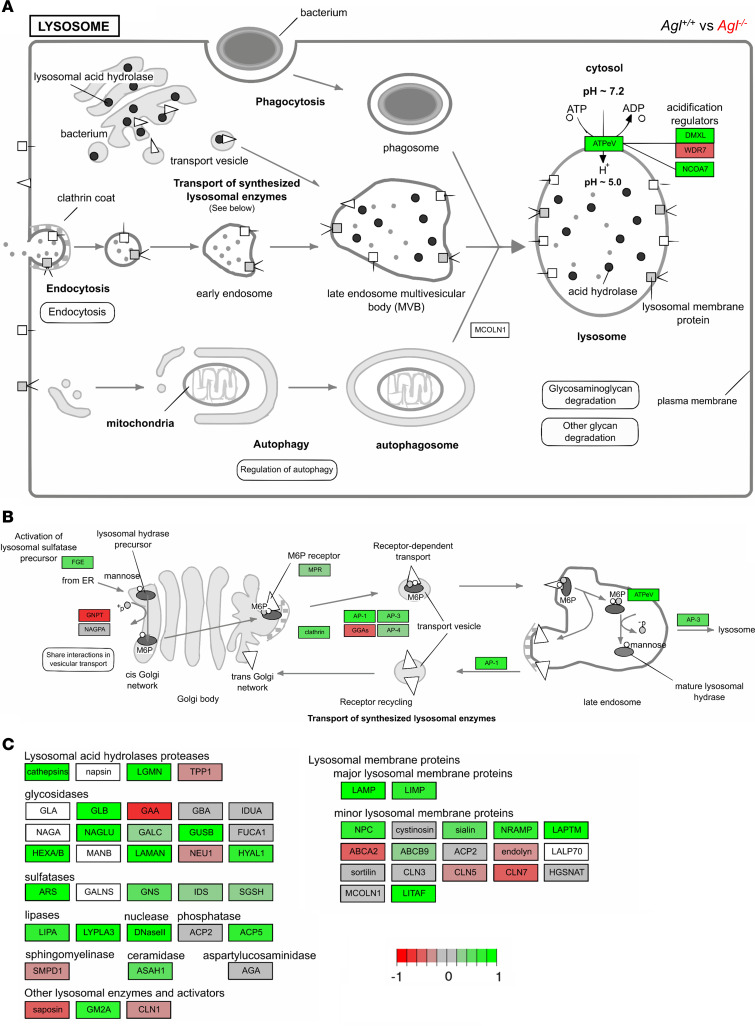
Visualization of the lysosome pathway from KEGG (mmu04102) between *Agl^+/+^* and *Agl^–/–^* mice, in triceps, using Pathview package in R. Proteins and corresponding genes names are indicated in Supplemental Table 6. Fold-change levels of upregulated and downregulated genes are represented in green or red colors, respectively. (**A**) Overview of lysosome formation and autophagy. (**B**) Transport of newly synthetized lysosomal enzymes to the lysosome. (**C**) Main lysosomal proteins and enzymes transcriptomic expression levels.

**Figure 6:**
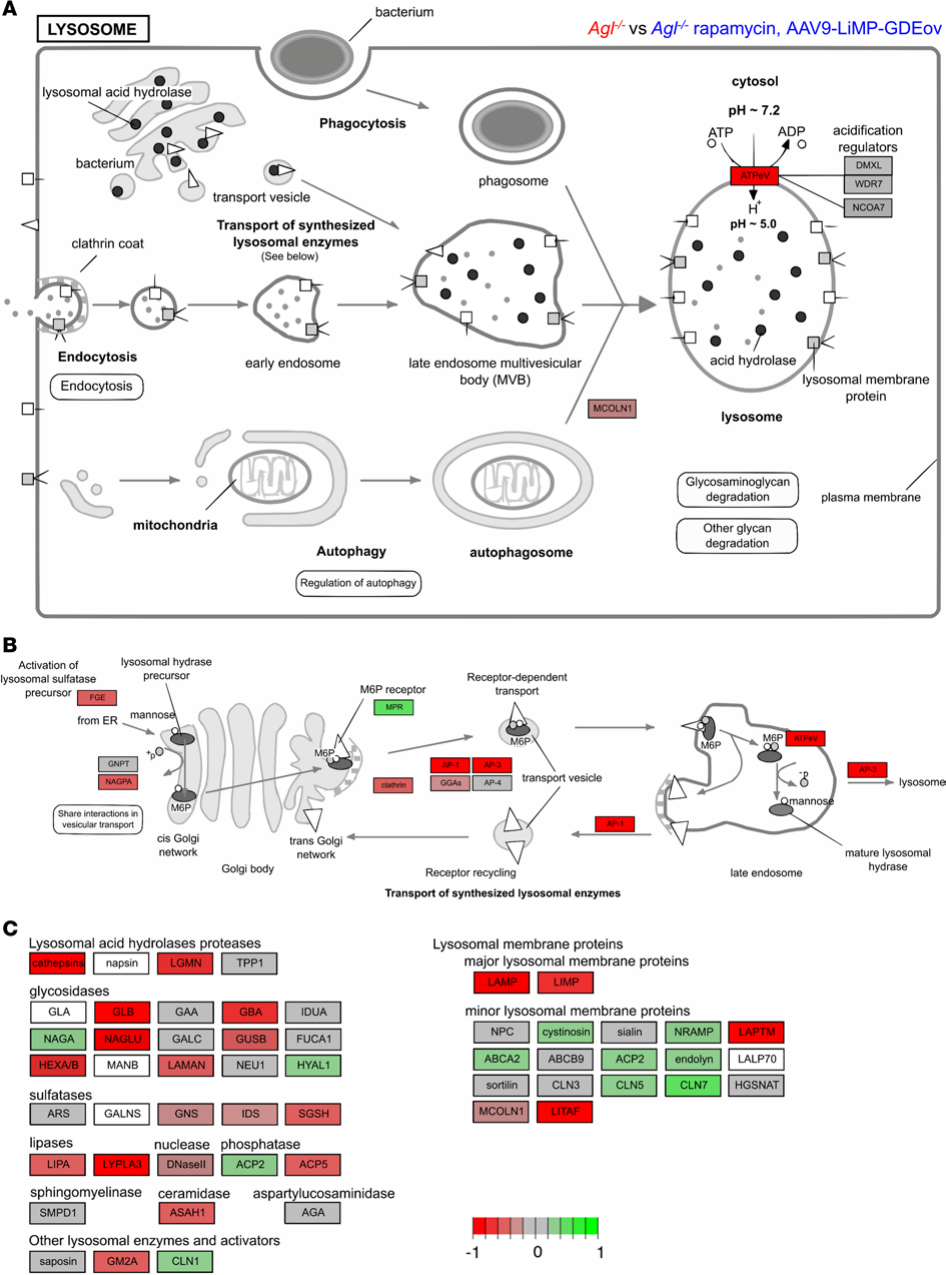
Visualization of the lysosome pathway from KEGG (mmu04102) between *Agl^–/–^* and *Agl^–/–^*, rapamycin, AAV9-LiMP-GDEov mice, in triceps, using Pathview package in R. Proteins and corresponding genes names are indicated in Supplemental Table 6. Fold-change levels of upregulated and downregulated genes are represented in green or red colors, respectively. (**A**) Overview of lysosome formation and autophagy. (**B**) Transport of newly synthetized lysosomal enzymes to the lysosome. (**C**) Main lysosomal proteins and enzymes transcriptomic expression levels.

To further characterize this synergism, we performed a gene set enrichment analysis with the kyoto encyclopedia of genes and genomes set of pathways (GSEA-KEGG) analysis in triceps. We identified 66 pathways markedly dysregulated between untreated *Agl^+/+^* and *Agl^–/–^* animals (Figure 4A). Among these pathways, 24 were rescued in AAV9-LiMP-GDEov–treated animals regardless of the combination with rapamycin (Supplemental Figure 5B). Twenty-one additional pathways were rescued only when the dual AAV vector was combined with the immunomodulatory drug, further supporting the synergic effect of rapamycin and AAV9-LiMP-GDEov (Figure 4, A and B). Importantly, “Lysosome’’ was among the pathways modified only by the combined treatment (Figure 4B). Given the fundamental role of lysosomes in autophagy and the recent report of a autophagy impairment in muscles from patients’ biopsies and GSDIII mice (31), we decided to characterize in detail the effect of the combined treatment on this pathway. To demonstrate the synergic effect of rapamycin and AAV9-LiMP-GDEov, we also included in this analysis a group of *Agl^–/–^* mice treated with rapamycin. K-means clustering of the genes belonging to the “Lysosome’’ pathway as defined in KEGG allowed us to group the genes of this family based on their profile in 3 main groups: (a) modified by the treatment (i.e., those genes similar in *Agl^+/+^* and *Agl^–/–^* mice, unmodified by the disease), (b) modified by the disease and not rescued, and (c) modified by the disease and rescued (Figure 4C). Importantly, mice treated with the combination of AAV9-LiMP-GDEov and rapamycin showed 58% of the gene in the category “modified by the disease, rescued,” while only 23% of the genes were in the category “modified by the disease, not rescued.” A lower percentage of rescued genes was observed in the lysosomal pathway measured in *Agl^–/–^* mice treated with rapamycin alone (30%) or with AAV9-LiMP-GDEov gene therapy alone (43%; Figure 4D and Supplemental Table 5). Visualization of the lysosomal pathway by Pathview showed that most of the genes modified in *Agl^–/–^* mice in this pathway were rescued by the combined treatment, further supporting the synergic effect of the 2 treatments for the rescue of this pathway (Figure 5, Figure 6 and Supplemental Table 6).

To functionally validate the results obtained by RNA-Seq, we explored the pathways of global autophagy. First, we measured acid α-glucosidase (GAA) activity in triceps, which was unchanged in *Agl^–/–^* mice, treated or untreated, compared with *Agl^+/+^* mice (Supplemental Figure 6A), suggesting the absence of a mechanism compensating the increased glycogen accumulation with an enhancement of the lysosomal degradation pathway. In addition, as observed in the previous protocol, p62 protein levels were similar between *Agl^+/+^* and *Agl^–/–^* mice and were not increased by rapamycin treatment after 2.5 months of wash-out of the drug (Supplemental Figure 6B). Interestingly, the P-Gys1/Gys1 protein ratio, elevated in *Agl^–/–^* mice, was rescued only when rapamycin and AAV9-LiMP-GDEov were used in combination (Supplemental Figure 6C), confirming the necessity to combine the expression of GDE and rapamycin to fully rescue the glycogen metabolism in skeletal muscles of *Agl^–/–^* mice. These data support our current working hypothesis that, in skeletal muscle, the transient effect of rapamycin on autophagy induction is beneficial only when combined with the clearance of cytosolic glycogen by reexpression of GDE.

Taken together, the biochemical and functional data and the extended bioinformatic analyses suggest that, while gene therapy treatment alone rescues the metabolic impairment in GSDIII mice, autophagy induction by rapamycin simultaneous to GDE transgene expression by AAV gene therapy both contribute to achieve a significant correction of the muscle phenotype in GSDIII mice.

### AAV9-LiMP-GDEov has an immunological footprint in GSDIII liver reverted by rapamycin treatment.

To evaluate the molecular events underlying the phenotypical correction observed after AAV9-LiMP-GDEov treatment in the liver, we performed an RNA-Seq analysis to compare the transcriptomic changes occurring in AAV-treated *Agl^–/–^* animals.

**Figure 7:**
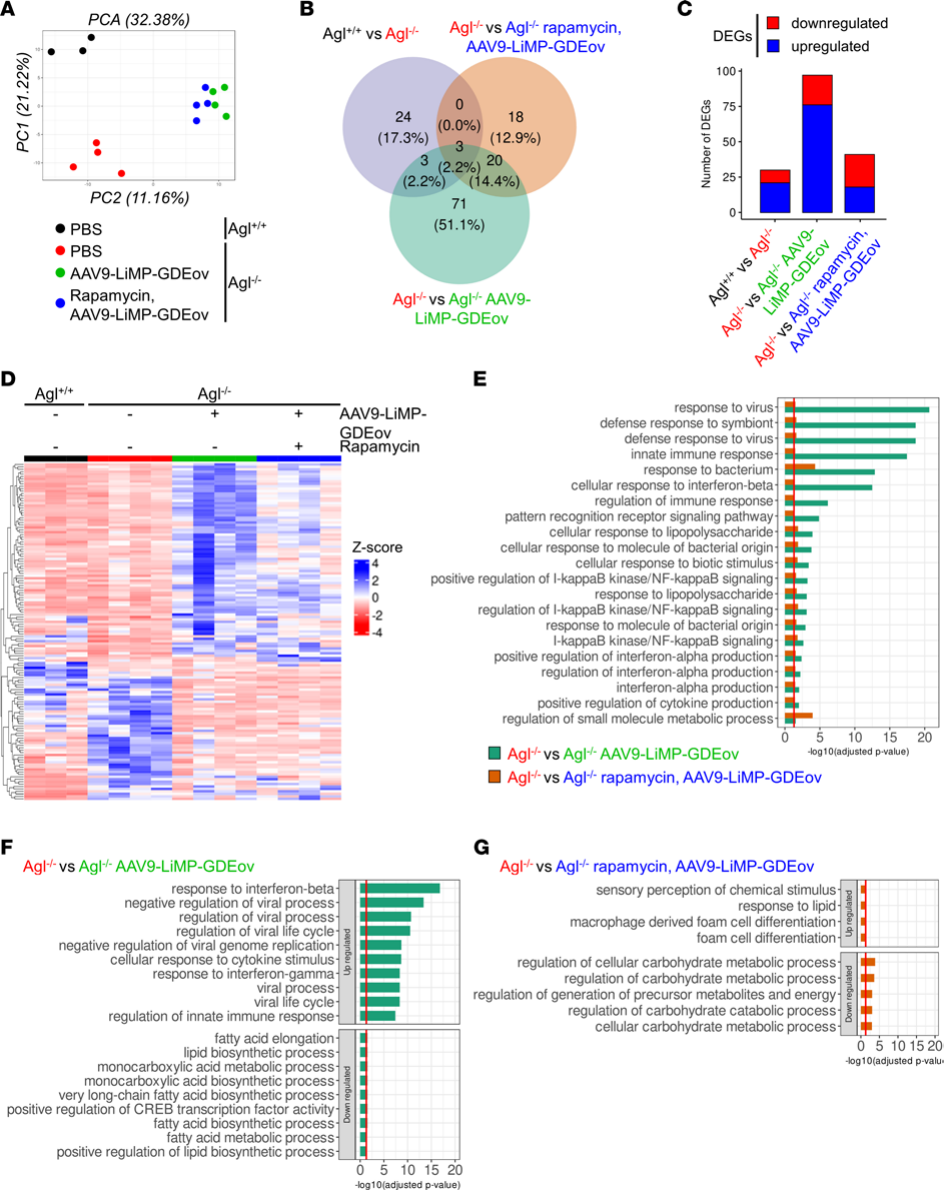
RNA-seq analysis reveals an immunogenic footprint induced by AAV9-LiMP-GDEov injection in *Agl^–/–^* mice partially reverted by rapamycin treatment in liver. (**A**) Principal Component Analysis (PCA) of RNA-Seq data using DESeq2. Samples are colored according to each group. (**B**) Venn diagram representing the overlap of DEGs among the 3 comparisons (*Agl^–/–^* vs. *Agl^+/+^*; *Agl^–/–^* vs. *Agl^–/–^*, AAV9-LiMP-GDEov; *Agl^–/–^* vs. *Agl^–/–^*, rapamycin, AAV9-LiMP-GDEov). (**C**) Number of up- and downregulated genes among differentially expressed genes (DEGs) in each pairwise comparison (*Agl^–/–^* vs. *Agl^+/+^*; *Agl^–/–^* vs. *Agl^–/–^*, AAV9-LiMP-GDEov; *Agl^–/–^* vs. *Agl^–/–^*, AAV9-LiMP-GDEov, rapamycin). (**D**) Heatmap representing the DEGs among all comparisons. Expression levels of upregulated and downregulated genes are represented in blue or red, respectively. (**E**) Bar plot depicting the 21 common enriched GO terms of up- and downregulated DEGs found between *Agl^–/–^* mice and *Agl^–/–^*, AAV9-LiMP-GDEov–treated mice with and without rapamycin treatment. (**F** and **G**) Bar plots showing the top enriched GO terms of up- and downregulated DEGs found between vehicle-treated *Agl^–/–^* mice and *Agl^–/–^* mice receiving the AAV9-LiMP-GDEov treatment alone (**F**) or the combined AAV9-LiMP-GDEov–rapamycin treatment (**G**).

Principal component analysis (PCA) performed on all the genes showed that the largest principal component (PC1) supported a clear separation between PBS-treated *Agl^–/–^* and *Agl^+/+^* mice (Figure 7A). Interestingly, AAV9-LiMP-GDEov–treated *Agl^–/–^* mice, which were equidistant from *Agl^–/–^* and *Agl^+/+^* mice in PC1, were completely separated from the untreated groups in the second principal component (PC2) (Figure 7A).

To further decipher the effect of the AAV vector treatment, differential expression analysis was performed among the 4 groups. In the liver, 139 DEGs were reported among all conditions, with only 30 genes (21 upregulated and 9 downregulated) being dysregulated between *Agl^+/+^* and *Agl^–/–^* mice (Figure 7, B and C, and Supplemental Table 7). Interestingly, the highest number of DEGs, 97 (76 gene upregulated and 21 downregulated), was observed between PBS and AAV9-LiMP-GDEov*–*treated *Agl^–/–^* mice, while more than 2 times fewer DEGs — 41 (18 genes upregulated and 23 downregulated) — were identified in *Agl^–/–^* mice treated with the combination of AAV9-LiMP-GDEov with rapamycin as compared with control animals (Figure 7, B and C, and Supplemental Table 7).

A heatmap was constructed to visualize similarities and differences in gene expression profiles of DEGs (Figure 7D). As predicted by the PCA analysis, AAV9-LiMP-GDEov–treated *Agl^–/–^* mice had a distinctive gene expression pattern, although most of the genes upregulated in *Agl^–/–^* mice treated with AAV9-LiMP-GDEov were not dysregulated when mice received the combination of AAV9-LiMP-GDEov with rapamycin.

Taken together, these data suggest that the expression of GDE by AAV changes the transcriptional profile of *Agl^–/–^* mice without reverting them to *Agl^+/+^* mice. Rapamycin treatment seems to somehow antagonize this effect, leading to a partial normalization of the AAV-treated livers.

To further characterize the specific effect induced by AAV9-LiMP-GDEov in the liver, a Gene Ontology (GO) analysis was then performed on the DEGs identified between untreated *Agl^–/–^* mice and mice of the same genotype treated with AAV9-LiMP-GDEov alone or in combination with rapamycin (Figure 7, E–G, and Supplemental Table 8). Notably, most of the downregulated DEGs in *Agl^–/–^* versus AAV9-LiMP-GDEov–treated mice were enriched in pathways related to fatty acids, carbohydrates, and glycogen metabolism (Figure 7, E and F, and Supplemental Table 8). On the contrary, the upregulated DEGs in *Agl^–/–^* versus AAV9-LiMP-GDEov–treated mice had the highest *P* value and were enriched in pathways such as response to IFN-β, negative regulation of viral process, downregulation of viral life cycle, and regulation of innate immune response (Figure 7, E and F, and Supplemental Table 8). Among the pathways related to immune response, some were also identified in GSDIII mice treated with AAV9-LiMP-GDEov in combination with rapamycin, although with a lower *P* value (Figure 7G and Supplemental Table 8).

To understand whether the response to AAV in GSDIII mice was dependent on the IFN response genes, we performed an extended analysis on a subset of DEGs involved in the IFN response as identified by the interferome database (39, 40). While no differences were observed between *Agl^+/+^* and *Agl^–/–^* mice, we found that 57 of 63 IFN response genes were increased in GSDIII mice treated with the AAV9-LiMP-GDEov vector as compared with PBS-treated *Agl^–/–^* mice (Supplemental Figure 7). Of note, treatment with rapamycin reduced the level of expression of 55 genes (Supplemental Figure 7), thus supporting the involvement of this pathway in the immune footprint of AAV vectors observed in GSDIII mice and counteracted by rapamycin treatment.

Further characterization of the cells infiltrating the liver was performed by immunofluorescence staining of CD8^+^ cells and *CD8* mRNA expression level. No significant differences were observed in the level of infiltrates measured in mice injected with AAV9-LiMP-GDEov vector as compared with PBS-treated control mice, suggesting a limited effect on the infiltrating CD8^+^ T cells 2.5 months after the rapamycin treatment (Supplemental Figure 8, A and B).

These results suggest that, while significant transcriptional changes occurred after treatment with AAV9-LiMP-GDEov in GSDIII livers, at 2.5 months after rapamycin treatment, there was no evident effect on the number of infiltrating CD8^+^ cells. To evaluate any contribution of the GSDIII background on the activation of the immune response, we performed a characterization of the immune cells infiltrating the livers in 6-month-old *Agl^+/+^* and *Agl^–/–^* mice (Supplemental Figure 8C). An increased number of infiltrating immune cells in *Agl^–/–^* mice was observed as measured by counting the total CD45^+^ cells (Supplemental Table 9). While no differences were observed in the number of neutrophils or T lymphocytes populating the livers of GSDIII mice, a significant increase was observed in CD11b^+^F4/80^+^ liver macrophages (Supplemental Table 9). When activation markers were evaluated, both M1 and M2 polarized macrophages (expressing CD80 and CD206, respectively) were found to be increased in *Agl^–/–^* mice. Interestingly, when those numbers were reported as percentages of macrophages, an inverse tendency was observed for the 2 populations, with M1 frequency increased and M2 decreased in *Agl^–/–^* mice as compared with *Agl^+/+^* (Supplemental Figure 8D). Taken together, these data suggest that the GSDIII liver contains a higher number of macrophages with a higher frequency of proinflammatory M1 macrophages.

## Discussion

Despite the first reports by Illingworth et al. more than 60 years ago (41), no curative treatment exists for GSDIII. Among the possible causes of this delay, one of the most important is the multiorgan phenotype of GSDIII, with metabolic impairment and muscle weakness as the 2 main disease manifestations.

Given their capacity to efficiently target several tissues, AAV-base gene transfer appears as an ideal therapeutic option for GSDIII. However, the need to target simultaneously liver and muscle, the high doses needed to treat the muscle, and the size of the transgene represent a large barrier toward the development of an effective gene replacement strategy for this disease.

We previously reported dual overlapping AAV vectors to produce full-length GDE after recombination (22). However, the only dual approach able to correct both liver and muscles at short term was the one using a CAG promoter, and this approach may represent a risk regarding immune responses (13, 27, 28) and tumor development in liver (29).

Because of these challenges, we thought to combine the overlapping strategy to express the full GDE transgene with a tandem promoter previously described for efficient liver and muscle targeting (13) and to evaluate the efficacy of such an approach at long-term. Importantly, while we observed stability in the correction of muscle phenotype, the metabolic impairment was only transiently corrected, possibly because of hepatocytes increased proliferation, a feature of the underlying hepatic pathology in GSDIII (3, 42).

In an attempt to improve AAV gene transfer efficacy, we focused our attention on the combination of the dual vector approach with rapamycin. Rapamycin, an FDA-approved drug, presents several interesting features for GSDIII treatment. First, the use of this drug has been reported to reduce glycogen accumulation and prevent fibrosis in a dog model of GSDIII (26). Second, rapamycin is an autophagy inducer, and autophagy has been reported to be impaired in muscle of patients with GSDIII (31). Finally, rapamycin is widely used in the clinic as an immunosuppressive drug and demonstrated the capacity to delay the immune response after AAV vector administration (32, 43–45) and to increase AAV transduction in the liver, in mouse, and in NHP (36, 37, 46).

Surprisingly, in GSDIII mice, rapamycin treatment alone had no effect on glycogen accumulation, possibly due to differences in the dose of rapamycin used in GSDIII dogs and the duration of the treatment (26). Interestingly, the levels of p62, a protein that accumulates in autophagosomes, already increased in the liver of GSDIII mice were only slightly reduced by rapamycin treatment, suggesting an underlying block of autophagy in this tissue. On the contrary, p62 levels were unchanged in GSDIII muscle, and when autophagy was induced by rapamycin treatment, we observed an accumulation of this autophagosome marker, suggesting an impairment in the clearance of autophagosomes. Although these data may seem counterintuitive, our working hypothesis is that the slower clearance rate of autophagosomes may be due to the glycogen overload of autophagosomes that saturates the GAA activity in GSDIII muscle.

Given this difference in the autophagy activation observed in the 2 tissues, we hypothesized that autophagy induction by rapamycin treatment combined with glycogen clearance by the reexpression of the GDE transgene may synergize in the muscle where the autophagy induction seems limited by the extensive glycogen accumulation.

Differently from what previously reported (36–38), rapamycin treatment in liver did not result in increased transduction, and it seems to induce some degree of toxicity. We consider that this may be due to differences in the drug used — i.e., encapsulated vs. free rapamycin, the time of treatment and the doses used. However, one intriguing hypothesis may be that the difference observed might be due to the abnormal autophagic flux observed in GSDIII mice and the absence of autophagy induction.

AAV9-LiMP-GDEov treatment either alone or in combination with rapamycin was able to rescue the metabolic impairment and glycogen accumulation in liver and heart. Importantly, GSDIII mice treated with the combination of AAV9-LiMP-GDEov and rapamycin showed better rescue of glycogen accumulation and muscle strength than those treated with the vector alone, suggesting that the hypothesized synergism was indeed present, at least in this tissue. The synergic effect was also evident at the molecular level when an RNA-Seq analysis was performed in triceps. In particular, the lysosomal pathway was impaired in the muscle of untreated GSDIII mice, thus providing a potential rationale on the lack of efficacy when autophagy was induced by rapamycin. It is thus conceivable to hypothesize that the cytosolic glycogen clearance achieved by gene replacement with GDE facilitates autophagy induction by rapamycin, ultimately leading to a complete normalization of the lysosomal pathway and a full restoration of the glycogen degradation pathway in muscle fibers.

To evaluate whether rapamycin treatment had a similar effect on the liver, we performed a transcriptomic analysis in liver. Surprisingly, we identified a clear gene expression pattern in *Agl^–/–^* mice treated with the vector. Functional analysis of the genes modified by the AAV9-LiMP-GDEov treatment revealed that almost all of them were IFN-responsive genes. Immunosuppressive rapamycin treatment performed at the time of the injection was able to decrease the activation of these genes, thus suggesting a role of the immune system in the early responses to AAV vectors in the liver of GSDIII mice.

These findings indicate that, in GSDIII mice, expression of the GDE transgene results in the formation of low-level anti-GDE antibodies as well as in a tendency toward increased infiltration of CD8^+^ cells in the liver. The presence of CD8^+^ cells in the liver could be related to the establishment of a tolerance status toward the transgene (47, 48) that was not rejected in muscle in the long-term experiment presented in this manuscript. One captivating finding of this manuscript is the reported presence of a larger population of macrophages M1 and M2 in the GSDIII liver at the time of vector injection. M1 macrophages play a proinflammatory role, while M2 macrophages are generally antiinflammatory, promote immunomodulation, and are involved in tissue reparation. When the frequency of these macrophage populations was measured relative to the total infiltrating macrophages, we found a tendential increase in M1 and a decrease in M2 macrophage populations suggestive of a proinflammatory environment in the GSDIII liver. Interestingly, rapamycin treatment has been reported to polarize macrophage differentiation toward an M2 phenotype (49). Here, we hypothesize that the reduction of the immune footprint of the AAV vector measured 2.5 months after the wash-out of rapamycin may be due to an M2 polarization promoted by the drug that resulted in a better control of the immune response toward the AAV vector. Further evaluation of the determinants of the immune response in GSDIII mice and, in particular, of the role of nonparenchymal cell profile in the formation of the immune response and the persistence of the GDE transgene is currently ongoing.

One limitation of our study is the use of only 1 dose, 1 duration, and 1 route of administration of rapamycin. Testing multiple doses of rapamycin, in particular, could be of interest in this disease, as rapamycin effect is known to be different according to the dose (50). Further investigations of the detailed mechanism behind this immune response to the vector are ongoing, although we consider the prefibrotic status of the liver as an important component of this response.

One important conclusion of this study is that the combination of rapamycin and gene therapy could reduce the dose of AAV required for the treatment of GSDIII. However, in our study, the total dose of AAV used is about 6 × 10^13^ vg/kg (3 × 10^13^ vg/kg of each AAV), not far from doses that showed toxicity in the clinic (51, 52). To circumvent the limitation imposed by the use of high doses of AAV vectors, novel approaches based on the use of single AAV vectors expressing a truncated GDE combined with AAV vectors with improved muscle targeting are being evaluated in glycogenosis (53, 54). Regardless, this manuscript provides support to the use of rapamycin in combination with gene therapy and identifies a potential mechanism behind the observed synergism between the small molecule and AAV gene transfer. In the next future, the combination of small molecules with improved AAV gene therapy approaches may increase safety while reducing the effective dose.

Future clinical development of the combination treatment may require optimization of the approach by testing several doses of rapamycin and protocol designs as well as AAV vectors to express GDE in both tissues.

To conclude, these results suggest that GSDIII liver is prone to the development of immune responses to AAV vectors that can be controlled by a transient immunosuppressive treatment and support the development of combination approaches for the treatment of diseases with underlying liver degeneration.

## Methods

### Sex as a biological variable

Only males were used in these studies, based on previous reports that AAV liver transduction is less efficient in females than in males (55).

### Clonings and AAV vector production

LiMP promoter was already described (13). Briefly, it consists of the association of the human α-1 anti-trypsin and the synthetic spC5-12 promoters. The design of the overlapping vectors was already described (22). The “Head” vector containing the first part of the final sequence is 4.4 kb, and the “Tail” vector containing the end of the final sequence is 4.1 kb. The 2 overlapping expression cassettes were flanked by the inverted terminal repeats of AAV serotype 2 for vector packaging. Transgene expression cassettes of similar size are fully encapsidated with percentages of full capsids ranging from 30% to 70% depending on the composition.

AAV vectors were produced by an adenovirus-free transient transfection method (56) and purified as described earlier (57). AAV vector titers were determined by quantitative PCR (qPCR) as already described (22).

### Mouse model

All studies were conducted in *Agl-*KO mice (*Agl*^–/–^) and WT (*Agl^+/+^*) littermates. Mice generation and phenotype were reported in previous work (22). Briefly, mice were generated in a pure C57BL/6J background by replacing exons 6–10 of the *Agl* gene with a neomycin-expressing cassette, and they were then bred into a mixed BALB/c background.

### In vivo studies in mice

In all mouse studies, animals were randomly assigned to treatment groups. To minimize potential bias during functional assessments in mice, operators were blinded to the study design. Operators in charge of sample analysis were also blinded to study design.

#### Twelve-month follow-up after AAV injection.

Three-month-old *Agl*^–/–^ mice were injected i.v. with 6 × 10^13^ vg/kg (3 × 10^13^ vg of the Head vector, and 3 × 10^13^ vg of the Tail vector) of AAV9-LiMP-GDE overlapping vector (*n* = 4) or PBS in the control, *Agl^+/+^* (*n* = 5), and *Agl*^–/–^ (*n* = 4) groups.

#### One-month daily treatment with rapamycin.

Rapamycin was injected i.p. to *Agl^+/+^* (*n* = 5) and *Agl^–/–^* (*n* = 6) at a dose of 1.5 mg/kg every day for 1 month. Control mice, *Agl^+/+^* (*n* = 5) and *Agl^–/–^* (*n* = 6), were injected with vehicle (NaCl) following the same scheme.

#### Combination treatment.

Six-month-old *Agl^+/+^* and *Agl^–/–^* mice were injected daily i.p. with 1.5 mg/kg rapamycin or vehicle (4 groups: *Agl^+/+^*, rapamycin [*n* = 5]; *Agl^+/+^*, vehicle [*n* = 5]; *Agl^–/–^*, rapamycin [*n* = 7]; and *Agl^–/–^* vehicle [*n* = 9]). After a month, half of the *Agl^–/–^* mice treated with vehicle were injected i.v. with 6 × 10^13^ vg/kg of AAV9-LiMP-GDE overlapping vector (*n* = 5) or PBS (*n* = 4), and half of the *Agl^–/–^* mice treated with rapamycin were injected i.v. with 6 × 10^13^ vg/kg of AAV9-LiMP-GDE overlapping vector (*n* = 4) or PBS (*n* = 3). Rapamycin daily injections were pursued for 15 additional days in these 2 last groups as well as in the *Agl^+/+^*, rapamycin group. Mice were euthanized 3 months after vector injection (or 2.5 months after the end of the rapamycin treatment) at the age of 9 months.

### Wire hang test

Forelimb wire hang test was performed as already reported (22). A 4 mm–thick wire was used to record the number of falls over a period of 3 minutes. The average number of falls per minute was reported for each animal.

### Blood collection, glycemia measures

Normally fed mice were anesthetized for glycemia measurement and blood collection. Blood glucose was measured with an Accu-Check Go glucometer (Roche Diagnostic) using a drop of blood from retro-orbital sinuses. Then, blood samples were collected by retro-orbital sampling into heparinized capillary tubes and mixed with 3.8% sodium citrate, followed by plasma isolation.

### Anti-capsids and anti-transgene responses measurement

First, mice sera were decomplemented by incubating them 30 minutes at 56°C. Then, the concentration of IgM and IgG antibodies was determined by ELISA, as previously described for anti–capsid IgM and IgG (18, 58) and as follows for anti-GDE IgG: 96-well plates were coated with GDE protein (OriGene) at 1 μg/mL and incubated overnight at 4°C; wells were saturated with PBS containing 6% w/v of nonfat milk powder for 2 hours at room temperature and were then incubated with decomplemented sera for 1 hour at 37°C. A biological known positive control, consisting of the decomplemented sera of Sprague-Dawley rats immunized through intramuscular injection of an AAV1 dual vector expressing GDE, was included. A commercial polyclonal rabbit anti-hGDE (16582-1-AP, ProteinTech) diluted at 1:1,000 was used as an internal control for coating. Secondary sheep anti–mouse IgG-HRP (GE Healthcare) and goat anti–rat IgG-HRP antibodies (Thermo Fisher Scientific) were diluted at 1:5,000, while secondary goat anti–rabbit IgG-HRP antibody (Agilent DAKO) was diluted at 1:2,000 before being incubated 1 hour at 37°C. 3, 3′, 5, 5′-tétraméthylbenzidine substrate (TMB) (BD Biosciences) was incubated 15 minutes at room temperature for revelation; then, the reaction was stopped using H_2_SO_4_ 2N before reading the absorbance at 450 nm and 570 nm (for background subtraction) on an EnSpire α plate reader (PerkinElmer).

### Western blot analysis

Snap-frozen tissues were homogenized in UltraPure DNase/RNase-free water (Thermo Fisher Scientific) in the first study or in RIPA buffer (R0278, Sigma-Aldrich) mixed with Complete protease-inhibtor cocktail (11697498001, Sigma-Aldrich) in the second and the third studies, with FastPrep lysis tubes (MP Biomedicals), followed by centrifugation 20 minutes at 10,000*g* at 4°C to collect the supernatant. Protein content in lysates was quantified by BCA Protein Assay (Thermo Fisher Scientific). SDS-page electrophoresis was performed with NuPAGE 4%–12 % Bis-Tris protein gels (Invitrogen). After transfer, the membrane was blocked with Odyssey buffer (LI-COR Biosciences) and incubated with an anti-hGDE antibody (rabbit polyclonal, AS09-454, Agrisera, in liver or rabbit polyclonal, 16582-1-AP, Proteintech, in muscles), anti-p62/SQSTM1 (ab56416, Abcam), anti–phosphorylated GYS (3891, Cell Signaling Technology), anti-GYS1 (3886, Cell Signaling Technology), anti-vinculin (mouse monoclonal, clone V9131, Sigma-Aldrich), or anti-actin antibody (rabbit monoclonal, sc-8432, Santa Cruz Biotechnology). Membranes were then washed and incubated with the appropriate secondary antibody (LI-COR Biosciences) and visualized with the Odyssey imaging system (LI-COR Biosciences). Densitometry analysis was conducted using Image Studio Lite (LI-COR Biosciences) version 5.2. Protein level was normalized to the housekeeping protein (vinculin or actin) and then to the *Agl^+/+^* mice.

### Measurement of glycogen content

Glycogen content was measured indirectly in tissue homogenates as previously described (22).

### Measurement of acid α-glucosidase activity

GAA activity was measured in tissue homogenates as previously described (18).

### Vector genome copy number

Vector genome copies in mice were determined by qPCR on tissue DNA. Total DNA was extracted from tissues homogenates using the DNA & RNA isolation Kit from pathogen (Macherey-Nagel) and the KingFisher apparatus (Thermo Fisher Scientific) extraction method according to manufacturer’s instructions. The primers used in the reaction were located in the human α-1 anti-trypsin promoter region (forward primer 5′-GGCGGGCGACTCAGATC-3′, reverse primer 5′-GGGAGGCTGCTGGTGAATATT-3′), for the vector. The number of vector copies was normalized by the copies of the *titin* gene, which was used as an internal control for each sample (forward primer: 5′-AAAACGAGCAGTGACGTGAGC-3′, reverse primer: 5′-TTCAGTCATGCTGCTAGCGC-3′). Data were expressed as vector genome copies per diploid genome.

### Histology and stainings

Immediately after euthanasia, muscles and a piece of liver were snap-frozen in isopentane (–160°C) previously chilled in liquid nitrogen, and a piece of fresh liver was fixed in formaldehyde and embedded in paraffin. Serial cross-sections (8 mm for muscles and 4 mm for liver) were cut in a Leica CM3050 S cryostat (Leica Biosystems). To minimize sampling error, 2 or 3 sections of each specimen were obtained and stained with H&E or with PAS, and Sirius red for liver, according to standard procedures. Images were acquired using an Axioscan slide scanner (Zeiss), using a plan-apochromat ×10 magnitude 0.45 NA objective. Tile scan images were reconstructed with ZEN software (Zeiss). Anti-CD8 immunostaining was performed according to standard procedures on frozen serial liver slices at a dilution of 1:40, using a monoclonal anti-CD8α antibody (monoclonal rat antibody, clone CT-CD8a, Invitrogen, MA5-17594). For CD8^+^ cells quantification, images were processed using QuPath 0.4.3 Software. A first pixel classifier was trained on different types of liver slices for detecting tissue and eliminating artefacts such as folding, bubbles, and tearings. This contour pixel classifier is a Random Tree with a resolution of 5.2 μm/pixel, and it includes 2 channels and 3 scales of smoothing: 2, 4, and 8; 5 features (Gaussian, Laplacian of Gaussian, Weighted Deviation, Gradient Magnitude, and Structure Tensor Max Eigenvalues); and no local normalization. The quantification of CD8^+^ cells was performed using the included Cell Detection feature on the fluorescence channel of the CD8 staining, using the following parameters: pixel size 1 μm, background radius 10 μM, median filter radius 1.5 μm, Sigma 2 μm, and minimum and maximum area, respectively, 30 and 400μm^2^. The threshold was set to 1,600, and the cell expansion set to 0 μm. The resulting detections are the CD8^+^ cells.

The output parameter is the ratio of the number of CD8^+^ cells over the total surface area of the tissue slice, expressed as cells per millimeter square. The quantification of CD8^+^ cells was performed using a pixel classifier trained on healthy tissue and impaired tissue. The output parameter is the ratio of the number of CD8^+^ cells over the total number of cells on the total tissue slice (quantified by DAPI^+^ staining).

### RNA extraction

Tissues were lysed with FastPrep lysis tubes (MP Biomedicals) using Qiazol (Qiagen) and chloroform (Sigma-Aldrich) reagents. RNA was isolated using RNeasy Plus Mini extraction kit (Qiagen) according to the manufacturer’s instructions. DNase I digestion (Invitrogen) was performed to degrade DNA in the sample. Quantification and quality control of RNA samples were determined using Agilent RNA ScreenTape System (5067-5576, Agilent) following manufacturer’s instructions.

### RNA-Seq and transcriptomic analysis

For each sample, 100 ng of total RNA was used to perform the QuantSeq 3′ mRNA-Seq Library Prep FWD for Illumina (Lexogene) resulting in NGS libraries, which originate from the 3′ end of polyadenylated RNA. Briefly, library generation was started by oligo (dT) priming with primers. After first-strand synthesis, the RNA was removed before the second-strand synthesis was initiated by random primers. Libraries were PCR amplified and barcoded in 17 cycles and were quantified using Agilent High Sensitivity DNA kit (Agilent). In total, 2 nM of pooled libraries were denatured and 1.8 pM was used for cluster generation before single-end sequencing on an Illumina Nextseq550 (Illumina, High output 2 × 75 pb run). Samples were sequenced with an average of 13,232,481 reads per sample. The quality control of the sequencing data was evaluated using FastQC (v0.11.9). The reads were trimmed using Prinseq-lite v0.20.4 (59) (--trim-right 20) and filtered by average quality score (--trim-qual 20) and cutadapt v1.18. Reads were mapped on EnsEMBL GRCm38.99 mouse reference using rna-STAR v2.7.6a (60). Reads below a mapping score of 10 or multimapped were filtered using samtools v0.1.18 (61). The gene expression level in each sample was quantified with HTSeq-count v0.12.4 (62). The differential gene expression (DEG) between conditions was calculated with DESeq2 v1.34.0 (63) using R v4.1.2). We consider that genes are differentially expressed when their BaseMean is greater than 20, their Benjamini-Hochberg adjusted *P* value is less than 0.05 and |log_2_FoldChange| is above 0.4 (options: lfcThreshold = 0.4, altHypothesis = ‘greaterAbs’). Data are available on the GEO database (GSE232166), and programming codes for DEG analysis are available on GitHub (https://github.com/I-Stem-CECS/NGS79; commit ID: 8f7245241b62cc79338956ac1a9040b22ba65eee). The heatmap of the DEGs among all pairwise comparisons of untreated *Agl^+/+^* and *Agl^–/–^*, and *Agl^–/–^* mice treated with AAV9-LiMP-GDEov alone or AAV9-LiMP-GDEov combined with rapamycin was performed using the ComplexHeatmap package (63). Functional annotation analysis of DEGs and GSEA (64) were performed using cluster Profiler package (65) in R. GO terms (66) and KEGG pathways (67) were retrieved from the analysis. Enrichments were considered significant if associated to an adjusted *P* < 0.05. K-means clustering analysis was performed on a set of genes from the lysosome pathway in KEGG. The 5 clusters identified contained genes with similar expression levels. These clusters have been discriminated into 3 sub-groups: (a) unaffected by the disease, modified by the treatment; (b) modified by the disease, not rescued; and (c) modified by the disease, rescued.

### Reverse transcription and qPCR

In total, 2 μg of RNA were subjected to the “DNA-free kit” (Invitrogen) and to reverse transcription using the “RevertAid First Strand cDNA Synthesis Kit” (Thermo Fisher Scientific). The cDNA obtained were quantified using TaqMan mixes for CD8 (Mm01182107_g1) and RPLP0 as housekeeping gene (Mm00725448-s1) (Thermo Fisher Scientific). Data were expressed as –ΔΔCt, which are the Ct normalized with the housekeeping gene values and normalized to Agl^+/+^ group values.

### Flow cytometry

Mouse livers were dissociated onto a 70 μm nylon mesh cell strainer in ice cold phosphate buffer saline (PBS) buffer, and hepatocytes were eliminated by centrifugation at 30*g* for 3 minutes. The supernatant was then centrifuged at 300*g* for 5 minutes, and the resulting cell pellet was resuspended in Percoll to undergo a leukocyte separation step on a Percoll gradient. After centrifugation at 1,300*g* for 20 minutes without acceleration or brake at room temperature, the leukocyte ring was harvested and washed in buffer solution (PBS, 2 mM EDTA, 0.1% human serum albumin [HSA]). Immunostaining for flow cytometry was performed using standard protocols where nonspecific binding of immunoglobulin to Fc receptors is blocked with anti–mouse FcγRIII/II (clone 2.4G2). Cells were then incubated for 30 minutes in PBS labeling buffer, 2 mM EDTA, 0.1% HSA with LIVE/DEAD Fixable near IR Dead Cell Stain (Invitrogen), and antibodies of interest (CD3-Alexa Fluor 488, eBioscience 53-0031-82; CD11b-Percp-efluor710, BioLegend 101227; CD80-PE, BioLegend 104707; CD8-PE-Cy7, BD Pharmingen 552877; CD206-APC, eBioscience 17-2061-82; Gr1–efluor 450, eBioscience 56-5941-82; F4/80–Super bright 780, eBioscience 78-4801-82; CD45–Brilliant ultra violet 395, BD Pharmingen 564279; CD4–Brilliant ultra violet 496, eBioscience 364-0042-82). Cells were fixed with 4% PFA for 15 minutes. Experimental data were acquired on a CytoFLEX LX flow cytometer (Beckman Coulter). All labeling was carried out at 4°C. VersaComp Antibody Capture Bead Kit (Beckman Coulter) compensation beads were used to perform the compensations. Obtained data were processed with FlowJo v10.7.2 software (Becton, Dickinson and Company).

### Statistics

All data shown in the present manuscript are expressed as mean ± SD. GraphPad Prism 7.0 software (GraphPad Software) was used for statistical analyses. *P* < 0.05 was considered significant. Parametric tests were used for data having a normal distribution with α = 0.05. One-way ANOVA with Tukey post hoc correction was used for comparisons of 1 variable between more than 2 groups. All statistical tests were performed 2-sided. The statistical analysis performed for each data set is indicated in the figure legends.

### Study approval

In vivo studies were performed in compliance with all relevant ethical regulations for animal testing and research. Notably, they were performed according to the French and European legislation on animal care and experimentation (2010/63/EU) and approved by Genethon’s ethical committee.

### Data availability

All data presented in the manuscript are either shown in histograms as individual points and/or reported in tables and reported in a separate Supporting Data Values file. The RNA-Seq data are available as a Gene Expression Omnibus (GEO) data set (GSE232166).

## Supplementary Material

Supplemental data

Unedited blot and gel images

Supporting data values

## Version 1 05/16/2024

In-Press Preview

## Version 2 06/10/2024

Electronic publication
