# Supplementary material for: Synergism of dual AAV gene therapy and rapamycin rescues GSDIII phenotype in muscle and liver
Source: JCI Insight. 2024 May 16;9(11):e172614. doi: 10.1172/jci.insight.172614 (PMC11382881; doi:10.1172/jci.insight.172614)
Supplement: Supplemental data [file jciinsight-9-172614-s153.pdf]

SUPPLEMENTARY FIGURES AND TABLES

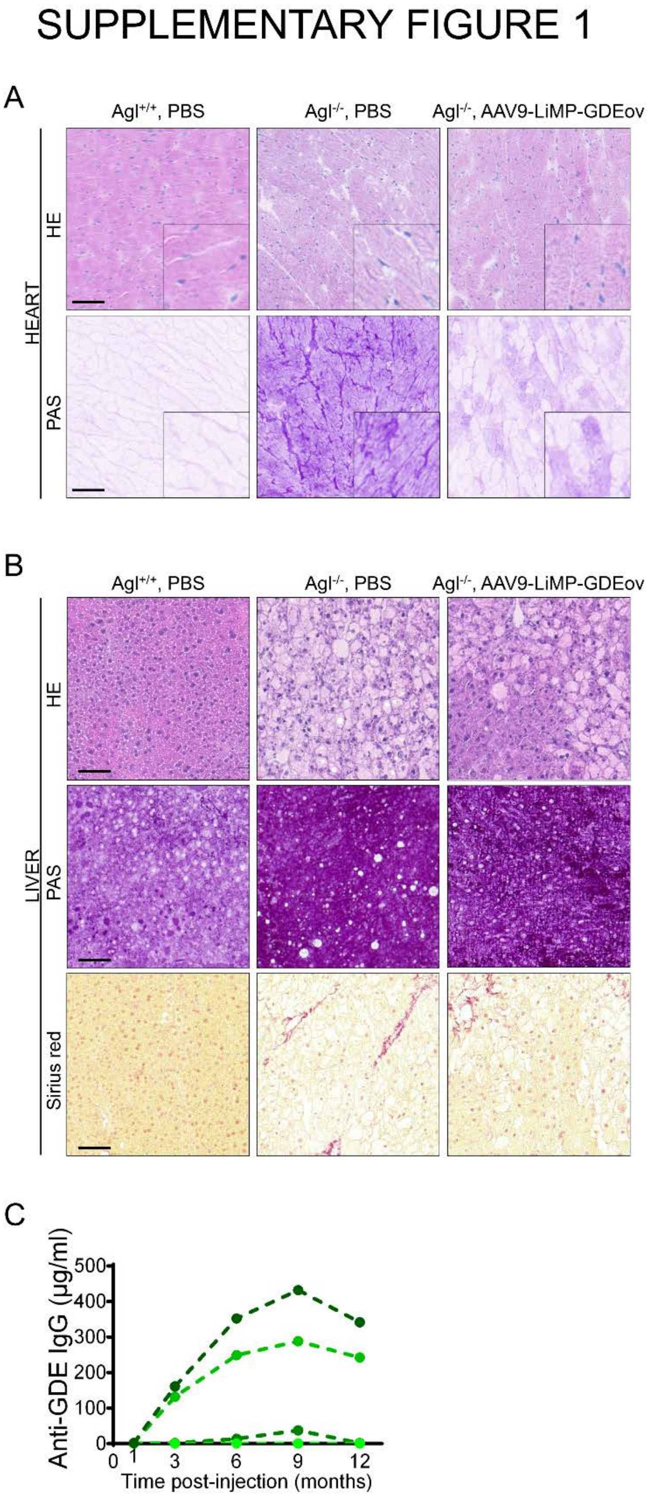

Supplementary Figure 1. 3-month-old *Agl*$^{-/-}$ mice received a single injection of AAV9-LiMP-GDEov at 6 x 10^{13} vg/kg (n = 4 per group) and followed-up for 12 months. PBS-injected *Agl*$^{+/+}$ (n = 5) and *Agl*$^{-/-}$ (n = 4) mice were used as controls. A. Hematoxylin and Eosin (HE) and Periodic Acid Schiff (PAS) staining performed in heart; scale bar 100 µm. B. Hematoxylin and

Eosin (HE) and Periodic Acid Schiff (PAS) and Sirius red (SR) in liver sections; scale bar 100 µm. C. Anti-transgene antibody titers overtime, each curve represents a single animal.

SUPPLEMENTARY FIGURE 2

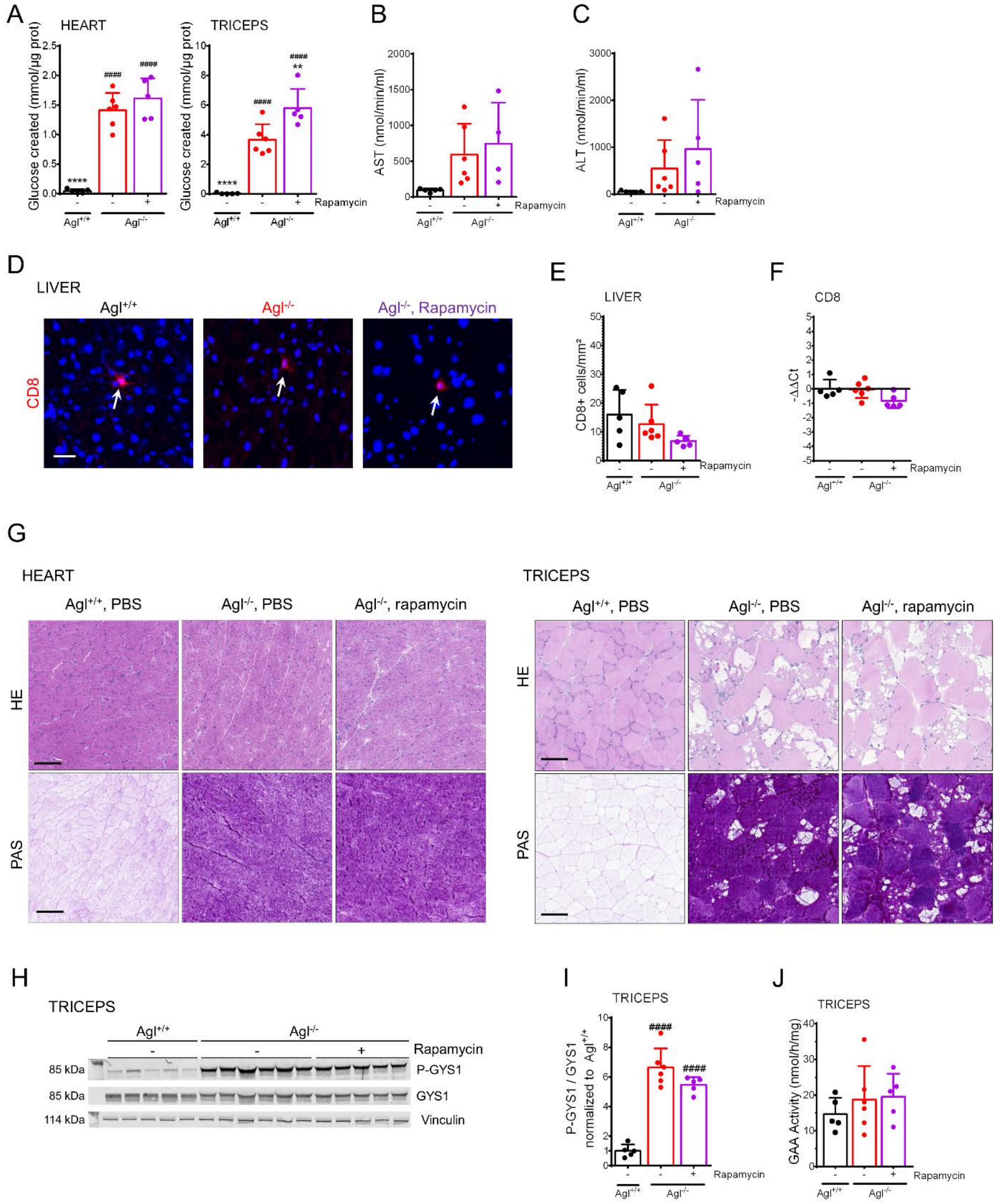

Supplementary Figure 2. 6-month-old *Agl*$^{-/-}$ mice received daily intraperitoneal injections of rapamycin at 1.5 mg/kg for a month (n = 5 per group). Vehicle-injected *Agl*$^{+/+}$ (n = 5) and *Agl*$^{-/-}$ (n = 5) mice were used as controls. A. Glycogen content measured in heart and triceps at the end of the treatment. B-C. Liver transaminases, (B) aspartate aminotransferase (AST) and (C) alanine aminotransferase (ALT) measured in serum at the end of treatment D-E.

Immunofluorescent staining of nucleus (DAPI) and CD8 positive cells (red) in liver at the end of the treatment (D) (scale bar 20 µm), and quantification (E) of CD8 positive cells in liver section. White arrows indicate a cell with DAPI positive and CD8 positive staining. F. Transcriptomic levels of expression of CD8 in liver. Data is represented as -ΔΔCt. G. Hematoxylin and Eosin (HE) and Periodic Acid Schiff (PAS) staining performed in heart, and triceps; scale bar 100 µm. H-I. Western blot analysis of phosphorylated GYS (P-GYS) and GYS in triceps (H) and quantification (I) expressed as ratio to P-GYS/GYS. J. Acid alpha-glucosidase (GAA) activity in triceps at the end of the treatment. Data shown as mean ± SD. Statistical analyses were performed by one-way ANOVA with Tuckey post-hoc test. Significance indicated with * vs. *Agl*$^{-/-}$ and $^{\#}$ vs. *Agl*$^{+/+}$. ** $p<0.01$, **** and $^{\#\#\#\#}$ $p<0.0001$.

SUPPLEMENTARY FIGURE 3

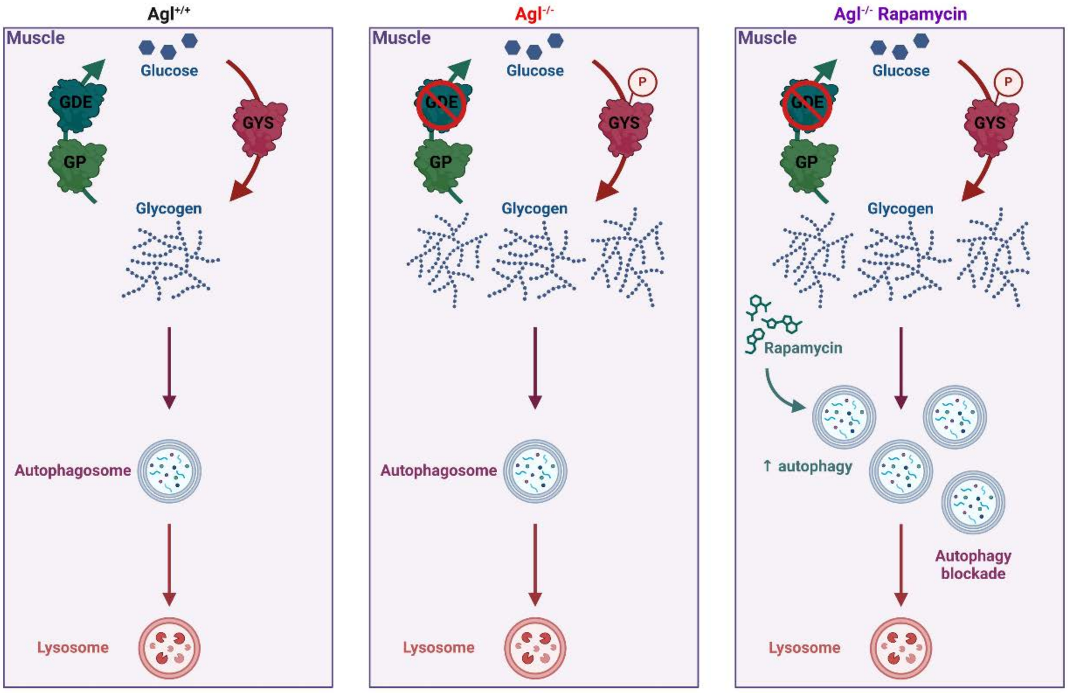

Supplementary Figure 3. Proposed model of autophagic flux, related to glycogen content, in GSDIII skeletal muscles. In Agl$^{+/+}$ mice, glycogen is synthesized and degraded (by the cytosolic pathway or autophagy of glycogen namely glycophagy) according to the energy needs. In Agl$^{-/-}$, the lack of GDE leads to an accumulation of cytosolic glycogen and to glycogen synthase (GYS) inhibition. Glycogen degradation in the lysosomal compartment remains unchanged. After a month of daily rapamycin, autophagy induction leads to increased glycophagy. However, the unchanged capacity to degrade glycogen in the lysosomal compartment lead to unpaired autophagosomes clearance, and autophagy blockade.

SUPPLEMENTARY FIGURE 4

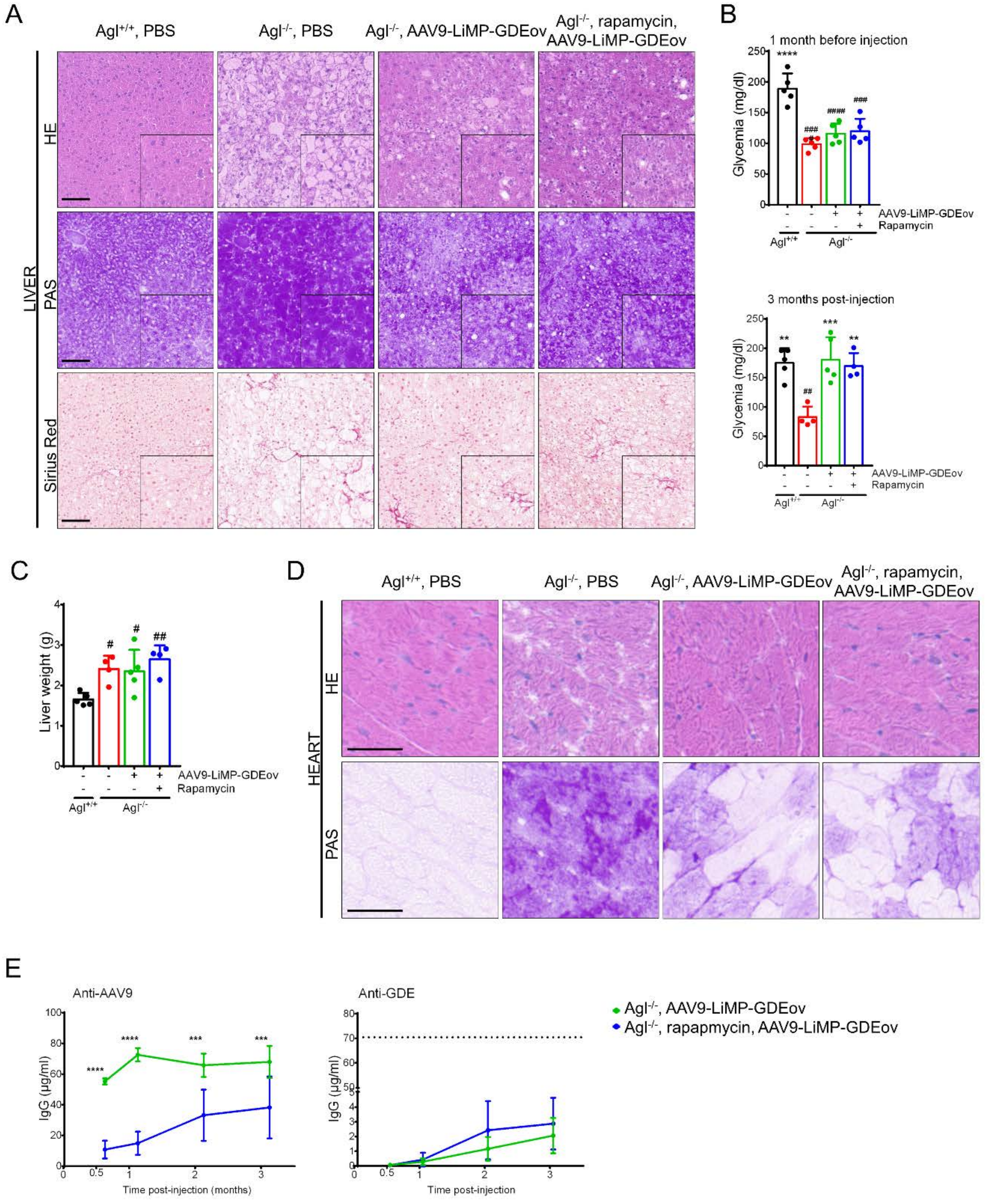

Supplementary Figure 4. 6-month-old *Agl*$^{-/-}$ mice received daily intraperitoneal injections of rapamycin at 1.5 mg/kg or vehicle for 6 weeks and were injected with AAV9-LiMP-GDEov at 6 x 10^{13} vg/kg (n = 4 per group) four weeks after the beginning of the rapamycin treatment. Vehicle-injected *Agl*$^{+/+}$ (n = 5) and *Agl*$^{-/-}$ (n = 4) mice received daily injection for 6 weeks. **A**. Hematoxylin and Eosin (HE), Periodic Acid Schiff (PAS), and Sirius red staining performed in liver sections; scale bar 100 µm. **B.** Glycemia measured one month before the beginning of the rapamycin treatment and three months after vector injection. **C.** Liver weight measured

three months after vector injection. **D.** Hematoxylin and Eosin (HE) and Periodic Acid Schiff (PAS) staining performed in heart; scale bar 50 µm. **E.** Circulating anti-AAV9 and anti-hGDE IgGs measured 0.5, 1-, 2-, or 3-months post-injection. Data shown as mean ± SD. Statistical analyses were performed by one-way ANOVA with Tuckey post-hoc test. Significance indicated with * vs. *Agl*$^{-/-}$ and $^{\#}$ vs. *Agl*$^{+/+}$. * $p<0.05$, ** and ## $p<0.01$, *** $p<0.001$, **** and #### $p<0.0001$.

SUPPLEMENTARY FIGURE 5

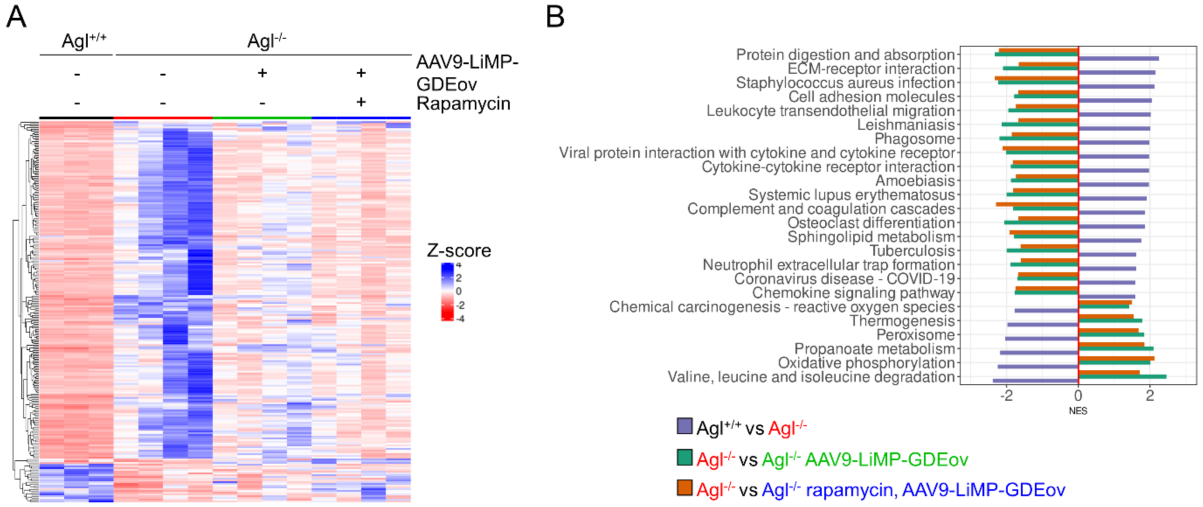

Supplementary Figure 5. A. Heatmap representing the differentially expressed genes (DEGs) among the three comparisons (*Agl*$^{-/-}$ vs. *Agl*$^{+/+}$, *Agl*$^{-/-}$ vs. *Agl*$^{-/-}$ AAV9-LiMP-GDEov, *Agl*$^{-/-}$ vs. *Agl*$^{-/-}$ AAV9-LiMP-GDEov, Rapamycin). Expression levels of up-regulated and down-regulated genes are represented in blue or red colors, respectively. **B.** Barplot representing the 24 common pathways found dysregulated between *Agl*$^{+/+}$ vs *Agl*$^{-/-}$ mice, *Agl*$^{-/-}$ vs *Agl*$^{-/-}$ AAV9-LiMP-GDEov mice and *Agl*$^{-/-}$ vs *Agl*$^{-/-}$ AAV9-LiMP-GDEov, Rapamycin mice found using the GSEA with KEGG database. NES, Normalized Enrichment Score.

SUPPLEMENTARY FIGURE 6

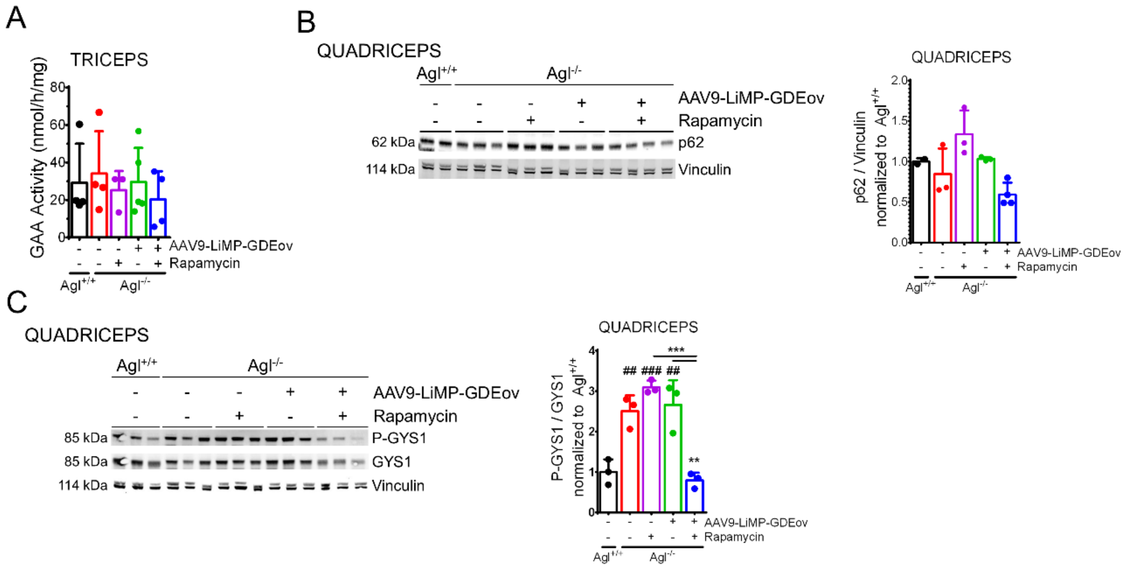

Supplementary Figure 6. 6-month-old *Agl*$^{-/-}$ mice received daily intraperitoneal injections of rapamycin at 1.5 mg/kg or vehicle for 6 weeks and were injected with AAV9-LiMP-GDEov at 6 x 10^{13} vg/kg (n = 4 per group) four weeks after the beginning of the rapamycin treatment. Vehicle-injected *Agl*$^{+/+}$ (n = 5) and *Agl*$^{-/-}$ (n = 4) mice received daily injection for 6 weeks. **A.** Acid alpha-glucosidase (GAA) activity in triceps. **B.** Western blot analysis of p62 in quadriceps. Quantifications of p62 protein bands are plotted next to the Western blot and expressed as ratio to vinculin. **C.** Western blot analysis of phosphorylated GYS (P-GYS) and GYS, and vinculin in quadriceps. Quantifications of P-GYS and GYS proteins bands are plotted next to the Western blot and expressed as ratio to GYS. Data shown as mean ± SD. Statistical analyses were performed by one-way ANOVA with Tuckey post-hoc test. Significance indicated with * vs. *Agl*$^{-/-}$ and # vs. *Agl*$^{+/+}$. ## and ** p<0.01, ### and *** p<0.01.

SUPPLEMENTARY FIGURE 7

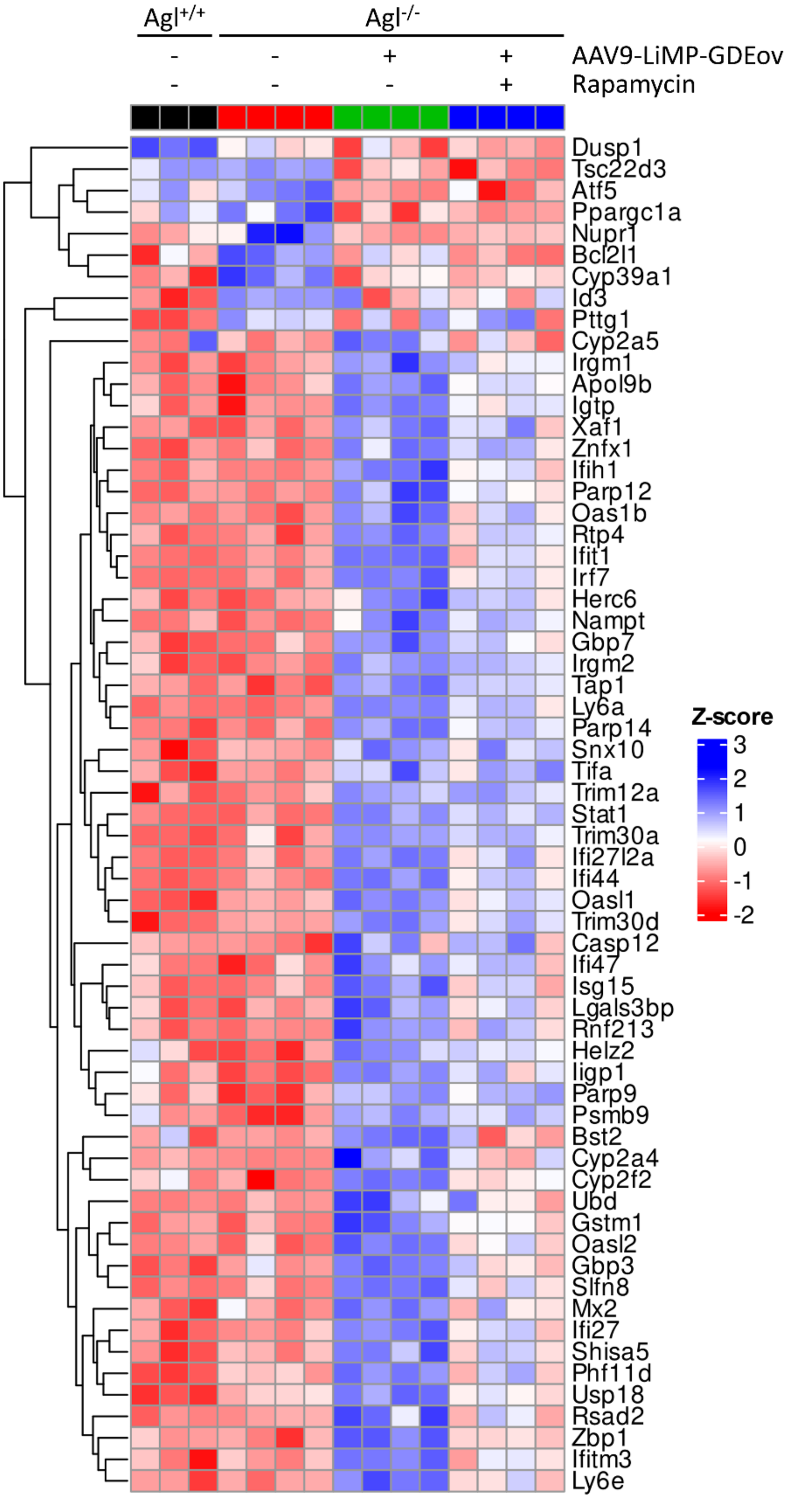

Supplementary Figure 7. Heatmap representing the differentially expressed genes (DEGs) related to IFN response among the three comparisons ($Agl^{-/-}$ vs. $Agl^{+/+}$, $Agl^{-/-}$ vs. $Agl^{-/-}$ AAV9-LiMP-GDEov, $Agl^{-/-}$ vs. $Agl^{-/-}$ AAV9-LiMP-GDEov, Rapamycin). Expression levels of

significatively up-regulated and down-regulated genes are represented in blue or red colors, respectively.

SUPPLEMENTARY FIGURE 8

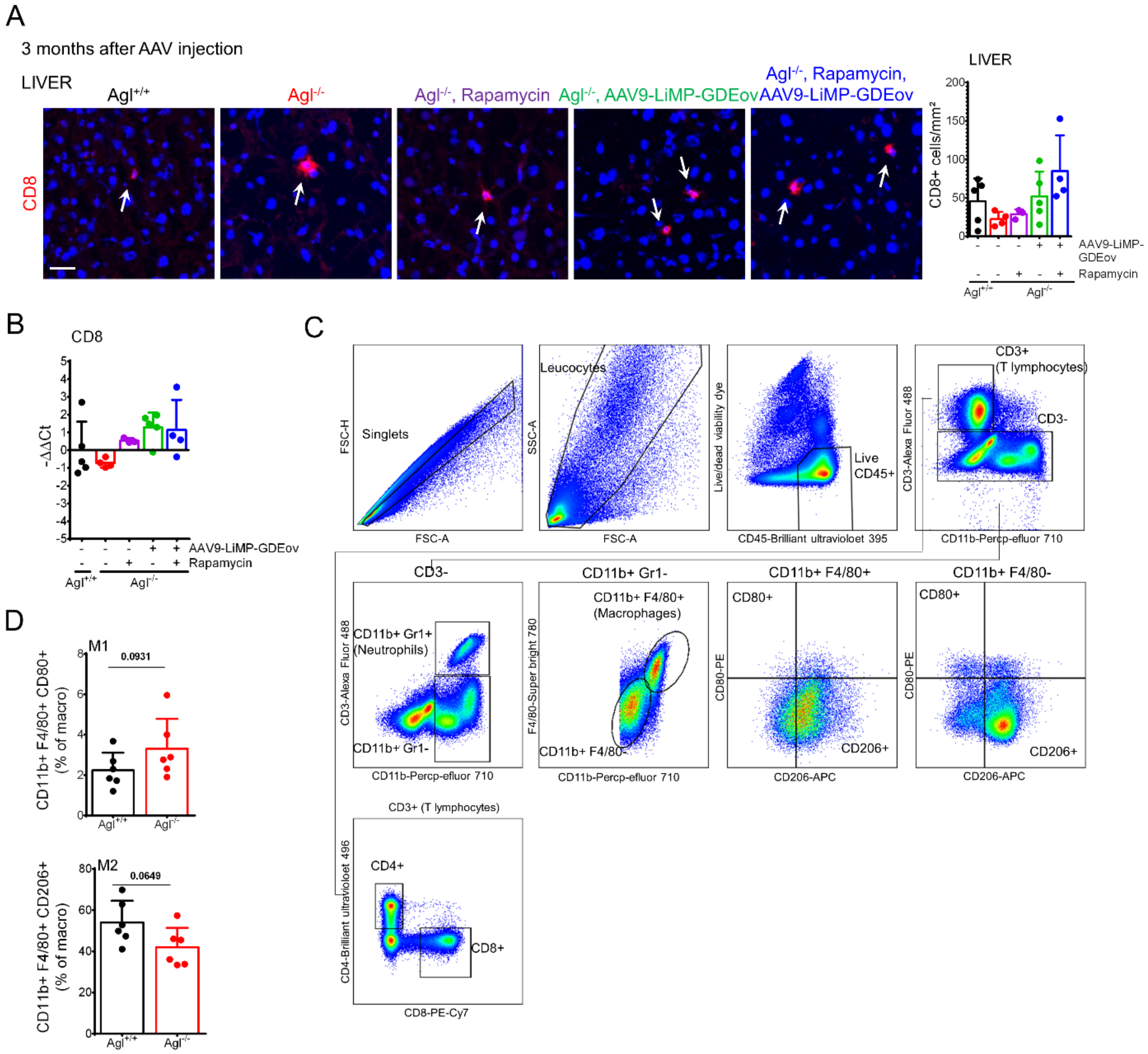

Supplementary Figure 8. Characterization of liver-infiltrating cells in liver of Agl$^{+/+}$ and Agl$^{-/-}$ mice **A.** Immunofluorescent DAPI staining of nucleus (blue) and CD8 positive cells (red) in liver at the end of the treatment ; scale bar 20 µm, and quantification of CD8 positive cells in liver section. White arrows indicate a cell positive for DAPI and CD8 staining. **B.** Transcriptomic levels of expression of CD8 in liver. **C.** Flow cytometry gating strategy for the identification of the hepatic immune cell populations. Livers of 6-month-old Agl$^{+/+}$ and Agl$^{-/-}$ mice (n=6 per group) were collected and liver infiltrating cell populations were mechanically extracted, labeled, and analyzed by flow cytometry. The gating strategy starts by eliminating the doublets (FSC-H v/s FSC-A). After gating on leucocytes according to their size and granularity (FSC v/s

SSC), dead and contaminating cells are eliminated by gating on live CD45+ cells. T lymphocytes are distinguished by their expression of CD3 (CD3 vs CD11b+) and gated into CD4+ and CD8+ T cells. Neutrophils (CD11b+ Gr1+) are then gated in the CD3 negative population. Macrophages are distinguished in the remaining CD11b+ Gr1- population as CD11b+ F4/80+, and characterized for their polarization profile through the expression of CD80 (M1) or CD206 (M2). **D.** Percentage of M1 and M2 polarized hepatic macrophages. Statistical analyses were performed by a one-way ANOVA (A and B) or t-test (D), p-value are indicated on the graphs. Data are shown as mean ± SD.

Supplementary Table 1. Vector genome copy number per diploid cell of all experiments.

		VCN/diploid cell								
**Genotype**	**Treatment**	**Liver**	**Heart**	**Quadriceps**	**Triceps**	**AC**	**PC**	**Psoas**	**Gluteus**	**Brain**
$Agl^{+/+}$	PBS	0,000±0,000	0,001± 0,001	0,004± 0,005	0,008± 0,007	0,024±0,008	0,008±0,003	0,028±0,015	0,001±0,001	0,000±0,000
$Agl^{-/-}$	PBS	0,000±0,000	0,002± 0,002	0,001± 0,001	0,009± 0,006	0,009±0,003	0,006±0,003	0,017±0,010	0,000±0,000	0,002±0,004
$Agl^{-/-}$	AAV-LiMP-GDEov	219,34±177,66	2,874± 1,743	0,610± 0,227	0,311± 0,150	1,195±0,439	0,437±0,195	7,275±2,018	1,008±0,416	0,120±0,055
$Agl^{+/+}$	Vehicle	0,001±0,001	0,002±0,001	0,008±0,009	0,053±0,095	n/a	n/a	n/a	n/a	n/a
$Agl^{-/-}$	Vehicle	0,001±0,000	0,002±0,001	0,014±0,020	0,059±0,098	n/a	n/a	n/a	n/a	n/a
$Agl^{-/-}$	Rapamycin	0,009±0,015	0,002±0,001	0,220±0,226	0,006±0,002	n/a	n/a	n/a	n/a	n/a
$Agl^{-/-}$	Vehicle, AAV-LiMP-GDEov	276,42±60,249	1,466±0,580	0,944±0,774	0,255±0,155	n/a	n/a	n/a	n/a	n/a
$Agl^{-/-}$	Rapamycin, AAV-LiMP-GDEov	96,500±71,673	1,202±0,643	1,527±1,049	0,315±0,077	n/a	n/a	n/a	n/a	n/a

AC: muscular anterior compartment of the leg, contains the tibialis anterior and EDL (extensor digitorum longus) muscles. PC: muscular posterior compartment of the leg, contains the gastrocnemius, soleus, and plantaris muscles. n/a: not applicable. Results are expressed as mean ± standard deviation.

Supplementary Table 2. List of differentially expressed genes (DEGs) found in pairwise comparisons between different conditions in triceps. DEGs identified among all the possible pairwise comparisons: untreated *Agl*$^{+/+}$ and *Agl*$^{-/-}$, and *Agl*$^{-/-}$ mice treated with AAV9-LiMP-GDEov alone or AAV9-LiMP-GDEov combined with rapamycin. Only genes with a Benjamini and Hochberg adjusted p-value < 0.05 and a fold-change superior to 1.5 or inferior to -1.5 are considered DEGs. 217 genes were found to be differentially expressed (DE) across all comparisons. 179 genes were found to be uniquely DE between the untreated *Agl*$^{+/+}$ and *Agl*$^{-/-}$ mice. 10 genes were found to be uniquely DE between the untreated *Agl*$^{-/-}$ and treated *Agl*$^{-/-}$, rapamycin + AAV9-LiMP-GDEov mice. 28 common genes were found to be DE across these two comparisons.

Comparison	Gene name	Log2FoldChange	p-value adjusted
***Agl*$^{+/+}$ vs *Agl*$^{-/-}$**	A930003A15Rik	4,06	3,01E-05
	Ablim1	1,39	4,04E-03
	Actc1	2,85	2,26E-07
	Adam19	1,75	3,64E-02
	Adgre1	1,80	2,24E-02
	Aebp1	1,33	4,68E-02
	Agl	-3,70	1,67E-38
	Alox12	2,34	2,66E-03
	Ankrd2	2,92	4,76E-24
	Anxa2	1,53	2,24E-06
	Anxa8	3,09	1,27E-10
	Apoe	1,59	1,06E-03
	Arl4c	3,17	1,40E-02
	Arpc1b	1,30	2,89E-02
	Asb14	-1,55	4,02E-03
	Aspn	1,92	3,16E-03
	Atp2a2	1,83	1,91E-04
	BC048679	-8,60	1,17E-09
	Bex1	1,50	2,47E-04
	C1qa	1,45	1,18E-02
	C1qtnf3	5,23	3,58E-04
	Card11	3,04	3,70E-02
	Ccl27a	-3,10	1,11E-04
	Cd44	2,45	1,72E-02
	Cd52	2,74	2,07E-03
	Cd74	1,95	3,07E-05
	Cdk6	1,96	1,88E-03
	Cdkn2a	3,92	1,65E-02
	Cers6	3,17	4,88E-02
	Chad	1,52	2,17E-02
	Chrng	7,66	8,68E-05
	Cilp2	2,17	3,06E-02
	Ckap4	1,80	5,17E-03
	Clec4d	3,72	3,32E-02
	Col14a1	2,44	3,29E-06

	Col1a1	2,27	2,88E-06
	Col5a2	1,98	9,46E-03
	Col6a2	1,58	1,58E-05
	Cotl1	1,66	1,87E-02
	Cpne2	1,75	5,77E-03
	Crlf3	2,04	4,84E-02
	Csrp3	2,62	5,68E-13
	Ctsk	2,05	9,83E-03
	Ctxn3	2,43	2,34E-04
	Cx3cl1	2,27	1,97E-02
	Cx3cr1	4,33	2,48E-03
	Cxcl14	1,41	6,33E-03
	Cyfip2	2,81	1,35E-03
	Dclk1	2,83	4,64E-06
	Dlg1	-1,14	1,12E-02
	E2f2	3,69	6,19E-03
	Eda2r	1,64	1,66E-02
	Fbln7	2,23	2,66E-04
	Fbn1	1,34	2,10E-02
	Fcgr3	1,81	2,97E-02
	Fhl2	2,29	2,21E-03
	Fstl1	1,50	7,71E-04
	Fxyd6	1,23	2,01E-02
	Gdf11	1,89	6,82E-03
	Gm13889	1,78	1,64E-03
	Gpm6b	1,59	4,86E-02
	Gpnmb	2,25	7,89E-03
	H2-Aa	2,43	2,51E-13
	H2-Ab1	2,29	1,63E-09
	H2-Eb1	2,20	2,97E-07
	Hbegf	1,62	2,36E-02
	Hsf2bp	3,88	2,34E-02
	Hspa1b	2,28	2,21E-03
	Hvcn1	3,76	9,04E-03
	Id2	1,69	3,51E-02
	Il13ra1	1,92	3,51E-02
	Il2rg	2,85	4,38E-03
	Ism1	3,19	4,78E-02
	Itgax	3,05	1,30E-02
	Itgbl1	1,96	4,84E-03
	Kbtbd11	2,31	4,06E-02
	Kcne1l	6,28	6,15E-04
	Klf5	1,85	2,88E-06
	Lamc2	3,35	9,18E-03
	Lamtor3	3,96	1,07E-02
	Laptm5	1,68	1,22E-02
	Lbh	2,14	3,56E-05
	Lgals3	2,96	5,91E-15
	Lilrb4b	2,66	4,99E-02
	Lmod2	1,62	8,78E-04
	Loxl1	1,53	6,54E-04
	Ltbp2	3,47	2,10E-02
	Lyz1	2,48	5,15E-08

	Lyz2	2,22	3,95E-13
	Maged2	1,71	3,57E-03
	Mamstr	-1,96	8,60E-03
	Map1a	2,12	5,88E-06
	Mertk	1,41	4,25E-02
	Mfap4	2,99	3,11E-03
	Mfap5	2,40	5,49E-12
	Mgp	2,44	1,97E-09
	Mmp14	1,52	2,22E-02
	Mmp2	1,52	7,90E-03
	Mpeg1	3,55	2,18E-12
	Mrln	-1,20	4,62E-02
	Msln	3,98	2,77E-03
	mt-Co1	-1,12	1,37E-02
	Mt1	2,40	2,24E-05
	Mtus2	4,32	2,66E-03
	Myl4	3,63	3,00E-14
	Myl6b	2,23	3,17E-02
	Mymx	7,92	3,90E-06
	Myom3	1,32	2,57E-02
	Ncam1	2,11	2,07E-03
	Ncf2	1,98	1,24E-02
	Nes	1,87	4,30E-08
	Neto2	-2,01	7,21E-05
	Nnat	-4,87	1,64E-04
	Npr3	1,75	4,92E-02
	Oplah	-2,87	3,13E-02
	Opn1mw	3,33	6,64E-05
	Osbpl3	2,37	1,51E-02
	Ostn	2,18	2,53E-02
	Oxsr1	-1,51	2,66E-04
	P4ha3	5,07	1,78E-03
	Pam	1,67	2,70E-03
	Pamr1	2,73	1,18E-02
	Pcca	-1,27	1,22E-02
	Pdgfrl	2,46	4,54E-02
	Peg3	2,42	1,70E-02
	Plaat1	-1,20	2,03E-02
	Plekho1	1,45	8,39E-03
	Ppp1r3a	-1,07	4,19E-02
	Prss12	3,77	5,17E-03
	Prune2	4,14	9,44E-14
	Pttg1	4,27	9,28E-23
	Ptx4	3,15	3,86E-04
	Pycard	2,91	2,48E-02
	Rab11fip5	1,23	2,01E-02
	Rab15	5,25	1,31E-03
	Rasd2	-2,10	7,70E-05
	Rassf4	1,69	3,30E-02
	Rbms3	1,31	3,01E-02
	Rcan1	1,45	5,26E-04
	Rhoc	1,35	1,37E-02
	Rhou	-1,58	7,72E-04

	Rrad	2,83	4,90E-06
	Runx1	3,60	9,91E-10
	S100a13	1,27	4,33E-02
	S1pr2	2,39	1,73E-03
	Selplg	2,26	2,22E-02
	Serpina3n	2,18	6,73E-03
	Serpinb1a	3,20	6,10E-09
	Sgms2	2,21	1,30E-02
	Smim3	2,17	1,22E-02
	Sod3	1,28	1,97E-02
	Sparc	1,38	2,10E-03
	Spsb4	3,74	1,85E-03
	Ssc5d	2,08	1,12E-02
	St6galnac4	1,75	1,53E-03
	Sulf1	1,64	2,07E-03
	Thbs4	3,52	7,80E-03
	Tlcd3b	-1,89	1,20E-05
	Tlr13	4,99	2,11E-03
	Tmem267	-2,28	2,60E-09
	Tnk2	-1,19	4,64E-02
	Tnnt1	2,45	4,51E-02
	Tnnt2	2,71	1,97E-09
	Top2a	3,31	3,60E-02
	Tppp3	1,28	1,65E-02
	Trem2	2,49	2,10E-02
	Trim47	1,81	3,56E-05
	Ttc9	2,69	1,02E-03
	Tuba1b	1,14	4,19E-02
	Tubb2b	2,20	7,54E-03
	Tubb6	2,60	4,65E-11
	Tyrobp	1,71	2,07E-03
	Ubash3b	2,84	3,06E-02
	Uchl1	2,90	8,92E-03
	Uck2	1,18	2,52E-02
	Ucp2	1,55	3,43E-02
	Vcam1	1,76	3,86E-02
	Wnt4	-1,55	2,66E-02
	Xirp1	1,43	5,04E-03
**$Agl^{-/-}$ vs $Agl^{-/-}$, rapamycin + AAV9-LiMP-GDEov**	Adamts4	-4,02	3,40E-03
	Adrb3	4,07	3,82E-03
	Cdc14a	1,87	8,32E-03
	Col5a3	-1,41	4,30E-02
	Gal	-4,45	6,79E-03
	Gfra2	-2,15	4,30E-02
	Ppl	1,97	3,76E-02
	Spon2	-2,99	3,09E-04
	Timp1	-2,62	2,15E-04
	Tuba1c	-1,58	3,00E-02
**$Agl^{+/+}$ vs $Agl^{-/-}$ & $Agl^{-/-}$ vs $Agl^{-/-}$, rapamycin + AAV9-LiMP-GDEov**	Adcy7	1,91	1,19E-02
	Bgn	2,12	8,37E-11
	C1qb	1,96	1,81E-02
	Casq2	2,04	2,76E-04
	Chodl	1,36	3,38E-02

	Cilp	3,61	8,87E-28
	Col8a1	3,39	5,49E-12
	Csf2rb	2,77	1,11E-06
	Ctss	2,39	6,26E-06
	Fcer1g	1,71	6,06E-03
	Fkbp10	2,24	6,03E-03
	Fn1	1,90	1,31E-03
	Hspb7	1,90	7,13E-08
	Lox	2,21	1,62E-06
	Ms4a6d	2,29	1,24E-05
	Mt2	2,79	5,00E-03
	Myh3	5,59	1,16E-35
	Myh8	4,88	5,02E-21
	Mymk	6,93	5,58E-12
	Myog	3,32	2,11E-07
	Pnma8b	7,79	4,18E-06
	Postn	4,24	1,49E-15
	S100a4	2,70	7,33E-07
	Sh3pxd2b	1,84	3,13E-02
	Sln	4,34	5,86E-15
	Sox11	6,26	3,28E-16
	Tceal7	1,75	2,88E-06
	Tnc	3,84	6,01E-10

A negative log2foldChange means that this gene is under-expressed in the second term of the comparison (under-expressed in *Agl*$^{-/-}$ condition in untreated *Agl*$^{+/+}$ versus *Agl*$^{-/-}$ mice comparison).

Supplementary Table 3. Validation of transcriptomics results by comparison of 9 genes expression quantified by qPCR, in liver. RNA-seq results are expressed as a number of reads, and qPCR results are expressed in n fold. Gm32468, Lepr, and Asns are upregulated in *Agl*$^{-/-}$ mice. Obp2a, Gm3787, Noct, Agl are downregulated in *Agl*$^{-/-}$ mice. Pttg1 and Mpeg1 are commonly differentially expressed in liver and triceps (supplementary table 4).

	**Gm32468**		**Lepr**		**Asns**		**Obp2a**		**Gm3787**		**Noct**		**Agl**		**Pttg1**		**Mpeg1**	
**Genotype**	RNA-seq	qPCR	RNA-seq	qPCR	RNA-seq	qPCR	RNA-seq	qPCR	RNA-seq	qPCR	RNA-seq	qPCR	RNA-seq	qPCR	RNA-seq	qPCR	RNA-seq	qPCR
	0,00	0,315	0,000	0,422	0,000	1,118	185,095	1,752	121,571	0,646	1859,712	0,440	307,762	0,631	32,857	0,601	443,571	1,228
**Agl$^{+/+}$**	0,00	0,692	1,138	2,538	0,000	3,646	73,978	1,447	101,293	1,293	1084,627	1,383	298,187	1,904	28,453	1,656	261,767	2,009
	0,00	4,806	0,992	0,953	1,984	1,269	66,471	0,395	251,002	1,210	1973,294	1,673	301,600	0,833	59,526	1,007	223,223	0,406
	210,12	79,487	42,449	12,748	53,062	2,302	9,551	0,045	15,918	0,040	278,042	0,274	36,082	0,011	501,962	6,888	965,720	2,111
**Agl$^{-/-}$**	59,37	40,250	71,870	14,317	8,593	7,985	2,344	0,007	50,778	0,117	346,070	0,241	30,467	0,010	264,045	3,303	1080,395	1,784
	41,40	8,312	58,733	12,908	51,030	4,546	9,628	0,039	2,888	0,048	298,478	0,248	14,442	0,006	303,292	3,086	1357,594	2,453
	174,67	46,598	35,665	9,003	82,304	6,772	5,487	0,029	22,862	0,053	385,916	0,194	55,784	0,003	275,262	3,493	1039,778	1,911

Gm32468: predicted gene 32468 of Mus musculus. Lepr: leptin receptor. Asns: asparagine synthetase. Obp2a: odorant binding protein 2A. Gm3787: predicted gene 3787 of Mus musculus. Noct: nocturnin. Agl: amylo-alpha-1, 6-glucosidase, 4-alpha-glucanotransferase. Pttg1: pituitary tumor transforming gene 1. Mpeg1: macrophage-expressed gene 1.

Supplementary Table 4. Validation of transcriptomics results by comparison of 9 genes expression quantified by qPCR, in triceps. RNA-seq results are expressed as a number of reads, and qPCR results are expressed in n fold. Mymx, PnmaI, and Chrng are upregulated in *Agl*$^{-/-}$ mice. BC048679, Nnat, Agl, and GM15543 are downregulated in *Agl*$^{-/-}$ mice. Pttg1 and Mpeg1 are commonly differentially expressed in liver and triceps (supplementary table 3).

	**Mymx**		**Pnma1**		**Chrng**		**BC048679**		**Nnat**		**Agl**		**Gm15543**		**Pttg1**		**Mpeg1**	
**Genotype**	RNA-seq	qPCR	RNA-seq	qPCR	RNA-seq	qPCR	RNA-seq	qPCR	RNA-seq	qPCR	RNA-seq	qPCR	RNA-seq	qPCR	RNA-seq	qPCR	RNA-seq	qPCR
	0,000	1,049	0,000	0,731	0,000	0,581	77,658	0,600	89,249	1,226	7748,413	0,641	252,678	0,718	40,568	0,701	41,727	1,195
**Agl$^{+/+}$**	0,000	1,328	0,000	1,702	0,000	2,541	57,756	1,094	42,739	1,397	6542,576	2,023	232,178	1,501	19,637	1,770	54,290	2,215
	0,000	0,848	0,000	0,820	0,000	0,742	170,326	1,530	43,375	0,587	6843,714	0,774	248,612	0,930	16,927	0,809	21,158	0,391
	22,465	33,711	37,068	1,446	10,109	2,922	0,000	0,007	0,000	0,053	712,152	0,029	68,519	0,033	699,796	11,787	416,732	2,196
**Agl$^{-/-}$**	53,143	30,829	50,244	2,077	24,156	5,311	0,000	0,009	6,764	0,069	579,739	0,028	16,426	0,009	491,812	6,306	226,098	2,801
	57,650	80,196	43,589	2,381	32,340	9,460	0,703	0,016	1,406	0,047	528,693	0,032	17,576	0,029	408,472	7,437	530,803	5,252
	66,625	64,560	51,079	3,389	99,937	9,665	0,000	0,003	0,000	0,064	350,889	0,019	9,624	0,052	376,059	5,423	649,219	3,214

Mymx: myomixer, myoblast fusion factor. Pnma1: paraneoplastic Ma antigen 1. Chrng: cholinergic receptor nicotinic gamma subunit. BC048679: cDNA sequence BC048679 from Mus musculus. Nnat: neuronatin. Agl: amylo-alpha-1, 6-glucosidase, 4-alpha-glucanotransferase. Gm15543: predicted gene 15543 of Mus musculus. Pttg1: pituitary tumor transforming gene 1. Mpeg1: macrophage-expressed gene 1.

Supplementary Table 5. List of genes grouped in each cluster defined by k-means clustering analysis on the genes belonging to the “Lysosome” pathway. K-means clustering analysis has been performed between *Agl*$^{-/-}$ mice treated with rapamycin alone, AAV9-LiMP-GDEov alone or that received the combined treatment. Genes were assigned to three different clusters: 1) modified by the treatment, 2) modified by the disease, not rescued and, 3) modified by the disease, rescued.

Condition	Cluster	Gene
***Agl*$^{-/-}$, rapamycin**	Modified by the treatment	Acp2
	Modified by the treatment	Ap1g2
	Modified by the treatment	Ap1m2
	Modified by the treatment	Ap4e1
	Modified by the treatment	Ap4m1
	Modified by the treatment	Arsa
	Modified by the treatment	Atp6v0a4
	Modified by the treatment	Atp6v0c
	Modified by the treatment	Ctsw
	Modified by the treatment	Galc
	Modified by the treatment	Gba
	Modified by the treatment	Gga2
	Modified by the treatment	Gla
	Modified by the treatment	Hexb
	Modified by the treatment	Hgsnat
	Modified by the treatment	Manba
	Modified by the treatment	Tcirg1
	Not rescued	Abcb9
	Not rescued	Acp5
	Not rescued	Aga
	Not rescued	Ap1b1
	Not rescued	Ap1s3
	Not rescued	Ap3b1
	Not rescued	Ap3m1
	Not rescued	Ap3s1
	Not rescued	Arsb
	Not rescued	Asah1
	Not rescued	Atp6ap1
	Not rescued	Atp6v0d1
	Not rescued	Atp6v0d2
	Not rescued	Atp6v1h
	Not rescued	Cd63
	Not rescued	Cd68
	Not rescued	Cln5
	Not rescued	Clta
	Not rescued	Cltb
	Not rescued	Cltc
	Not rescued	Ctns

	Not rescued	Ctsa
	Not rescued	Ctsc
	Not rescued	Ctsd
	Not rescued	Ctse
	Not rescued	Ctsh
	Not rescued	Ctsk
	Not rescued	Ctsl
	Not rescued	Ctss
	Not rescued	Ctsz
	Not rescued	Dmxl1
	Not rescued	Dnase2a
	Not rescued	Dnase2b
	Not rescued	Galns
	Not rescued	Gga1
	Not rescued	Glb1
	Not rescued	Gm2a
	Not rescued	Gnptab
	Not rescued	Gusb
	Not rescued	Hexa
	Not rescued	Hyal3
	Not rescued	Idua
	Not rescued	Igf2r
	Not rescued	Lamp1
	Not rescued	Lamp2
	Not rescued	Laptm4b
	Not rescued	Laptm5
	Not rescued	Lipa
	Not rescued	Litaf
	Not rescued	Man2b1
	Not rescued	Mcoln1
	Not rescued	Mfsd8
	Not rescued	Naga
	Not rescued	Naglu
	Not rescued	Napsa
	Not rescued	Ncoa7
	Not rescued	Neu1
	Not rescued	Npc1
	Not rescued	Npc2
	Not rescued	Pla2g15
	Not rescued	Ppt1
	Not rescued	Ppt2
	Not rescued	Psap
	Not rescued	Sgsh
	Not rescued	Slc11a1
	Not rescued	Sort1

	Not rescued	Tpp1
	Not rescued	Wdr7
	Rescued	Abca2
	Rescued	Ap1g1
	Rescued	Ap1m1
	Rescued	Ap1s1
	Rescued	Ap1s2
	Rescued	Ap3b2
	Rescued	Ap3d1
	Rescued	Ap3m2
	Rescued	Ap3s2
	Rescued	Ap4b1
	Rescued	Ap4s1
	Rescued	Arsg
	Rescued	Atp6v0a1
	Rescued	Atp6v0a2
	Rescued	Atp6v0b
	Rescued	Cd164
	Rescued	Cln3
	Rescued	Ctsb
	Rescued	Ctsf
	Rescued	Ctsg
	Rescued	Ctso
	Rescued	Dmxl2
	Rescued	Fuca1
	Rescued	Fuca2
	Rescued	Gaa
	Rescued	Gga3
	Rescued	Gnptg
	Rescued	Gns
	Rescued	Hyal1
	Rescued	Hyal2
	Rescued	Ids
	Rescued	Laptm4a
	Rescued	Lgmn
	Rescued	M6pr
	Rescued	Nagpa
	Rescued	Scarb2
	Rescued	Slc11a2
	Rescued	Slc17a5
	Rescued	Smpd1
	Rescued	Sumf1
***Agl*$^{-/-}$, AAV9-LiMP-GDEov**	Modified by the treatment	Acp2
	Modified by the treatment	Ap1g2
	Modified by the treatment	Ap4e1

	Modified by the treatment	Arsb
	Modified by the treatment	Atp6v1h
	Modified by the treatment	Ctns
	Modified by the treatment	Gga2
	Modified by the treatment	Gla
	Modified by the treatment	Hexb
	Modified by the treatment	Hgsnat
	Modified by the treatment	Igf2r
	Modified by the treatment	M6pr
	Modified by the treatment	Npc1
	Modified by the treatment	Scarb2
	Modified by the treatment	Slc11a1
	Modified by the treatment	Slc17a5
	Modified by the treatment	Tcirg1
	Not rescued	Abca2
	Not rescued	Abcb9
	Not rescued	Acp5
	Not rescued	Ap1g1
	Not rescued	Ap1s1
	Not rescued	Ap1s2
	Not rescued	Ap3b1
	Not rescued	Ap3m1
	Not rescued	Ap3m2
	Not rescued	Ap3s1
	Not rescued	Atp6v0a4
	Not rescued	Atp6v0d1
	Not rescued	Atp6v0d2
	Not rescued	Cd63
	Not rescued	Cd68
	Not rescued	Cln3
	Not rescued	Clta
	Not rescued	Cltc
	Not rescued	Ctsa
	Not rescued	Ctsc
	Not rescued	Ctse
	Not rescued	Ctsh
	Not rescued	Ctso
	Not rescued	Ctss
	Not rescued	Ctsz
	Not rescued	Dmxl2
	Not rescued	Dnase2a
	Not rescued	Dnase2b
	Not rescued	Galns
	Not rescued	Gba
	Not rescued	Gm2a

	Not rescued	Gnptab
	Not rescued	Gns
	Not rescued	Hexa
	Not rescued	Hyal1
	Not rescued	Lamp2
	Not rescued	Laptm4a
	Not rescued	Laptm5
	Not rescued	Lgmn
	Not rescued	Man2b1
	Not rescued	Mcoln1
	Not rescued	Mfsd8
	Not rescued	Nagpa
	Not rescued	Ncoa7
	Not rescued	Neu1
	Not rescued	Ppt1
	Not rescued	Psap
	Not rescued	Sgsh
	Not rescued	Sumf1
	Rescued	Aga
	Rescued	Ap1b1
	Rescued	Ap1m1
	Rescued	Ap1m2
	Rescued	Ap1s3
	Rescued	Ap3b2
	Rescued	Ap3d1
	Rescued	Ap3s2
	Rescued	Ap4b1
	Rescued	Ap4m1
	Rescued	Ap4s1
	Rescued	Arsa
	Rescued	Arsg
	Rescued	Asah1
	Rescued	Atp6ap1
	Rescued	Atp6v0a1
	Rescued	Atp6v0a2
	Rescued	Atp6v0b
	Rescued	Cd164
	Rescued	Cln5
	Rescued	Cltb
	Rescued	Ctsb
	Rescued	Ctsd
	Rescued	Ctsf
	Rescued	Ctsg
	Rescued	Ctsk
	Rescued	Ctsl

	Rescued	Ctsw
	Rescued	Dmxl1
	Rescued	Fuca1
	Rescued	Fuca2
	Rescued	Gaa
	Rescued	Galc
	Rescued	Gga1
	Rescued	Gga3
	Rescued	Glb1
	Rescued	Gnptg
	Rescued	Gusb
	Rescued	Hyal2
	Rescued	Hyal3
	Rescued	Ids
	Rescued	Idua
	Rescued	Lamp1
	Rescued	Laptm4b
	Rescued	Lipa
	Rescued	Litaf
	Rescued	Manba
	Rescued	Naga
	Rescued	Naglu
	Rescued	Napsa
	Rescued	Npc2
	Rescued	Pla2g15
	Rescued	Ppt2
	Rescued	Slc11a2
	Rescued	Smpd1
	Rescued	Sort1
	Rescued	Tpp1
	Rescued	Wdr7
***Agl$^{-/-}$*, rapamycin + AAV9-LiMP-GDEov**	Modified by the treatment	Acp2
	Modified by the treatment	Aga
	Modified by the treatment	Ap3b2
	Modified by the treatment	Ap4m1
	Modified by the treatment	Arsb
	Modified by the treatment	Atp6v0c
	Modified by the treatment	Cltc
	Modified by the treatment	Ctns
	Modified by the treatment	Dmxl1
	Modified by the treatment	Hgsnat
	Modified by the treatment	Igf2r
	Modified by the treatment	M6pr
	Modified by the treatment	Naga
	Modified by the treatment	Npc1

	Modified by the treatment	Scarb2
	Not rescued	Abcb9
	Not rescued	Ap1b1
	Not rescued	Ap1g2
	Not rescued	Ap1m2
	Not rescued	Ap1s1
	Not rescued	Ap1s2
	Not rescued	Ap3b1
	Not rescued	Arsa
	Not rescued	Atp6ap1
	Not rescued	Atp6v0a4
	Not rescued	Atp6v0d1
	Not rescued	Atp6v1h
	Not rescued	Ctsa
	Not rescued	Ctsc
	Not rescued	Ctsd
	Not rescued	Ctsl
	Not rescued	Dnase2a
	Not rescued	Galc
	Not rescued	Gba
	Not rescued	Gga1
	Not rescued	Gla
	Not rescued	Gnptab
	Not rescued	Hyal1
	Not rescued	Laptm4a
	Not rescued	Mcoln1
	Not rescued	Nagpa
	Not rescued	Ncoa7
	Not rescued	Sgsh
	Not rescued	Slc11a1
	Not rescued	Slc17a5
	Not rescued	Sort1
	Rescued	Abca2
	Rescued	Acp5
	Rescued	Ap1g1
	Rescued	Ap1m1
	Rescued	Ap1s3
	Rescued	Ap3d1
	Rescued	Ap3m1
	Rescued	Ap3m2
	Rescued	Ap3s1
	Rescued	Ap3s2
	Rescued	Ap4b1
	Rescued	Ap4e1
	Rescued	Ap4s1

	Rescued	Arsg
	Rescued	Asah1
	Rescued	Atp6v0a1
	Rescued	Atp6v0a2
	Rescued	Atp6v0b
	Rescued	Atp6v0d2
	Rescued	Cd164
	Rescued	Cd63
	Rescued	Cd68
	Rescued	Cln3
	Rescued	Cln5
	Rescued	Clta
	Rescued	Cltb
	Rescued	Ctsb
	Rescued	Ctse
	Rescued	Ctsf
	Rescued	Ctsg
	Rescued	Ctsh
	Rescued	Ctsk
	Rescued	Ctso
	Rescued	Ctss
	Rescued	Ctsw
	Rescued	Ctsz
	Rescued	Dmxl2
	Rescued	Dnase2b
	Rescued	Fuca1
	Rescued	Fuca2
	Rescued	Gaa
	Rescued	Galns
	Rescued	Gga2
	Rescued	Gga3
	Rescued	Glb1
	Rescued	Gm2a
	Rescued	Gnptg
	Rescued	Gns
	Rescued	Gusb
	Rescued	Hexa
	Rescued	Hyal2
	Rescued	Hyal3
	Rescued	Ids
	Rescued	Idua
	Rescued	Lamp1
	Rescued	Lamp2
	Rescued	Laptm4b
	Rescued	Laptm5

	Rescued	Lgmn
	Rescued	Lipa
	Rescued	Litaf
	Rescued	Man2b1
	Rescued	Manba
	Rescued	Mfsd8
	Rescued	Naglu
	Rescued	Napsa
	Rescued	Neu1
	Rescued	Npc2
	Rescued	Pla2g15
	Rescued	Ppt1
	Rescued	Ppt2
	Rescued	Psap
	Rescued	Slc11a2
	Rescued	Smpd1
	Rescued	Sumf1
	Rescued	Tcirg1
	Rescued	Tpp1
	Rescued	Wdr7

Supplementary Table 6. Description of proteins and corresponding genes reported in Figures 5 and 6.

Protein	NCBI gene ID	Symbol	Name
ABCA2	11305	Abca2	ATP-binding cassette, sub-family A member 2
ABCB9	56325	Abcb9	ATP-binding cassette, sub-family B member 9
ACP2	11432	Acp2	acid phosphatase 2, lysosomal
ACP5	11433	Acp5	acid phosphatase 5, tartrate resistant
AGA	11593	Aga	aspartylglucosaminidase
AP-1	108012	Ap1s2	adaptor-related protein complex 1, sigma 2 subunit
AP-3	11774	Ap3b1	adaptor-related protein complex 3, beta 1 subunit
AP-4	108011	Ap4e1	adaptor-related protein complex AP-4, epsilon 1
ARS	11881	Arsb	arylsulfatase B
ASAH1	11886	Asah1	N-acylsphingosine amidohydrolase 1
ATPeV	108664	Atp6v1h	ATPase, H+ transporting, lysosomal V1 subunit H
cathepsins	13030	Ctsb	cathepsin B
clathrin	12757	Clta	clathrin light chain A
CLN1	19063	Ppt1	palmitoyl-protein thioesterase 1
CLN3	12752	Cln3	CLN3 lysosomal/endosomal transmembrane protein, battenin
CLN5	211286	Cln5	ceroid-lipofuscinosis, neuronal 5
CLN7	72175	Mfsd8	major facilitator superfamily domain containing 8
cystinosin	83429	Ctns	cystinosis, nephropathic
DMXL	235380	Dmxl2	Dmx-like 2
DnaseII	13423	Dnase2a	deoxyribonuclease II alpha
endolyn	53599	Cd164	CD164 antigen
FGE	58911	Sumf1	sulfatase modifying factor 1
FUCA1	66848	Fuca2	fucosidase, alpha-L- 2, plasma
GAA	14387	Gaa	glucosidase, alpha, acid
GALC	14420	Galc	galactosylceramidase
GALNS	50917	Galns	galactosamine (N-acetyl)-6-sulfatase
GBA	14466	Gba1	glucosylceramidase beta 1
GGAs	106039	Gga1	golgi associated, gamma adaptin ear containing, ARF binding protein 1
GLA	11605	Gla	galactosidase, alpha
GLB	12091	Glb1	galactosidase, beta 1
GM2A	14667	Gm2a	GM2 ganglioside activator protein
GNPT	214505	Gnptg	N-acetylglucosamine-1-phosphotransferase, gamma subunit
GNS	75612	Gns	glucosamine (N-acetyl)-6-sulfatase
GUSB	110006	Gusb	glucuronidase, beta
HEXA/B	15211	Hexa	hexosaminidase A
HGSNAT	52120	Hgsnat	heparan-alpha-glucosaminide N-acetyltransferase
HYAL1	109685	Hyal3	hyaluronoglucosaminidase 3
IDS	15931	Ids	iduronate 2-sulfatase
IDUA	15932	Idua	iduronidase, alpha-L
LALP70	100862375	Entpd4b	ectonucleoside triphosphate diphosphohydrolase 4B
LAMAN	17159	Man2b1	mannosidase 2, alpha B1
LAMP	12514	Cd68	CD68 antigen
LAPTM	114128	Laptm4b	lysosomal-associated protein transmembrane 4B
LGMN	19141	Lgmn	legumain
LIMP	12492	Scarb2	scavenger receptor class B, member 2

LIPA	16889	Lipa	lysosomal acid lipase A
LITAF	56722	Litaf	LPS-induced TN factor
LYPLA3	192654	Pla2g15	Glycerophospholipid metabolism
MANB	110173	Manba	mannosidase, beta A, lysosomal
MCOLN1	94178	Mcoln1	mucolipin 1
MPR	16004	Igf2r	insulin-like growth factor 2 receptor
NAGA	17939	Naga	N-acetyl galactosaminidase, alpha
NAGLU	27419	Naglu	alpha-N-acetylglucosaminidase (Sanfilippo disease IIIB)
NAGPA	27426	Nagpa	N-acetylglucosamine-1-phosphodiester alpha-N-acetylglucosaminidase
napsin	16541	Napsa	napsin A aspartic peptidase
NCOA7	211329	Ncoa7	nuclear receptor coactivator 7
NEU1	18010	Neu1	neuraminidase 1
NPC	18145	Npc1	NPC intracellular cholesterol transporter 1
NRAMP	18173	Slc11a1	solute carrier family 11 (proton-coupled divalent metal ion transporters), member 1
saposin	19156	Psap	prosaposin
SGSH	27029	Sgsh	N-sulfoglucosamine sulfohydrolase (sulfamidase)
sialin	235504	Slc17a5	solute carrier family 17 (anion/sugar transporter), member 5
SMPD1	20597	Smpd1	sphingomyelin phosphodiesterase 1, acid lysosomal
sortilin	20661	Sort1	sortilin 1
WDR7	104082	Wdr7	WD repeat domain 7

Supplementary Table 7. List of differentially expressed genes (DEGs) found in pairwise comparisons between different conditions in liver. DEGs identified among all pairwise comparisons: untreated *Agl*$^{+/+}$ and *Agl*$^{-/-}$, and *Agl*$^{-/-}$ mice treated with AAV9-LiMP-GDEov alone or AAV9-LiMP-GDEov combined with rapamycin. Only genes with a Benjamini and Hochberg adjusted p-value < 0.05 and a fold-change superior to 1.5 or inferior to -1.5 are considered DEGs. 139 genes were found to be differentially expressed (DE) across all comparisons. 24 were found to be uniquely DE between the untreated *Agl*$^{-/-}$ and *Agl*$^{+/+}$ mice. 71 genes were found to be uniquely DE between untreated *Agl*$^{-/-}$ and *Agl*$^{-/-}$ AAV9-LiMP-GDEov treated mice. 18 genes were found to be uniquely DE between the untreated *Agl*$^{-/-}$ and *Agl*$^{-/-}$, rapamycin + AAV9-LiMP-GDEov treated mice. 3 common genes were found to be DE across two comparisons: the untreated *Agl*$^{+/+}$ versus *Agl*$^{-/-}$ mice and the untreated *Agl*$^{-/-}$ versus *Agl*$^{-/-}$ AAV9-LiMP-GDEov treated mice. 3 common genes were found to be DE across all pairwise comparisons.

Comparison	Gene name	Log2FoldChange	p-value adjusted
***Agl*$^{+/+}$ vs *Agl*$^{-/-}$**	2010003K11Rik	1,64	4,24E-02
	Acaca	1,36	4,62E-02
	Asns	6,11	2,30E-03
	Cfap20dc	5,33	2,97E-07
	Cyp2c23	2,65	8,16E-04
	Cyp2u1	-1,62	3,94E-04
	Cyp39a1	2,82	4,24E-02
	Dusp1	-1,62	5,61E-05
	Elovl6	1,64	3,41E-04
	Fam89a	1,53	9,01E-03
	Guk1	1,13	4,62E-02
	Hcn3	1,84	1,92E-02
	Id3	1,44	7,05E-06
	Map3k5	1,26	7,58E-03
	Mpeg1	1,85	1,50E-06
	Mup21	-2,10	2,02E-06
	Noct	-2,32	7,26E-14
	Obp2a	-4,03	3,75E-07
	Pebp1	-1,19	2,23E-04
	Pttg1	3,05	6,09E-12
	Tbc1d31	2,00	2,82E-03
	Tceal8	2,04	4,62E-02
	Tent5a	2,01	2,10E-04
	Tstd1	-1,38	1,92E-02
***Agl*$^{-/-}$ vs *Agl*$^{-/-}$ AAV9-LiMP-GDEov**	Abcc3	1,36	1,80E-02
	Acly	-1,12	2,99E-03
	Aox1	1,41	1,23E-02
	Apol9a	1,85	2,06E-02
	Apol9b	1,70	7,56E-06
	Atf5	-0,99	8,54E-03
	Bst2	1,34	9,66E-05
	Camk1d	-1,18	7,10E-03

	Casp12	2,45	2,86E-02
	Ccdc162	-1,53	3,61E-03
	Chrna4	-2,21	1,76E-03
	Cyp2a4	8,69	3,59E-11
	Cyp2a5	1,32	3,94E-05
	Cyp2c68	-1,40	1,07E-02
	Cyp2f2	0,98	3,41E-03
	Ddah1	2,94	1,80E-02
	Dntt	-1,98	4,24E-05
	Eif4e3	1,95	4,74E-02
	Elovl5	-0,88	3,10E-02
	Gbp3	2,05	3,29E-03
	Gbp7	1,42	9,42E-04
	Gm3776	3,36	2,15E-04
	Gsta2	2,00	2,06E-02
	Gstm1	2,01	8,31E-14
	Helz2	1,08	1,09E-02
	Herc6	1,85	2,99E-03
	Ifi27	1,95	1,68E-17
	Ifi27l2a	2,81	6,38E-09
	Ifi47	1,42	1,22E-02
	Ifih1	1,60	1,79E-08
	Ifit1	3,01	1,39E-37
	Ifit3	1,82	1,26E-06
	Ifitm3	0,79	4,24E-02
	Igtp	2,32	3,37E-13
	Iigp1	1,25	6,28E-11
	Irgm1	1,49	1,33E-02
	Isg15	2,17	1,42E-08
	Keg1	1,38	7,33E-04
	Lgals3bp	1,31	1,15E-04
	Ly6c1	4,81	7,37E-07
	Ly6e	1,72	7,15E-16
	Mmd2	-5,89	2,62E-05
	Mx2	1,75	2,05E-04
	Nampt	1,33	2,68E-03
	Nqo1	2,11	1,95E-03
	Oas1b	3,19	2,93E-04
	Oasl1	1,90	1,90E-15
	Oasl2	3,91	2,68E-03
	Oat	-1,03	4,51E-02
	Orm3	2,27	1,63E-03
	P2ry2	1,72	1,79E-02
	Parp12	1,20	2,69E-03
	Parp14	1,48	1,33E-06

	Parp9	1,17	1,63E-02
	Phf11d	1,77	7,76E-04
	Ppfibp2	1,44	1,22E-02
	Rnf213	1,85	6,88E-12
	Rsad2	1,34	1,07E-02
	Shisa5	1,03	2,86E-02
	Slc39a4	2,68	1,64E-03
	Slfn8	2,97	1,04E-03
	Snx10	1,23	1,65E-03
	Tap1	1,50	4,24E-05
	Tm6sf2	-1,28	9,42E-04
	Tmem45a	2,56	4,65E-02
	Trim30a	1,99	2,88E-04
	Trim30d	2,05	2,40E-11
	Ubd	4,04	1,33E-02
	Xaf1	3,01	8,79E-03
	Zbp1	2,30	2,82E-04
	Znfx1	1,11	2,86E-02
**_Agl_$^{-/-}$ vs _Agl_$^{-/-}$, rapamycin + AAV9-LiMP-GDEov**	Ablim3	-1,22	3,86E-03
	Arntl	-2,25	2,44E-03
	Bcl2l1	-1,04	4,34E-02
	Cdkn1a	-3,84	1,79E-52
	Fzd1	2,24	3,86E-03
	Lad1	1,89	7,08E-03
	Map3k13	-1,51	1,64E-02
	Mapkapk2	-0,96	1,60E-02
	Ppargc1a	-2,08	5,89E-05
	Ppp1r3b	-1,46	1,16E-02
	Ppp1r3c	-1,49	1,45E-06
	Retreg1	-1,07	1,16E-02
	Sdhd	1,40	1,10E-03
	Tcim	-1,20	4,53E-02
	Tppp	2,29	4,53E-02
	Trp53inp1	-1,92	3,40E-02
	Tubb2a	-1,58	1,14E-03
	Zap70	-1,78	3,38E-02
**_Agl_$^{+/+}$ vs _Agl_$^{-/-}$ & _Agl_$^{-/-}$ vs _Agl_$^{-/-}$ AAV9-LiMP-GDEov**	Aox3	-1,16	3,20E-02
	Cyp17a1	2,86	4,49E-07
	Usp18	2,46	3,34E-06
**_Agl_$^{-/-}$ vs _Agl_$^{-/-}$ AAV9-LiMP-GDEov & _Agl_$^{-/-}$ vs _Agl_$^{-/-}$, rapamycin + AAV9-LiMP-GDEov**	Avpr1a	-2,28	3,35E-11
	Cd36	2,14	2,93E-04
	Chka	-1,76	2,72E-04
	Crybg2	2,82	1,33E-02
	Elovl3	-1,91	7,10E-03
	Gdf10	1,88	1,23E-02

	Ifi44	3,76	8,08E-29
	Igfbp2	-2,64	3,64E-02
	Irf7	2,72	5,02E-27
	Irgm2	2,02	9,35E-12
	Ly6a	6,63	1,02E-53
	Nupr1	-3,99	2,46E-03
	Ppp1r3g	-5,13	6,64E-03
	Psmb9	1,55	5,04E-05
	Rtp4	2,63	1,37E-18
	Stat1	1,55	8,95E-09
	Tifa	1,79	3,89E-04
	Trim12a	1,98	3,38E-04
	Tsc22d3	-1,28	1,71E-03
	Xbp1	1,12	9,62E-04
**Common**	Adcy1	3,90	3,34E-06
	Agl	-3,14	1,31E-11
	Lepr	6,20	2,23E-04

A negative log2foldChange means that this gene is under-expressed in the second term of the comparison (under-expressed in *Agl*$^{-/-}$ condition in untreated *Agl*$^{+/+}$ versus *Agl*$^{-/-}$ mice comparison).

Supplementary Table 8. List of gene ontology terms (GO) found in pairwise comparisons between different conditions in liver. GO terms identified among all pairwise comparisons: untreated *Agl*$^{+/+}$ and *Agl*$^{-/-}$, and *Agl*$^{-/-}$ mice treated with AAV9-LiMP-GDEov alone or AAV9-LiMP-GDEov combined with rapamycin. Only GO terms with a Benjamini and Hochberg adjusted p-value < 0.05 were considered significant.

Comparison	DEGs	ID	Description	p.adjust
Agl$^{-/-}$ vs *Agl*$^{+/+}$	up-regulated	GO:0046394	carboxylic acid biosynthetic process	1,53E-02
		GO:0016053	organic acid biosynthetic process	1,53E-02
		GO:0044283	small molecule biosynthetic process	1,71E-02
Agl$^{-/-}$ vs *Agl*$^{-/-}$, AAV9-LiMP-GDEov	up-regulated	GO:0035456	response to interferon-beta	1,57E-17
		GO:0048525	negative regulation of viral process	4,31E-14
		GO:0050792	regulation of viral process	1,90E-11
		GO:1903900	regulation of viral life cycle	2,87E-11
		GO:0045071	negative regulation of viral genome replication	2,06E-09
		GO:0071345	cellular response to cytokine stimulus	2,21E-09
		GO:0016032	viral process	4,53E-09
		GO:0034341	response to interferon-gamma	4,53E-09
		GO:0019058	viral life cycle	4,66E-09
		GO:0045088	regulation of innate immune response	3,88E-08
		GO:0002831	regulation of response to biotic stimulus	6,39E-08
		GO:0045069	regulation of viral genome replication	6,39E-08
		GO:0019079	viral genome replication	1,52E-07
		GO:0035634	response to stilbenoid	2,84E-07
		GO:0002833	positive regulation of response to biotic stimulus	5,42E-07
		GO:0019221	cytokine-mediated signaling pathway	5,44E-07
		GO:0045089	positive regulation of innate immune response	5,72E-07
		GO:1903901	negative regulation of viral life cycle	1,09E-06
		GO:0042742	defense response to bacterium	1,32E-06
		GO:0060760	positive regulation of response to cytokine stimulus	1,54E-06
		GO:0060333	interferon-gamma-mediated signaling pathway	6,44E-06
		GO:0031347	regulation of defense response	1,32E-05
		GO:0060337	type I interferon signaling pathway	1,36E-05
		GO:0071357	cellular response to type I interferon	1,36E-05
		GO:0060759	regulation of response to cytokine stimulus	1,75E-05
		GO:0001961	positive regulation of cytokine-mediated signaling pathway	1,78E-05
		GO:0046597	negative regulation of viral entry into host cell	2,13E-05
		GO:0034340	response to type I interferon	2,65E-05

		GO:0071346	cellular response to interferon-gamma	3,91E-05
		GO:0060330	regulation of response to interferon-gamma	5,17E-05
		GO:0060334	regulation of interferon-gamma-mediated signaling pathway	5,17E-05
		GO:0046596	regulation of viral entry into host cell	8,04E-05
		GO:0031349	positive regulation of defense response	8,75E-05
		GO:0001959	regulation of cytokine-mediated signaling pathway	1,15E-04
		GO:0002218	activation of innate immune response	1,59E-04
		GO:0052372	modulation by symbiont of entry into host	1,59E-04
		GO:0043903	regulation of biological process involved in symbiotic interaction	2,08E-04
		GO:0035455	response to interferon-alpha	2,50E-04
		GO:0042832	defense response to protozoan	3,09E-04
		GO:0050778	positive regulation of immune response	3,62E-04
		GO:0032103	positive regulation of response to external stimulus	3,62E-04
		GO:0001562	response to protozoan	3,62E-04
		GO:0002753	cytoplasmic pattern recognition receptor signaling pathway	3,97E-04
		GO:0044403	biological process involved in symbiotic interaction	6,10E-04
		GO:0006805	xenobiotic metabolic process	6,10E-04
		GO:0002764	immune response-regulating signaling pathway	8,69E-04
		GO:0140289	protein mono-ADP-ribosylation	1,46E-03
		GO:0046718	viral entry into host cell	1,80E-03
		GO:0009410	response to xenobiotic stimulus	1,84E-03
		GO:0095500	acetylcholine receptor signaling pathway	1,84E-03
		GO:0044409	entry into host	2,18E-03
		GO:1905145	cellular response to acetylcholine	2,89E-03
		GO:0062207	regulation of pattern recognition receptor signaling pathway	3,47E-03
		GO:0071466	cellular response to xenobiotic stimulus	3,67E-03
		GO:0031664	regulation of lipopolysaccharide-mediated signaling pathway	4,15E-03
		GO:0031663	lipopolysaccharide-mediated signaling pathway	4,32E-03
		GO:0010506	regulation of autophagy	4,51E-03
		GO:1905144	response to acetylcholine	4,81E-03
		GO:0010508	positive regulation of autophagy	4,95E-03
		GO:0002684	positive regulation of immune system process	5,02E-03
		GO:0032069	regulation of nuclease activity	5,51E-03
		GO:0034121	regulation of toll-like receptor signaling pathway	6,19E-03
		GO:0052126	movement in host environment	6,19E-03

		GO:0044790	suppression of viral release by host	6,19E-03
		GO:0032481	positive regulation of type I interferon production	7,63E-03
		GO:0070534	protein K63-linked ubiquitination	7,63E-03
		GO:0098926	postsynaptic signal transduction	8,11E-03
		GO:0019373	epoxygenase P450 pathway	1,03E-02
		GO:0098586	cellular response to virus	1,13E-02
		GO:0051701	biological process involved in interaction with host	1,23E-02
		GO:0006471	protein ADP-ribosylation	1,42E-02
		GO:0050829	defense response to Gram-negative bacterium	1,42E-02
		GO:0001817	regulation of cytokine production	1,53E-02
		GO:0060338	regulation of type I interferon-mediated signaling pathway	1,56E-02
		GO:0006914	autophagy	2,51E-02
		GO:0061919	process utilizing autophagic mechanism	2,51E-02
		GO:0032728	positive regulation of interferon-beta production	2,73E-02
		GO:0001816	cytokine production	2,83E-02
		GO:0001676	long-chain fatty acid metabolic process	2,98E-02
		GO:0032479	regulation of type I interferon production	3,10E-02
		GO:0071396	cellular response to lipid	3,10E-02
		GO:0035457	cellular response to interferon-alpha	3,19E-02
		GO:0061635	regulation of protein complex stability	3,19E-02
		GO:0002224	toll-like receptor signaling pathway	3,29E-02
		GO:0032606	type I interferon production	3,59E-02
		GO:0044546	NLRP3 inflammasome complex assembly	3,70E-02
		GO:0140632	inflammasome complex assembly	3,70E-02
		GO:1900225	regulation of NLRP3 inflammasome complex assembly	3,70E-02
		GO:0031648	protein destabilization	4,23E-02
		GO:0018410	C-terminal protein amino acid modification	4,29E-02
		GO:0070431	nucleotide-binding oligomerization domain containing 2 signaling pathway	4,29E-02
	down-regulated	GO:0006633	fatty acid biosynthetic process	2,87E-02
		GO:0032793	positive regulation of CREB transcription factor activity	2,87E-02
		GO:0042761	very long-chain fatty acid biosynthetic process	2,87E-02
		GO:0072330	monocarboxylic acid biosynthetic process	2,87E-02
		GO:0032787	monocarboxylic acid metabolic process	2,87E-02
		GO:0008610	lipid biosynthetic process	2,87E-02
		GO:0030497	fatty acid elongation	2,87E-02
		GO:0006631	fatty acid metabolic process	3,82E-02

		GO:0046889	positive regulation of lipid biosynthetic process	4,98E-02
Agl$^{-/-}$ vs *Agl*$^{-/-}$, rapamycin + AAV9-LiMP-GDEov	up-regulated	GO:0007606	sensory perception of chemical stimulus	3,16E-02
		GO:0010742	macrophage derived foam cell differentiation	3,37E-02
		GO:0033993	response to lipid	3,37E-02
		GO:0090077	foam cell differentiation	3,49E-02
	down-regulated	GO:0010675	regulation of cellular carbohydrate metabolic process	1,42E-04
		GO:0006109	regulation of carbohydrate metabolic process	2,49E-04
		GO:0043470	regulation of carbohydrate catabolic process	9,08E-04
		GO:0043467	regulation of generation of precursor metabolites and energy	9,08E-04
		GO:0044262	cellular carbohydrate metabolic process	1,04E-03
		GO:0005977	glycogen metabolic process	1,04E-03
		GO:0006073	cellular glucan metabolic process	1,04E-03
		GO:0044042	glucan metabolic process	1,04E-03
		GO:0006112	energy reserve metabolic process	1,04E-03
		GO:0015980	energy derivation by oxidation of organic compounds	1,04E-03
		GO:0005979	regulation of glycogen biosynthetic process	1,04E-03
		GO:0010962	regulation of glucan biosynthetic process	1,04E-03
		GO:0044264	cellular polysaccharide metabolic process	1,04E-03
		GO:0005976	polysaccharide metabolic process	1,24E-03
		GO:0032885	regulation of polysaccharide biosynthetic process	1,39E-03
		GO:0070873	regulation of glycogen metabolic process	1,45E-03
		GO:0043255	regulation of carbohydrate biosynthetic process	1,45E-03
		GO:0032881	regulation of polysaccharide metabolic process	1,92E-03
		GO:1903580	positive regulation of ATP metabolic process	1,97E-03
		GO:0042752	regulation of circadian rhythm	1,97E-03
		GO:0005978	glycogen biosynthetic process	2,19E-03
		GO:0009250	glucan biosynthetic process	2,19E-03
		GO:0006091	generation of precursor metabolites and energy	3,07E-03
		GO:0120161	regulation of cold-induced thermogenesis	3,07E-03
		GO:0106106	cold-induced thermogenesis	3,14E-03
		GO:0010677	negative regulation of cellular carbohydrate metabolic process	3,14E-03
		GO:0033692	cellular polysaccharide biosynthetic process	3,14E-03
		GO:0010906	regulation of glucose metabolic process	3,23E-03
		GO:0016052	carbohydrate catabolic process	3,37E-03
		GO:0000271	polysaccharide biosynthetic process	3,43E-03

		GO:0045912	negative regulation of carbohydrate metabolic process	3,43E-03
		GO:1990845	adaptive thermogenesis	3,43E-03
		GO:0001659	temperature homeostasis	4,29E-03
		GO:0034637	cellular carbohydrate biosynthetic process	4,29E-03
		GO:0062013	positive regulation of small molecule metabolic process	4,46E-03
		GO:0005975	carbohydrate metabolic process	5,10E-03
		GO:0016051	carbohydrate biosynthetic process	5,63E-03
		GO:0043471	regulation of cellular carbohydrate catabolic process	6,81E-03
		GO:1900542	regulation of purine nucleotide metabolic process	7,41E-03
		GO:0006140	regulation of nucleotide metabolic process	7,64E-03
		GO:0007623	circadian rhythm	8,54E-03
		GO:0030810	positive regulation of nucleotide biosynthetic process	8,63E-03
		GO:1900373	positive regulation of purine nucleotide biosynthetic process	8,63E-03
		GO:2001171	positive regulation of ATP biosynthetic process	8,63E-03
		GO:0050671	positive regulation of lymphocyte proliferation	9,71E-03
		GO:0045820	negative regulation of glycolytic process	9,75E-03
		GO:0120162	positive regulation of cold-induced thermogenesis	9,76E-03
		GO:0032946	positive regulation of mononuclear cell proliferation	1,00E-02
		GO:0006006	glucose metabolic process	1,02E-02
		GO:0005980	glycogen catabolic process	1,14E-02
		GO:0044247	cellular polysaccharide catabolic process	1,14E-02
		GO:1901857	positive regulation of cellular respiration	1,14E-02
		GO:0043523	regulation of neuron apoptotic process	1,14E-02
		GO:0062014	negative regulation of small molecule metabolic process	1,16E-02
		GO:1903578	regulation of ATP metabolic process	1,19E-02
		GO:0000272	polysaccharide catabolic process	1,21E-02
		GO:0009251	glucan catabolic process	1,21E-02
		GO:0048145	regulation of fibroblast proliferation	1,27E-02
		GO:1900543	negative regulation of purine nucleotide metabolic process	1,32E-02
		GO:0070665	positive regulation of leukocyte proliferation	1,33E-02
		GO:0048144	fibroblast proliferation	1,36E-02
		GO:0045980	negative regulation of nucleotide metabolic process	1,40E-02
		GO:0019318	hexose metabolic process	1,40E-02
		GO:0048511	rhythmic process	1,40E-02
		GO:2001169	regulation of ATP biosynthetic process	1,51E-02

		GO:0051402	neuron apoptotic process	1,53E-02
		GO:0008630	intrinsic apoptotic signaling pathway in response to DNA damage	1,54E-02
		GO:1904019	epithelial cell apoptotic process	1,63E-02
		GO:0005996	monosaccharide metabolic process	1,63E-02
		GO:1903530	regulation of secretion by cell	1,63E-02
		GO:0030808	regulation of nucleotide biosynthetic process	1,67E-02
		GO:1900371	regulation of purine nucleotide biosynthetic process	1,67E-02
		GO:0048147	negative regulation of fibroblast proliferation	1,98E-02
		GO:0071480	cellular response to gamma radiation	2,13E-02
		GO:0007519	skeletal muscle tissue development	2,17E-02
		GO:0051046	regulation of secretion	2,22E-02
		GO:0043524	negative regulation of neuron apoptotic process	2,24E-02
		GO:0060538	skeletal muscle organ development	2,29E-02
		GO:0045981	positive regulation of nucleotide metabolic process	2,29E-02
		GO:1900544	positive regulation of purine nucleotide metabolic process	2,29E-02
		GO:1903579	negative regulation of ATP metabolic process	2,29E-02
		GO:0009152	purine ribonucleotide biosynthetic process	2,36E-02
		GO:0097009	energy homeostasis	2,41E-02
		GO:0009895	negative regulation of catabolic process	2,50E-02
		GO:0044275	cellular carbohydrate catabolic process	2,50E-02
		GO:1901861	regulation of muscle tissue development	2,50E-02
		GO:0006164	purine nucleotide biosynthetic process	2,55E-02
		GO:0043068	positive regulation of programmed cell death	2,56E-02
		GO:1901214	regulation of neuron death	2,64E-02
		GO:0050670	regulation of lymphocyte proliferation	2,71E-02
		GO:0071478	cellular response to radiation	2,71E-02
		GO:0009260	ribonucleotide biosynthetic process	2,78E-02
		GO:0072522	purine-containing compound biosynthetic process	2,78E-02
		GO:0009150	purine ribonucleotide metabolic process	2,78E-02
		GO:0032944	regulation of mononuclear cell proliferation	2,87E-02
		GO:0042771	intrinsic apoptotic signaling pathway in response to DNA damage by p53 class mediator	3,09E-02

		GO:0046390	ribose phosphate biosynthetic process	3,09E-02
		GO:0006163	purine nucleotide metabolic process	3,13E-02
		GO:0035914	skeletal muscle cell differentiation	3,14E-02
		GO:0045923	positive regulation of fatty acid metabolic process	3,14E-02
		GO:0150076	neuroinflammatory response	3,14E-02
		GO:0009259	ribonucleotide metabolic process	3,27E-02
		GO:0060537	muscle tissue development	3,28E-02
		GO:0010942	positive regulation of cell death	3,30E-02
		GO:0006110	regulation of glycolytic process	3,37E-02
		GO:0070997	neuron death	3,37E-02
		GO:0040008	regulation of growth	3,43E-02
		GO:2000300	regulation of synaptic vesicle exocytosis	3,43E-02
		GO:0070663	regulation of leukocyte proliferation	3,43E-02
		GO:0019693	ribose phosphate metabolic process	3,43E-02
		GO:0048662	negative regulation of smooth muscle cell proliferation	3,56E-02
		GO:0072521	purine-containing compound metabolic process	3,60E-02
		GO:0051090	regulation of DNA-binding transcription factor activity	3,61E-02
		GO:0023061	signal release	3,82E-02
		GO:0010332	response to gamma radiation	4,18E-02
		GO:0048871	multicellular organismal homeostasis	4,25E-02
		GO:0032940	secretion by cell	4,28E-02
		GO:1901215	negative regulation of neuron death	4,34E-02
		GO:0006754	ATP biosynthetic process	4,36E-02
		GO:0043457	regulation of cellular respiration	4,36E-02
		GO:1904705	regulation of vascular associated smooth muscle cell proliferation	4,36E-02
		GO:0046651	lymphocyte proliferation	4,37E-02
		GO:0009165	nucleotide biosynthetic process	4,41E-02
		GO:1901293	nucleoside phosphate biosynthetic process	4,46E-02
		GO:0032943	mononuclear cell proliferation	4,53E-02
		GO:0042102	positive regulation of T cell proliferation	4,53E-02
		GO:1990874	vascular associated smooth muscle cell proliferation	4,53E-02
		GO:0051251	positive regulation of lymphocyte activation	4,57E-02
		GO:0010823	negative regulation of mitochondrion organization	4,81E-02
		GO:0090398	cellular senescence	4,81E-02
		GO:0009206	purine ribonucleoside triphosphate biosynthetic process	4,95E-02
		GO:0009117	nucleotide metabolic process	4,95E-02
		GO:0051347	positive regulation of transferase activity	4,97E-02
	up-regulated	GO:0009617	response to bacterium	4,76E-05

Agl$^{-/-}$ vs *Agl*$^{-/-}$, rapamycin + AAV9-LiMP-GDEov & *Agl*$^{-/-}$ vs *Agl*$^{-/-}$, AAV9-LiMP-GDEov		GO:0043122	regulation of I-kappaB kinase/NF-kappaB signaling	1,31E-02
		GO:0071219	cellular response to molecule of bacterial origin	1,31E-02
		GO:0071222	cellular response to lipopolysaccharide	1,31E-02
		GO:0007249	I-kappaB kinase/NF-kappaB signaling	1,54E-02
		GO:0071216	cellular response to biotic stimulus	1,54E-02
		GO:0032496	response to lipopolysaccharide	2,02E-02
		GO:0002237	response to molecule of bacterial origin	2,27E-02
		GO:0051607	defense response to virus	2,29E-02
		GO:0140546	defense response to symbiont	2,29E-02
		GO:0043123	positive regulation of I-kappaB kinase/NF-kappaB signaling	2,55E-02
		GO:0032727	positive regulation of interferon-alpha production	2,88E-02
		GO:0009615	response to virus	3,16E-02
		GO:0032647	regulation of interferon-alpha production	3,21E-02
		GO:0032607	interferon-alpha production	3,37E-02
		GO:0045087	innate immune response	3,37E-02
		GO:0001819	positive regulation of cytokine production	3,61E-02
		GO:0035458	cellular response to interferon-beta	3,72E-02
		GO:0002221	pattern recognition receptor signaling pathway	4,20E-02
		GO:0050776	regulation of immune response	4,24E-02
	down-regulated	GO:0062012	regulation of small molecule metabolic process	1,07E-04

Supplementary Table 9. Numbers of Infiltrating immune cells in the liver of *Agl*$^{-/-}$ mice. Statistical analyses were performed by a one-way ANOVA (n=6 mice per group). Data are shown as mean ± SD

	**Agl$^{+/+}$**	**Agl$^{-/-}$**	**p value**
**Leucocytes (CD45)**	759621 ± 267040	1615388 ± 621324	0,0152 *
**Lymphocytes T (CD3+)**	454536 ± 196853	681361 ± 228677	0,1320 *ns*
**Neutrophiles (CD11b+ Gr1+)**	11757 ± 2796	27044 ± 17476	0,0649 *ns*
**Macrophages (CD11b+ F4/80)**	37979 ± 9703	133085 ± 77397	0,0022 **
**Macrophages M1 (CD80+)**	485 ± 306	2548 ± 2613	0,0022 **
**Macrophages M2 (CD206+)**	21116 ± 8557	52904 ± 23236	0,0260 *
