# Supplementary material for: Synergism of dual AAV gene therapy and rapamycin rescues GSDIII phenotype in muscle and liver
Source: JCI Insight. 2024 May 16;9(11):e172614. doi: 10.1172/jci.insight.172614 (PMC11382881; doi:10.1172/jci.insight.172614)

Figure 1B **HEART**

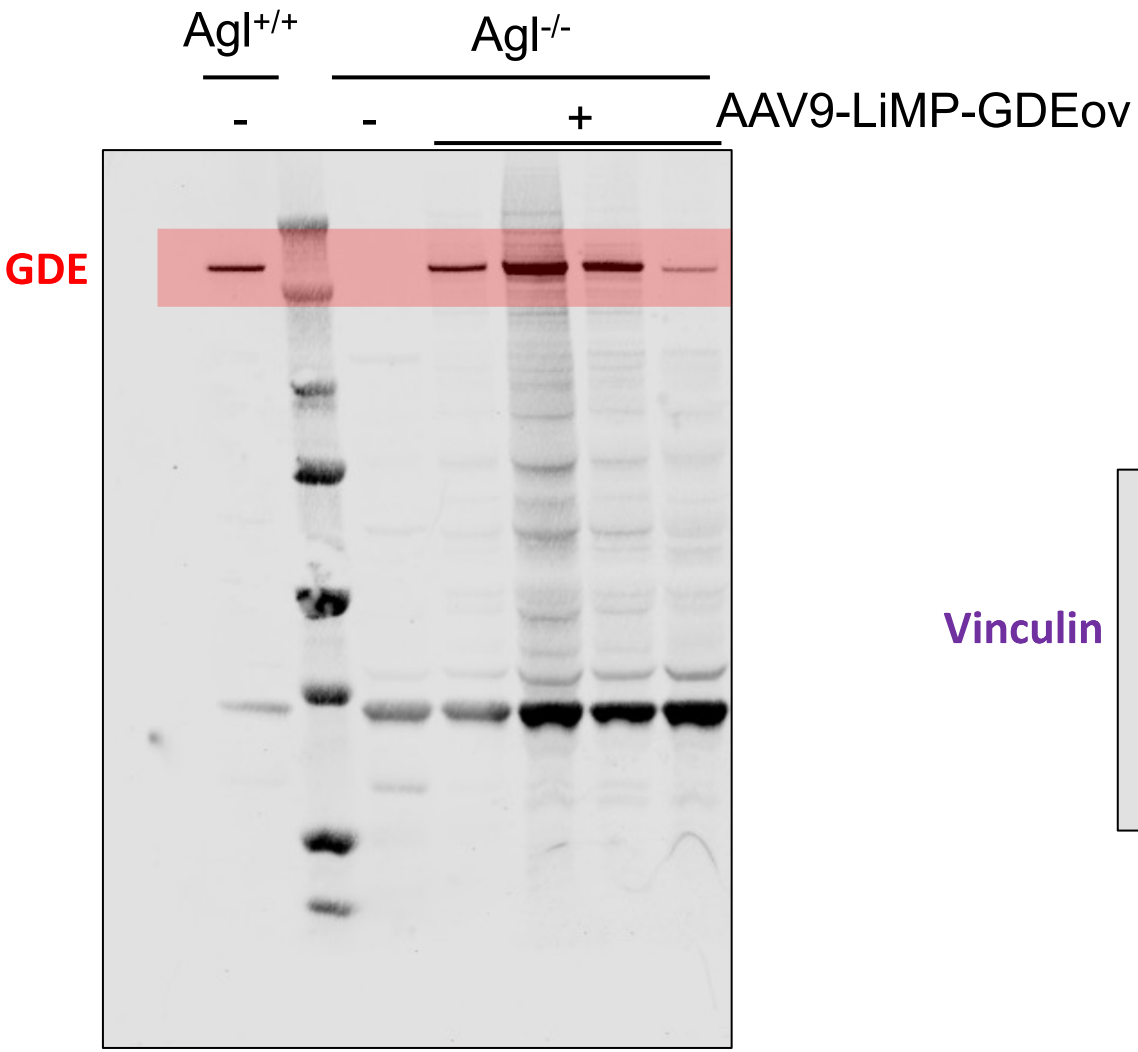

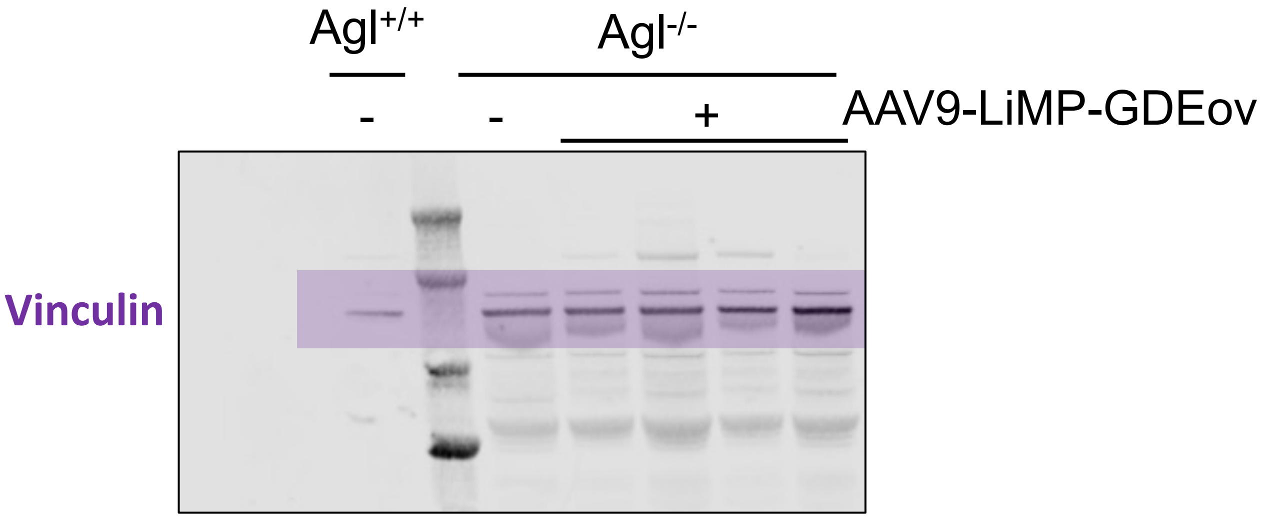

Figure 1B QUADRICEPS

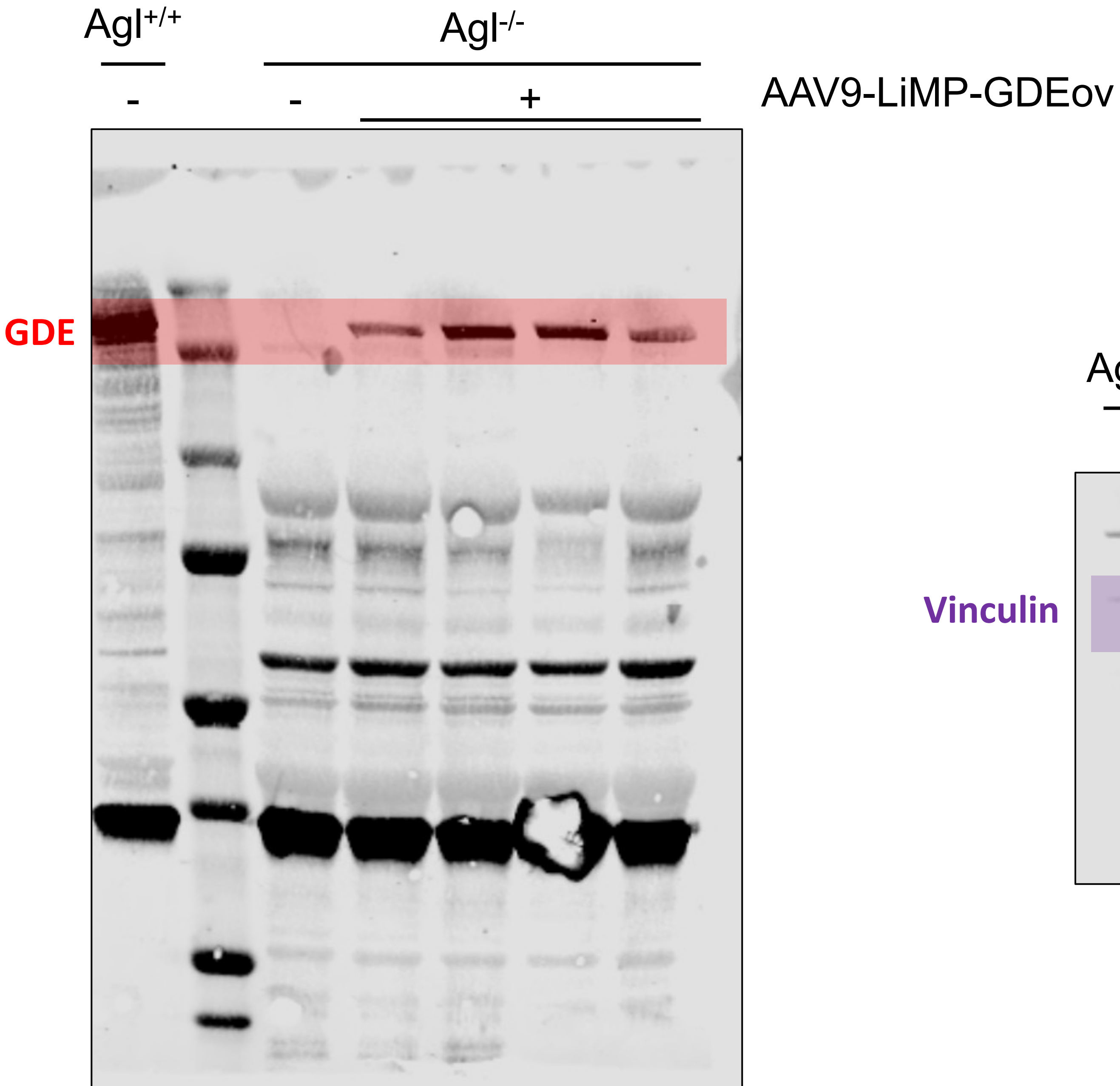

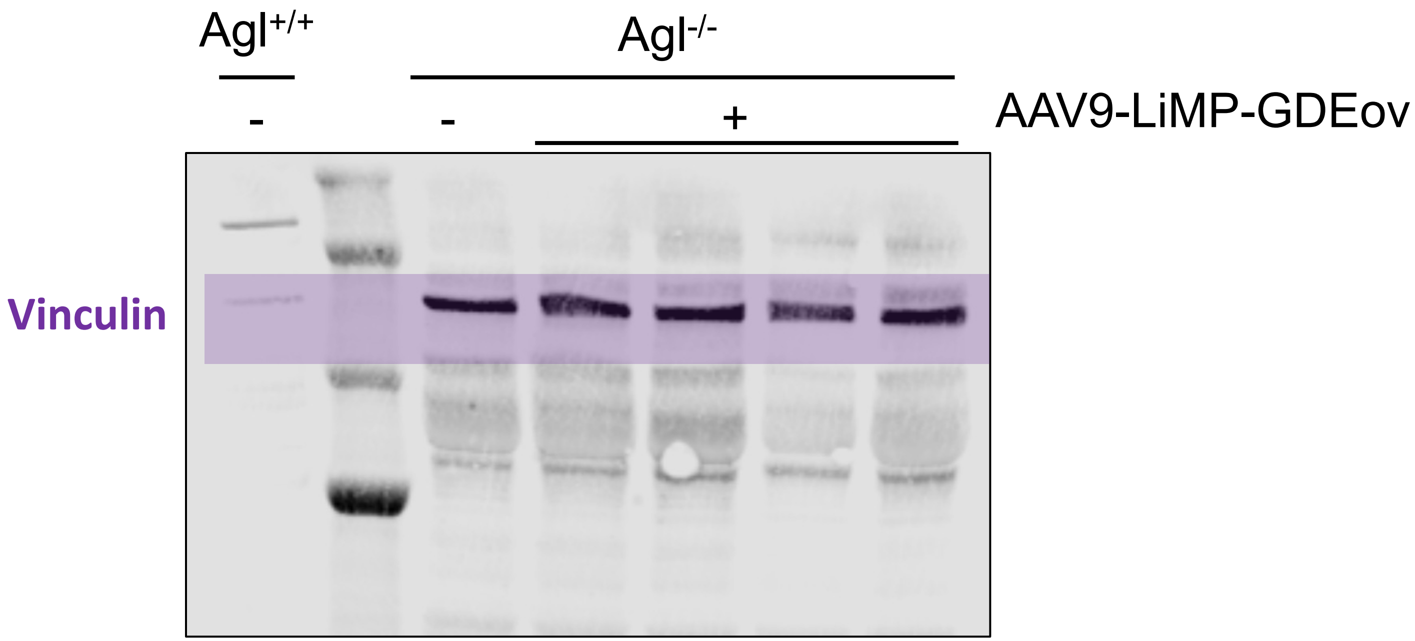

Figure 1E LIVER

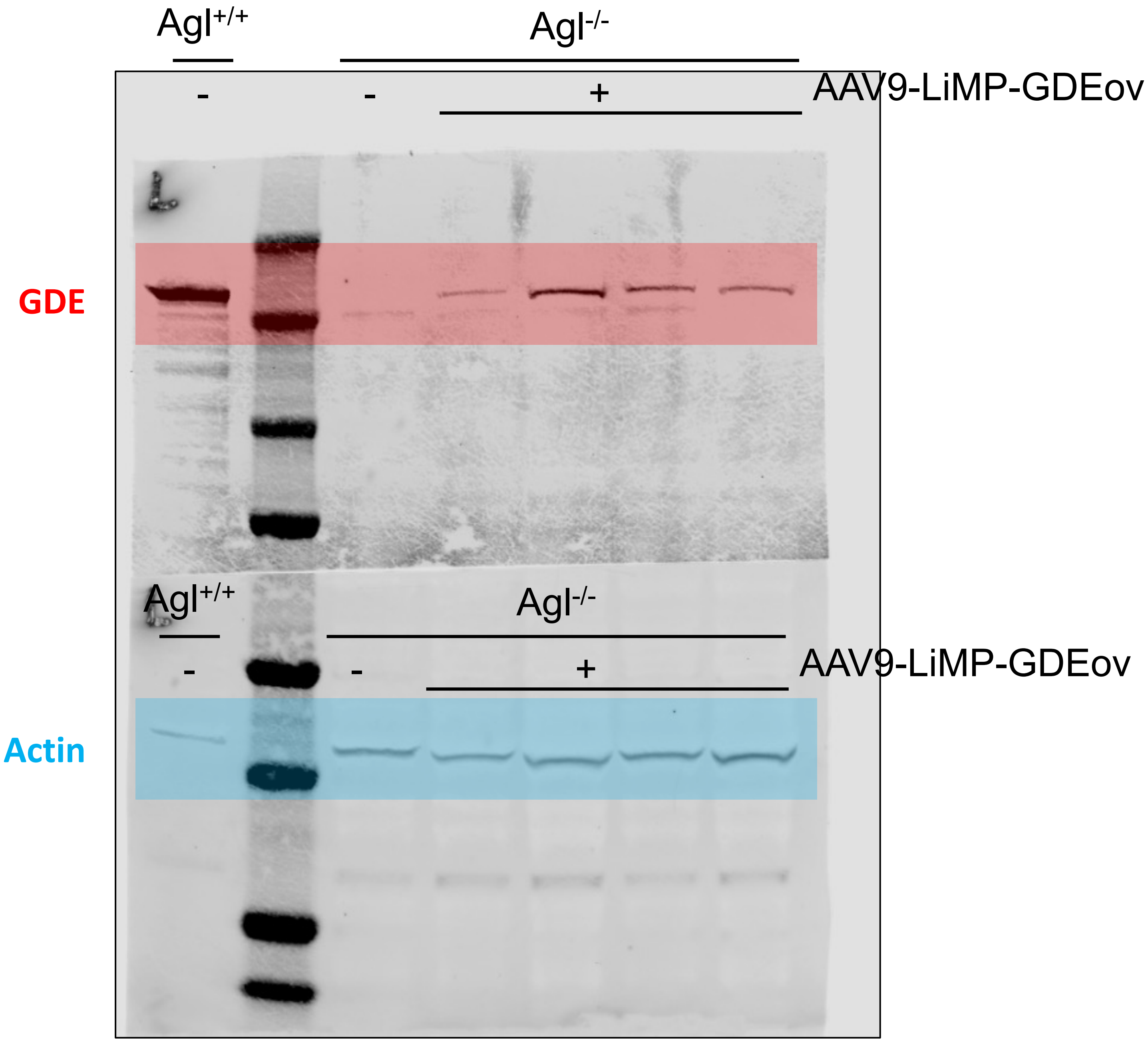

Figure 2G

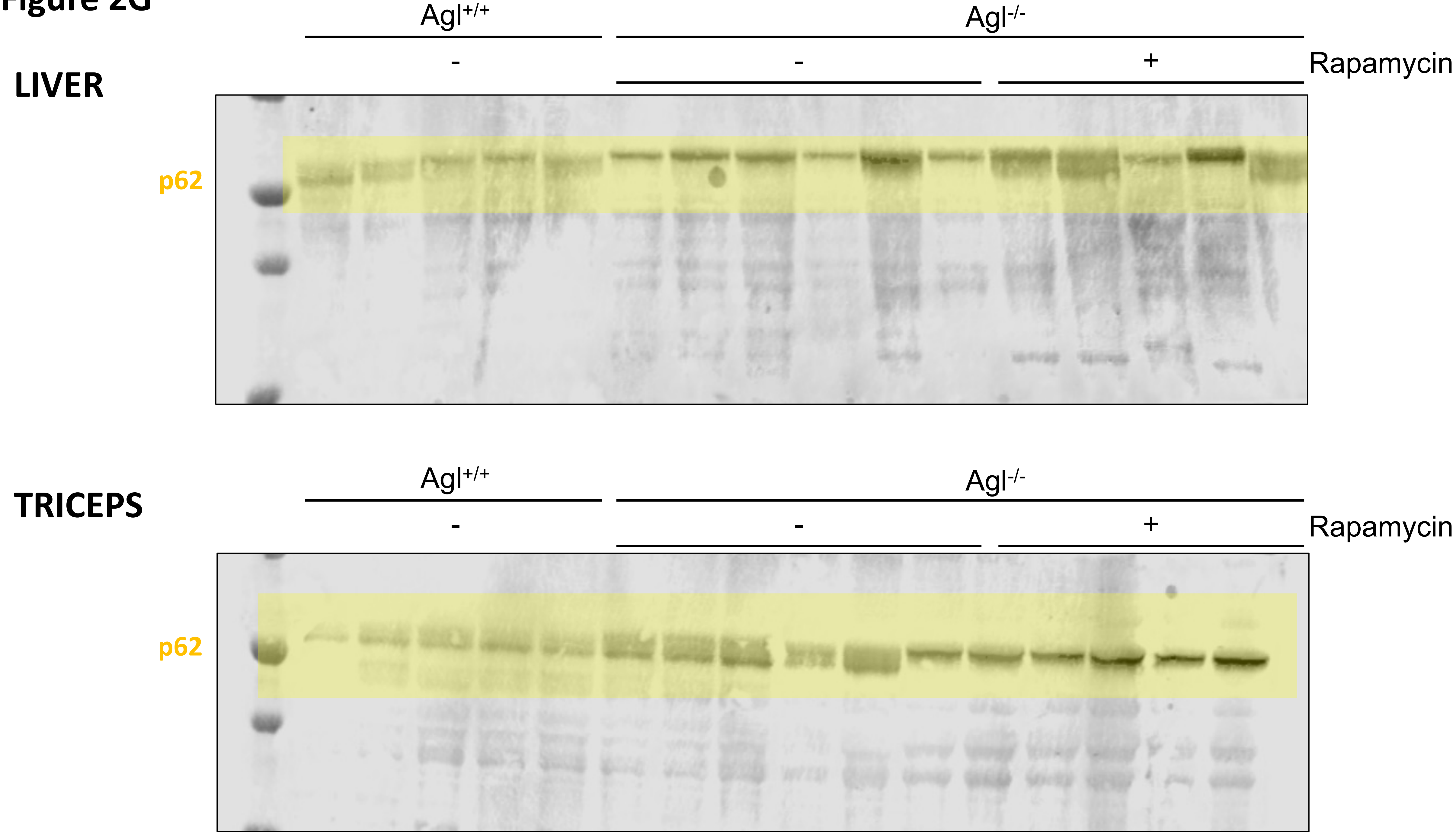

Figure 2G

LIVER

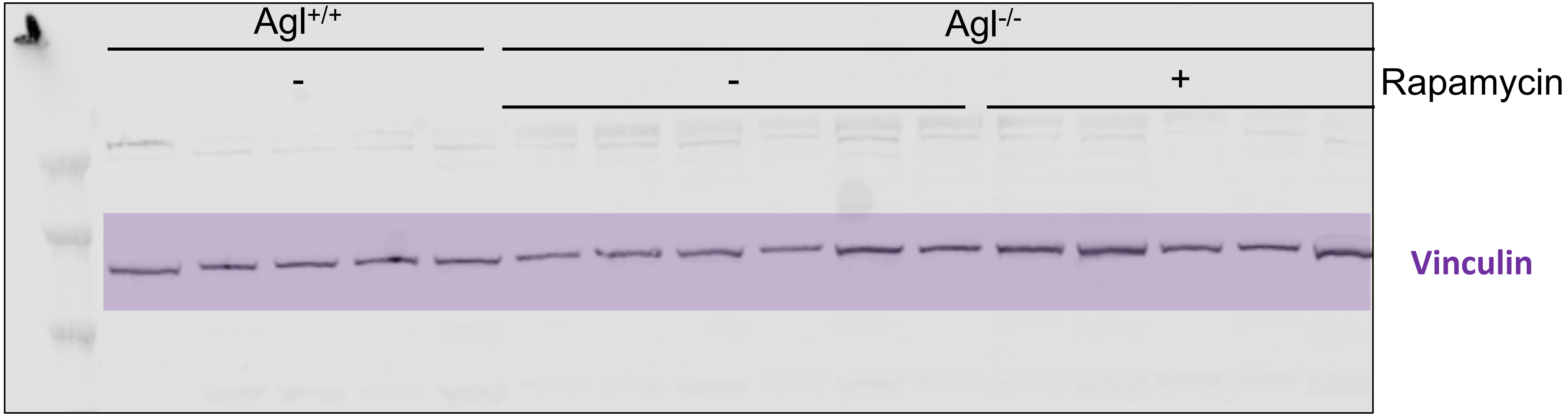

TRICEPS

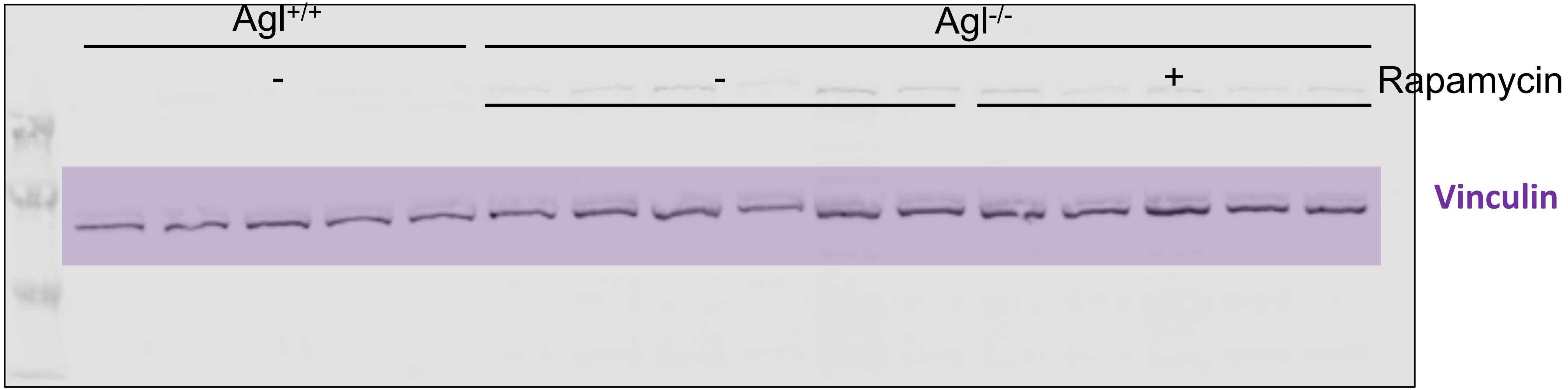

Supplementary Figure 2H

TRICEPS

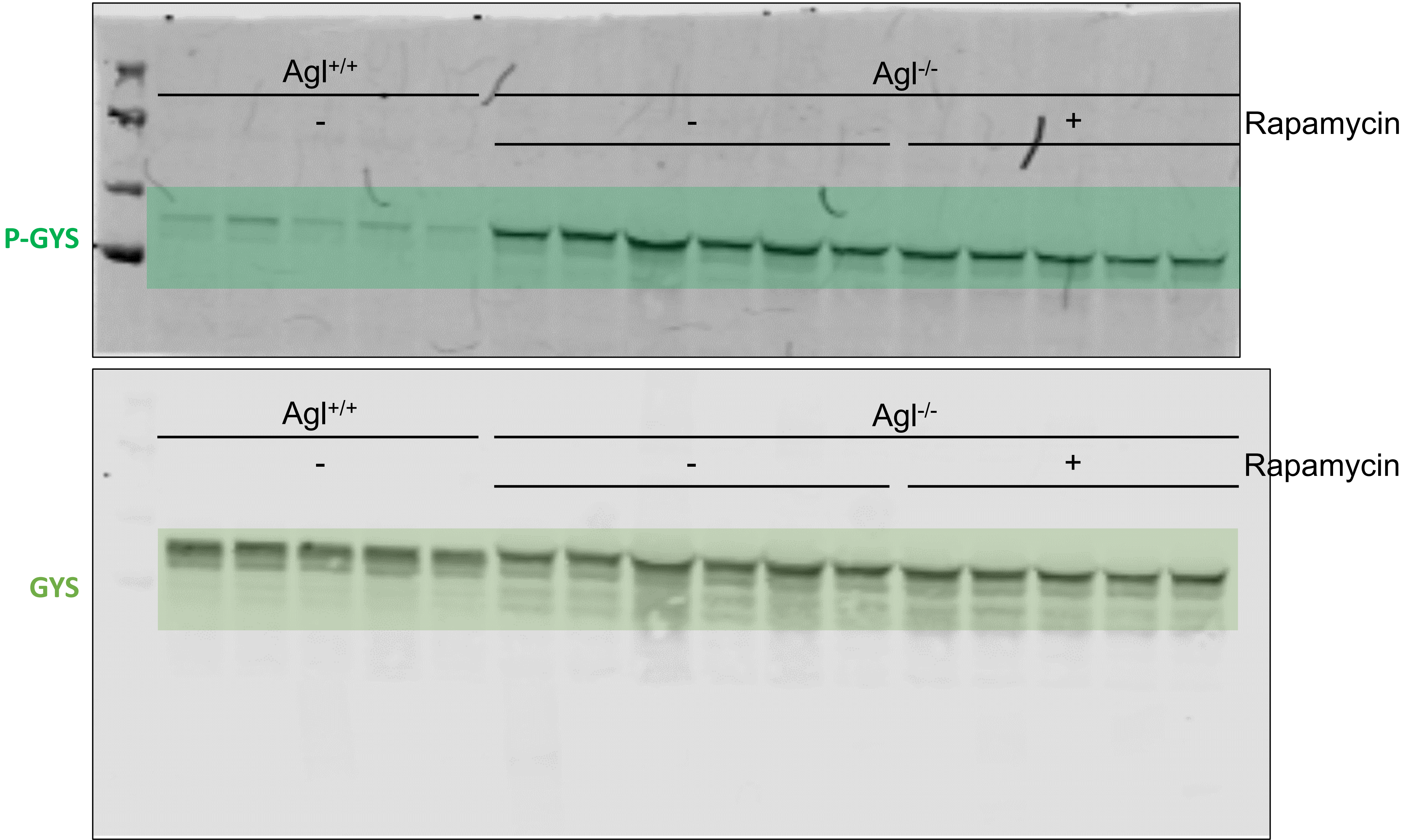

Figure 3B

LIVER

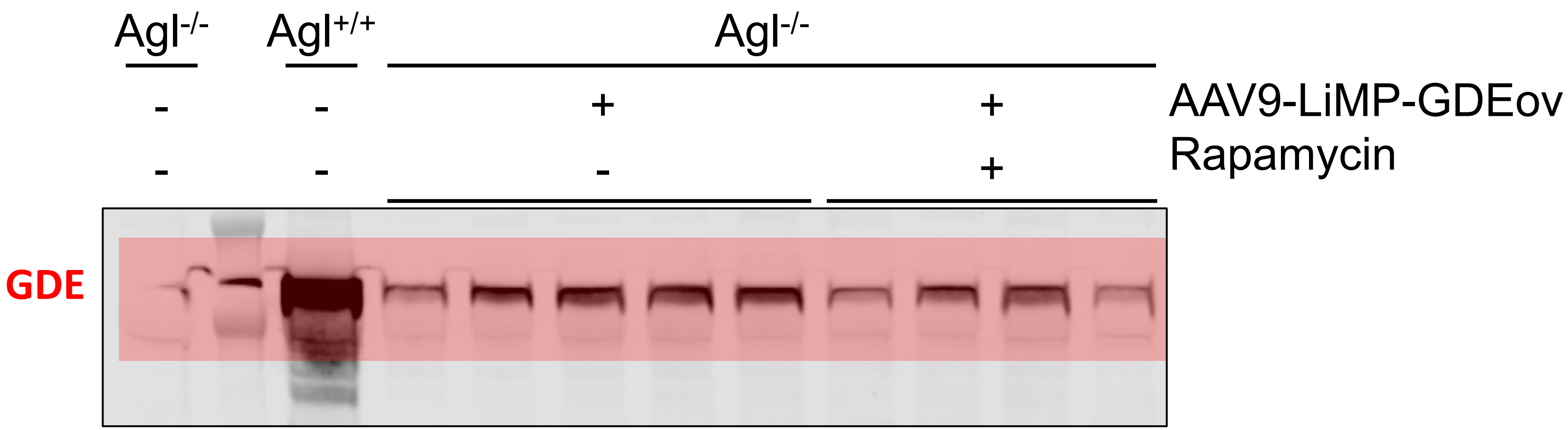

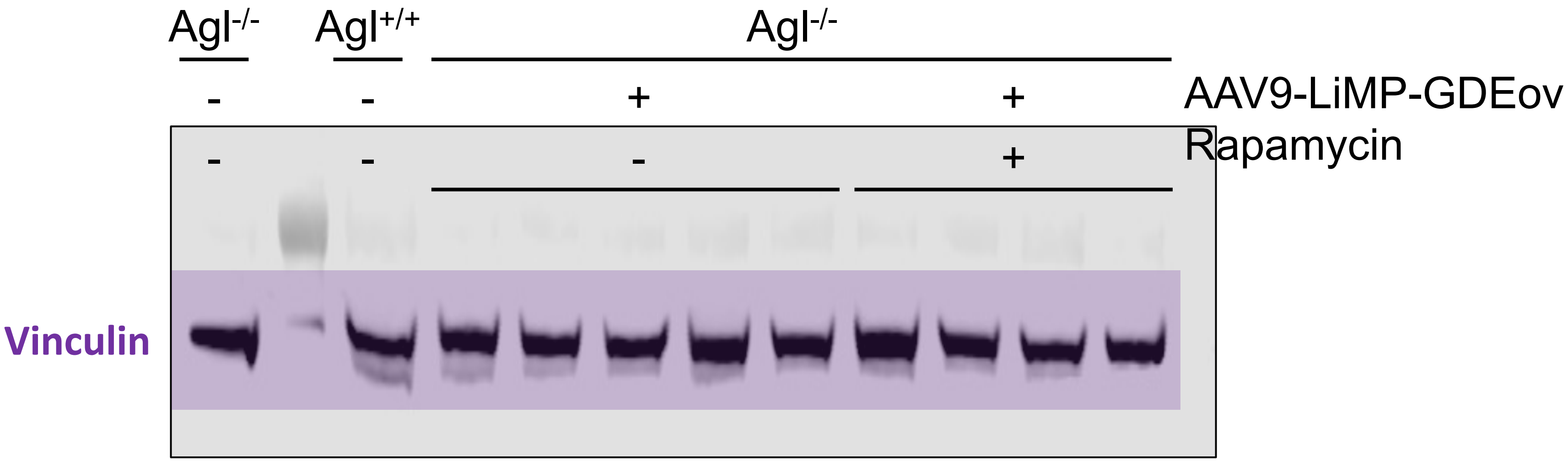

Figure 3D

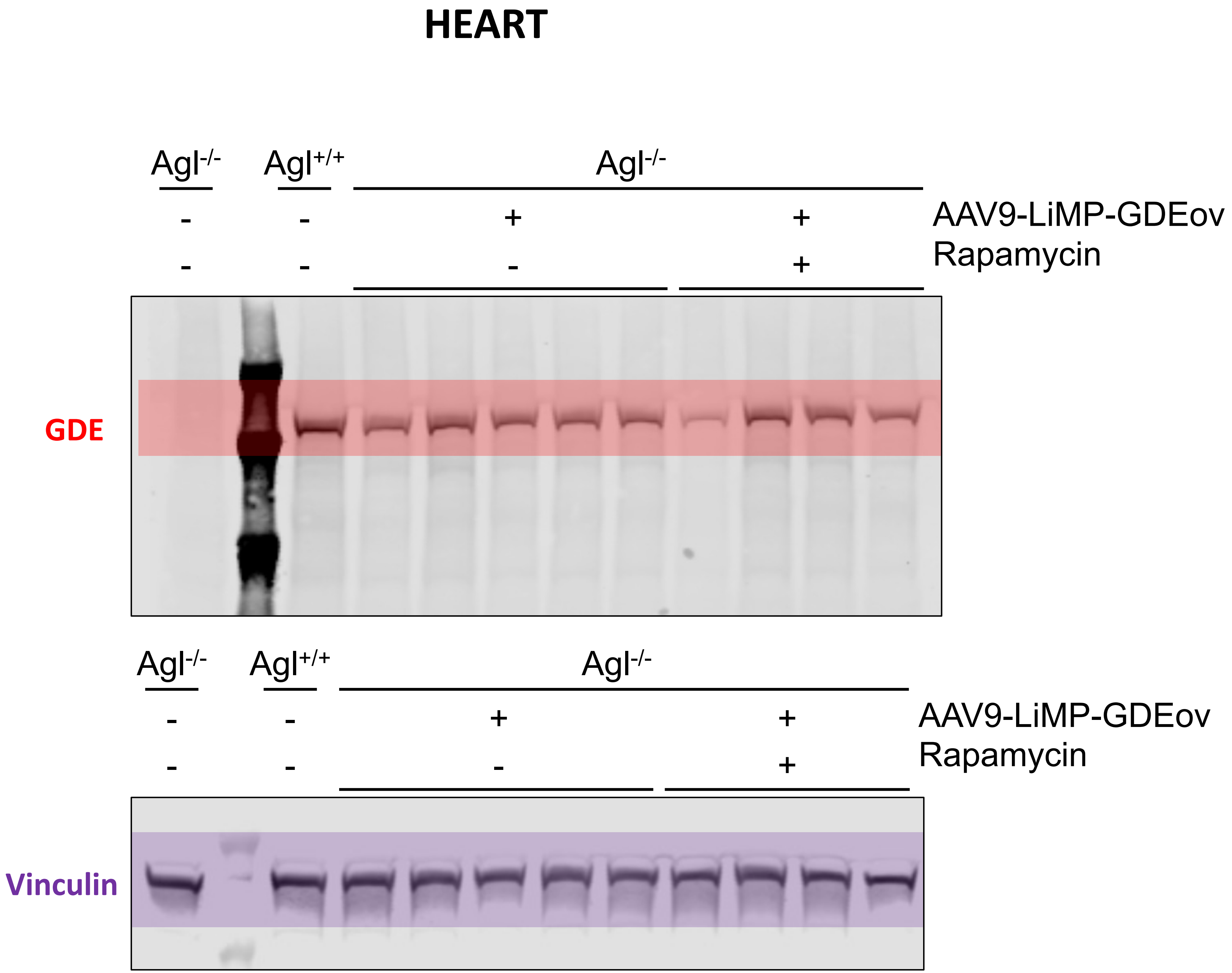
HEART
Agl-/-
Agl+/+
Agl-/-
AAV9-LiMP-GDEov
Rapamycin
GDE
Agl-/-
Agl+/+
Agl-/-
AAV9-LiMP-GDEov
Rapamycin
Vinculin

Figure 3D

QUADRICEPS

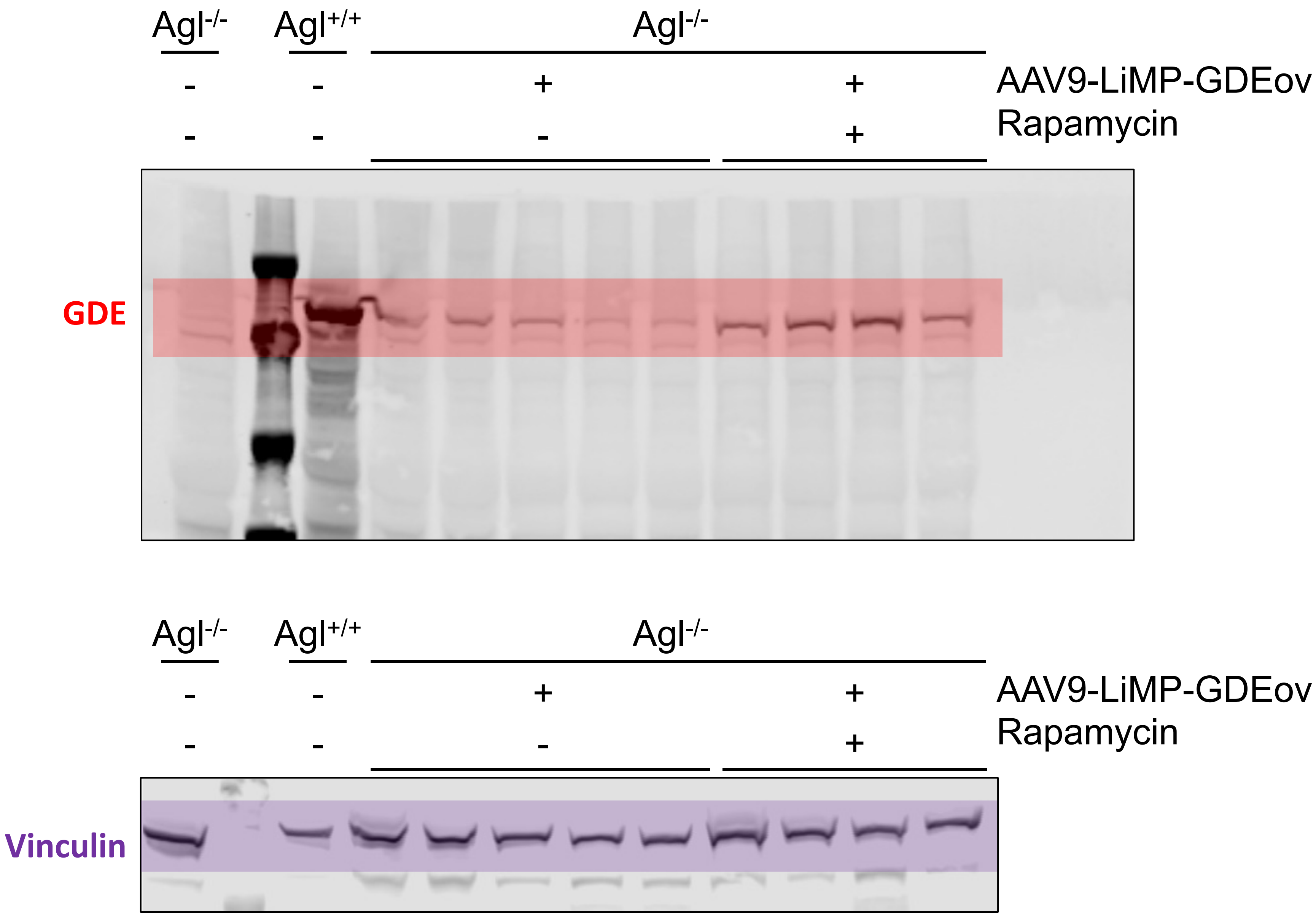

Figure 3D

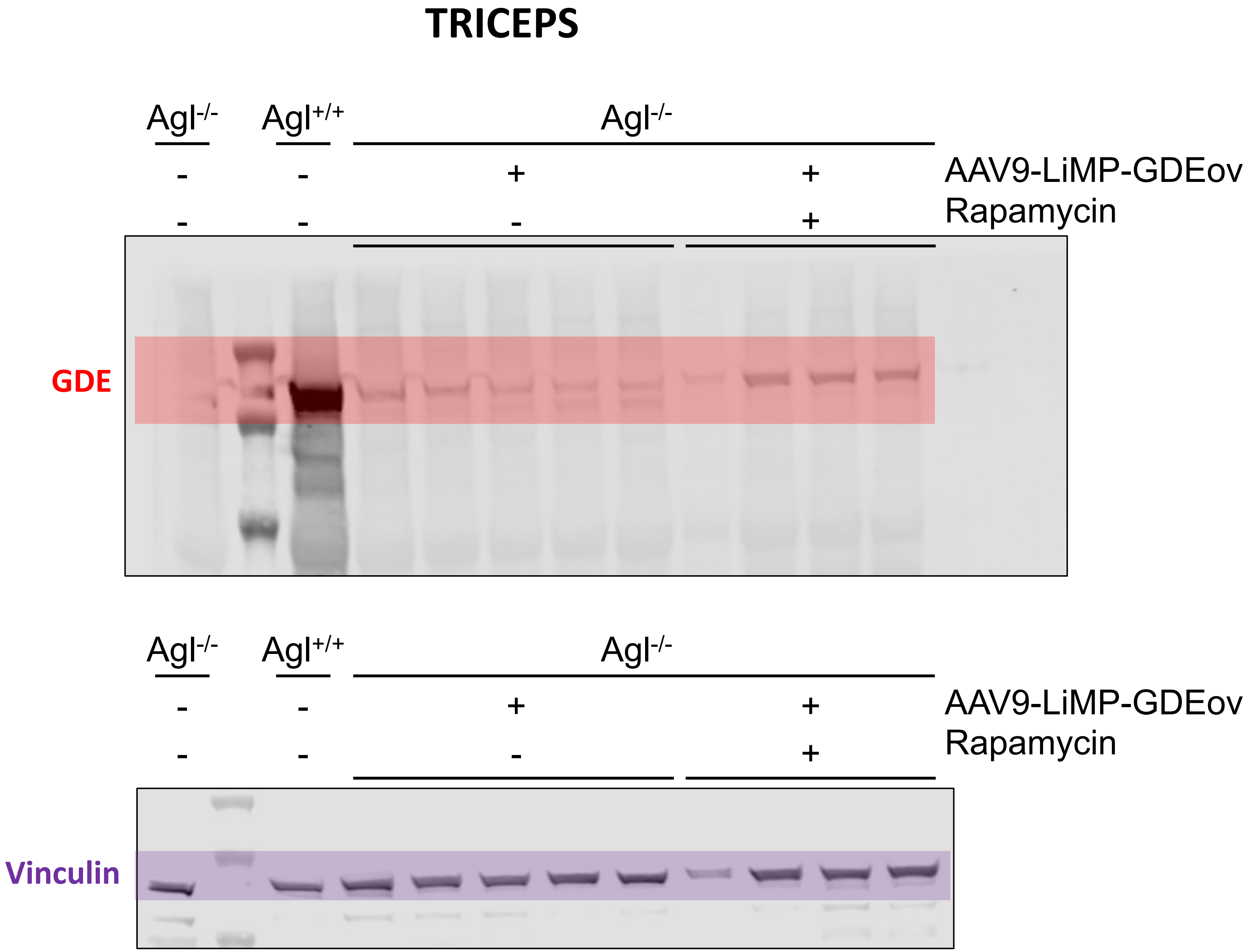
TRICEPS
Agl-/-
Agl+/+
Agl-/-
AAV9-LiMP-GDEov
Rapamycin
GDE
Agl-/-
Agl+/+
Agl-/-
AAV9-LiMP-GDEov
Rapamycin
Vinculin

Supplementary Figure 6B

QUADRICEPS

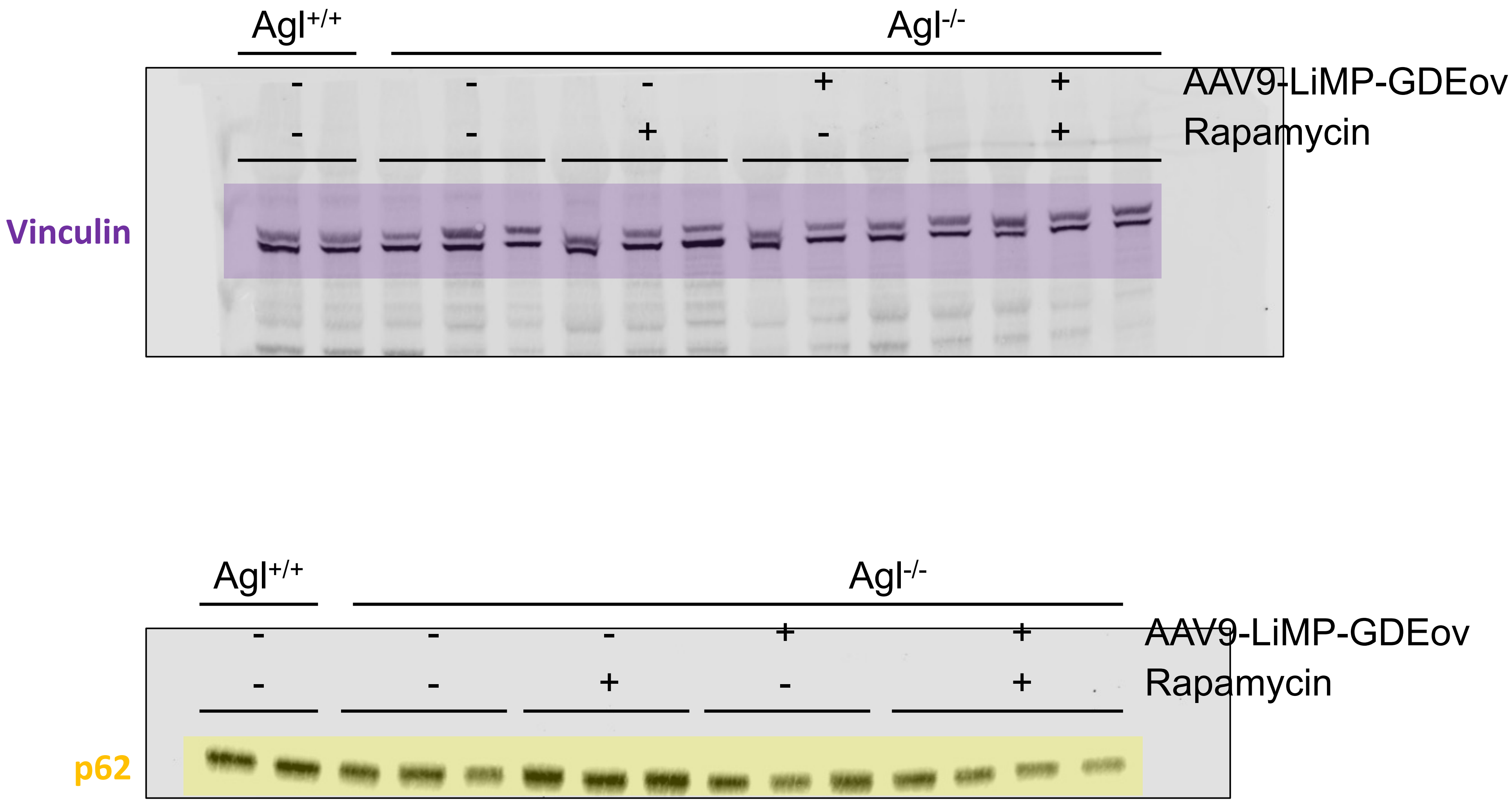

Supplementary Figure 6C

QUADRICEPS

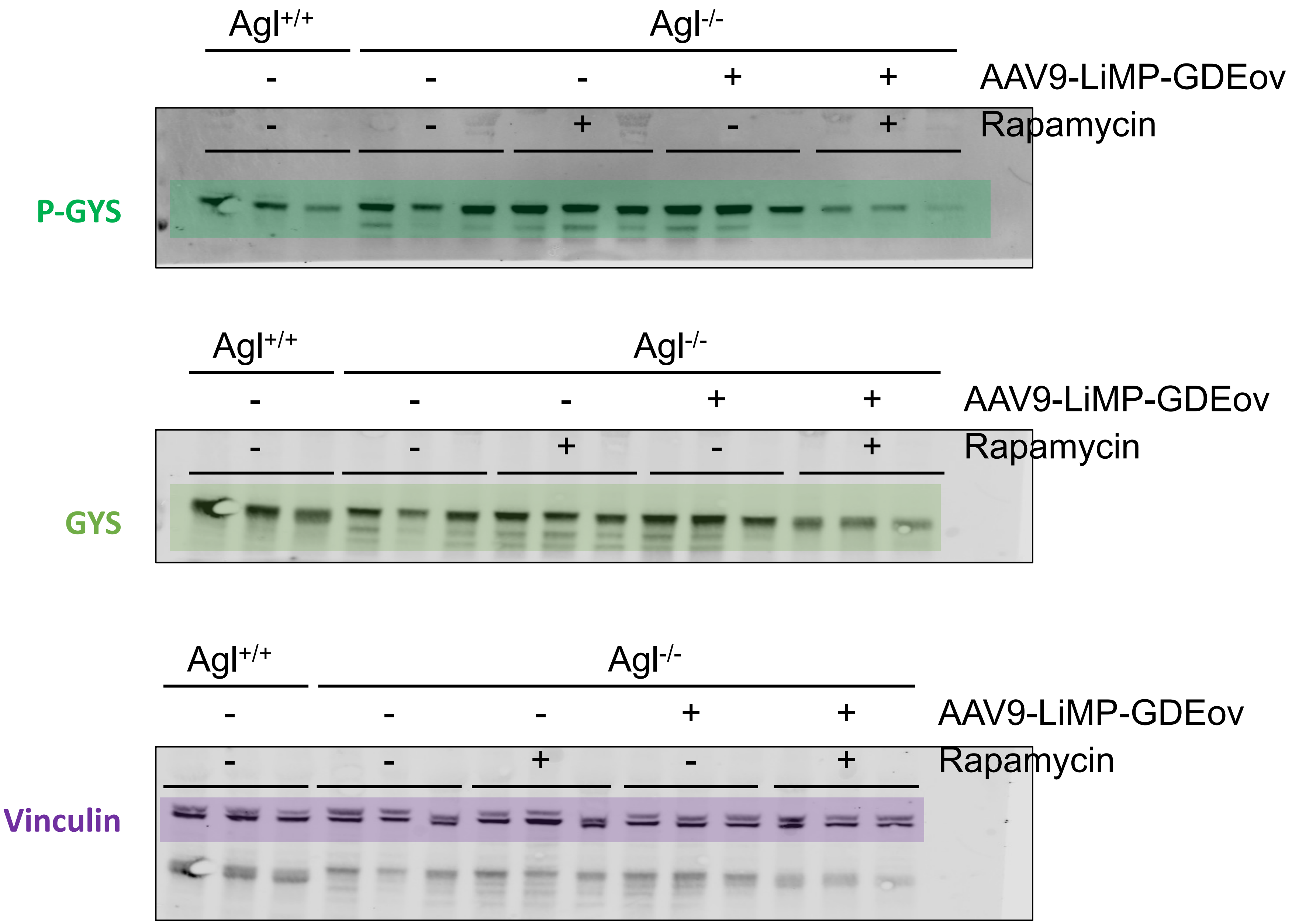

Supplement: Unedited blot and gel images [file jciinsight-9-172614-s154.pdf]
